# Characterization and Valorization of ‘Sulmona Red Garlic’ Peels and Small Bulbs

**DOI:** 10.3390/antiox11112088

**Published:** 2022-10-22

**Authors:** Alba Lasalvia, Francesco Cairone, Stefania Cesa, Alessandro Maccelli, Maria Elisa Crestoni, Luigi Menghini, Simone Carradori, Beatrice Marinacci, Marialucia Gallorini, Osama Elsallabi, Mirko Pesce, Antonia Patruno

**Affiliations:** 1 Department of Drug Chemistry and Technology, “Sapienza” University of Rome, Piazzale Aldo Moro 5, 00185 Rome, Italy; 2 Department of Pharmacy, “G. d’Annunzio” University of Chieti-Pescara, Via dei Vestini 31, 66100 Chieti, Italy; 3 Department of Medicine and Science of Aging, “G. d’Annunzio” University of Chieti-Pescara, Via dei Vestini 31, 66100 Chieti, Italy; 4 Department of Biosciences and Nutrizion, Karolinska Institutet, SE-141 57 Huddinge, Sweden

**Keywords:** Sulmona red garlic, metabolomics, FT-ICR mass spectrometry, color analysis, phytochemical composition, nitrosative stress, immunomodulation

## Abstract

‘Sulmona red garlic’ is an Italian variety characterized by a red tunica surrounding a white bulb. Red tunicae and non-commercial small bulbs are food wastes that must be studied for their added value. Hydroalcoholic extracts, obtained by separated inner and outer tunicae and peeled bulbs of small commercial ‘Sulmona red garlic’ bulbs, harvested at two different years, were first characterized with respect to their color, polyphenolic content, and antiradical activity. Then, an untargeted metabolic profile by means of electrospray ionization Fourier transform ion cyclotron resonance (ESI FT-ICR) mass spectrometry led to a comparative evaluation of the chemical diversity of six different samples. The study was completed by biological tests aiming to evaluate the associated health potential. Data on monocytes/macrophages showed good biocompatibility and a promising cytoprotective effect under oxidative stress conditions of all the extracts. At a molecular level, all the garlic extracts were able to downregulate the hydrogen peroxide-induced cyclooxygenase-2 and inducible nitric oxide synthase expression through the modulation of nuclear factor kappa-light-chain-enhancer of activated B cells (NF-ĸB) and peroxynitrite intracellular amounts, at different extents depending on the extract, the cell type, and the concentration. On the whole, data highlight an associated health potential of the extracts of this waste plant material both in terms of cytoprotection and of anti-inflammatory activity.

## 1. Introduction

Garlic (*Allium sativum* L.), belonging to the Amaryllidaceae family, represents one of the most diffuse aromatic and food herbs. It plays a prominent role among the traditionally employed herbal remedies, occupying a unique position in the history of folk medicine [1]. The increasing interest in functional foods and food bioactives has highlighted their potential involvement in the prevention of chronic and degenerative disorders [2]. Traditionally employed in the control of blood pressure, garlic extracts have been recently tested with success as antimicrobial, anti-inflammatory, anti-obesity, and anticancer agents. Garlic components could work synergistically and account for several different activities, such as hypolipidemic, antiplatelet, hepatoprotective, blood circulation, and immune enhancement [3]. The derived food supplements’ composition is largely influenced by the preparation and processing methods being crucial for selective biomolecule extraction. The complex chemistry of garlic bulbs should be interpreted as a self-protective mechanism related to the presence of metabolites with antimicrobial, antiradical, and antioxidant activities. Sulfur compounds consist of more than 20 biosynthetic derivatives from sulfur amino acids [4,5], mainly including cysteine sulphoxides, such as alliin and γ-glutamyl cysteine peptides. Mechanical processing of plant material is responsible for the release of the enzyme alliinase, which interacts with alliin and generates a plethora of degradation products such as allicin, ajoenes, vinyldithiins, and sulfides. The operative conditions during processing strongly influence the quali-quantitative degradation of metabolites and should be carefully considered when comparing the activity of different used products, such as bulbs, dry powder, essential oil, or oil extract [6]. The polyphenol fraction is mainly represented by phenolic acids and flavonoid compounds. The correlation of phenolic fractions to the antiradical/antioxidant ability of plant extracts was evaluated on many different garlic varieties.

2,2-Diphenyl-1-picrylhydrazyl (DPPH) assay, hydroxyl radical scavenging capacity, ferric ion reducing antioxidant power, cupric ion reducing antioxidant capacity, and metal chelating activity assay showed that samples with higher phenolic or flavonoid content had stronger protective effects [7]. Recent studies deal with several garlic cultivar and/or wild garlic bioactive compositions and the associated health-promoting effects [6,8], even related to the anti-COVID-19 (coronavirus disease) potential [9]. Some authors [10,11,12] focused on the garlic chemical composition and applied multivariate analyses to define a model for a geographical discrimination among different cultivars.

Indeed, ‘Sulmona red garlic’ identifies the commercial product obtained from *Allium sativum* ‘Rosso di Sulmona’ resulting characteristic for its geographic origin. The research of stable isotope was also proposed to discriminate among garlic samples cultivated in different Italian regions, showing that phytochemical and antioxidant properties were influenced by genetic, environmental and pedoclimatic factors [13,14]. A focus on these selected garlic samples confirmed direct effects of manufacturing process on the organosulphur composition, comparing methods, extraction solvents and operative conditions [15]. As for other food plants, many different varieties, ecotypes, and cultivars of garlic are defined deriving from the geographical origin or to the selective conservation of specific characteristics related to adaptation capabilities, productivity, yield, aroma, and others. ‘Rosso di Sulmona’ is a variety inserted into the list of the officially recognized commercial varieties of red garlic (DM 296/2009 delivered by Italian Ministry of Agricultural, Food and Forestal Policy). It represents the result of decades of agricultural selections operated in Peligna Valley (Abruzzo region, Italy) for the commercial production, according to the local pedoclimatic conditions. The crop is also recognized as a traditional agri-food product of Abruzzo Region and of the Italian cultural and agri-pastoral heritage (Italian Law n. 238/2016). Its identification is related to morphological and morphometric characteristic factors, while the chemical composition is not contemplated. The whole bulb is covered by a white membranous and dry tunica and once the bulb is divided, the presence of an inner reddish tunica surrounding each bulbil becomes evident. Intriguingly, this macroscopic evidence is related to the botanical name of the variety (red = rosso), without a demonstrated characteristic chemotype. In this context, experimental research was already performed to better characterize this peculiar Italian variety, including a multimethodological characterization of primary and secondary metabolites, pigments, and colors of the polar extracts [16], as well as pharmacological investigations confirming the neuroprotective effects in an ex vivo model of oxidative stress [7].

In the present work, an extensive investigation was conducted on commercial samples of ‘Sulmona red garlic’ by comparing chemical and biological properties of the smaller bulbs. The garlic tunicae, non-suitable for the market because generally discarded by the consumers, represents, on average, the 3.5% of the bulbs. This means that, considering a production of 6.5–15 million tons of garlic per year, as reported by Kallel and Chaabouny [17], a range of 230 and 520 thousand tons/year of discarded wastes are produced. In the light of these consideration, it becomes even clearer that the outer white tunica and the inner red tunica, represent waste products that deserve to be valorized. The quali-quantitative changes of hydroalcoholic extracts from samples, collected in two different years, were evaluated by color analysis, HPLC-DAD and ESI FT-ICR, already applied to investigate the pigment and polyphenol components besides the (moderately) polar fraction of garlic extracts, respectively [16], whereas the intrinsic antiradical activity was measured through DPPH assay. Then, we aimed to establish whether the garlic extracts displayed cytotoxicity performing classical biocompatibility tests ((3-(4,5-dimethylthiazol-2-yl)-5-(3-carboxymethoxyphenyl)-2-(4-sulfophenyl)-2*H*-tetrazolium) or MTS assay). Subsequently, our study focused on evaluating anti-inflammatory properties to extend the understanding of the health benefits of the novel extracts. Herein, we examined their effects, assessing the impact on inducible nitric oxide synthase (iNOS) and cyclooxygenase-2 (COX-2) expression and peroxynitrite generation on H_2_O_2_-induced cellular toxicity in undifferentiated (monocyte) and differentiated (macrophages) human immortalized monocyte-like cell line (THP-1), a well-established cell model for the immune-modulation study. A potential mechanism of action through effects on nuclear factor kappa-light-chain-enhancer of activated B cells (NF-kB) activation was investigated. Moreover, antimicrobial screening on three different bacterial species was also evaluated for the same extracts.

For the first time a multi-methodological analytical approach has been applied to the phytochemical and biological characterization of inner and outer tunicae and bulbs of ‘Sulmona red garlic’, a valuable Italian cultivar. Particularly, the untargeted methods showed a wide range of beneficial compounds as well as a promising anti-inflammatory activity.

## 2. Materials and Methods

Methanol and acetonitrile (HPLC-grade) were obtained from Merck (Milan, Italy). Reference compounds (reported in Supplementary Materials, Table S1) for HPLC analysis were purchased from Merck (Milan, Italy). All the solvents and chemical standards were analytical-grade products used without further purification.

### 2.1. Plant Material

Plant material consisted of bulbs of *Allium sativum* ‘Sulmona red’ collected from a common productive chain designated for commercialization at “Aglio D’Alessandro” company (Pratola Peligna, L’Aquila, Italy). The production is completely integrated into the company that manages directly all aspects related to farming, harvesting, and processing, according to good agricultural practice (global GAP certified). The external part of the whole bulb shows several outer layers that are the dry residuals of sheathing protective leaves surrounding the inner bulbils (commonly called cloves). Each bulbil presents a membranaceous coating derived from an inner sheath that wraps the edible part represented by the fleshy storage leaves. The external sheathing layers (outer tunica) present a white to gray color as in all garlic varieties, while the inner tunica (surrounding the bulbils) is characterized by red-purple speckles of different extents and intensities. Along with the processing to prepare the commercial products, a manual selection of only healthy bulbs is operated. The second step of selection is performed on the basis of bulb size and aspect in order to discriminate: the biggest and most vigorous as a first quality product, the medium (diameter >55 mm, with regular shape) representing the conventional commercial size, and the smaller or those with irregular shape are discarded as not suitable for commercialization. The latter group represents a waste product for the production of whole garlic bulbs but, at the same time, it represents a high-quality plant material that is discarded only for the general aspect; in fact, some companies use the smaller bulb to produce a second line of commercial products that are the hull bulbils. In order to support the implementation of the productive chain, the sampling of plant material for experimental research was performed on the bulbs considered waste products. For each year, a sample of approximately 4 kg (consisting of dozens of bulbs) was randomly selected from different bulks representative of the whole seasonal harvest. All bulbs of the sample were further selected to discard unhealthy ones and manually opened to carefully separate the outer white tunica, the inner red tunica, and the bulbils. The tunicae are already dry, and, to prevent degradation of the swollen bulbils, the samples were directly used for the extraction. Samples were collected in two different years (July 2020 and July 2021) in order to highlight the variability of the selected crop.

### 2.2. Hydroalcoholic Extraction

According to Garzoli et al. [18], with some modifications, 1 g of each sample (white tunica W, red tunica R, bulb B) was mixed with a hydroalcoholic mixture (10 mL of ethanol/5% acetic acid in water in the ratio 70:30, *v*/*v*), homogenized for 1 min (IKA Ultra-Turrax T-50 Homogenizer) and then extracted for 1 h, at room temperature, in the darkness and under stirring (Heidolph MR 2002, Heidolph Instruments GmbH & Co., Schwabach, Germany). The exhausted plant material was separated by filtration, and the liquid phase was taken to dryness by means of rotary evaporation. The obtained residue was dissolved in 2 mL of distilled water, frozen at −30 °C, and lyophilized (Lio5P, Padua, Italy). Obtained dry extracts were stored at −20 °C until analyzed or used for biological tests.

### 2.3. Colorimetric Analysis

After solubilization, extracts (W, R, B) were submitted to colorimetric CIEL*a*b* analysis with a colorimeter X-Rite MetaVue^®TM^ 140 (Grand Rapids, MI, USA), equipped as described in Recinella et al. [19].

### 2.4. HPLC-DAD Analysis

Samples of dry extracts (W, R, B) were weighed and dissolved in methanol. The resulting solutions were injected and analyzed with an HPLC-DAD (Perkin Elmer, Milan, Italy), equipped as described in Garzoli et al. [18]. Analyses were performed at 280 nm, 360 nm, and 520 nm on a Luna RP-18, 3 µm column, with a linear gradient consisting of acetonitrile and acidified water (5% formic acid), from 100% aqueous phase to 85% in 15 min, 85% to 55% in 30 min and 55% to 40% in 20 min, at a flow rate of 0.8 mL/min. Analytes were identified by comparing retention times and ultraviolet–visible spectroscopy (UV-Vis) absorption spectra to those of authentic standards (Table S1 and Figure S1).

### 2.5. Mass-Spectrometry-Based Metabolomics

Samples (W, R, B) have been analyzed by direct infusion electrospray ionization (ESI) mass spectrometry (MS). High-resolution mass analysis of each sample was carried out by using a Bruker BioApex Fourier transform ion cyclotron resonance (FT-ICR) [20]. A hydroalcoholic extract (1 mg) was diluted in 1 mL of H_2_O/CH_3_OH/CH_3_COOH (49:49:2 *v*/*v*/*v*), filtered through a 0.45 µm polypropylene Acrodisc (SigmaAldrich, Milan, Italy) syringe filter, diluted in methanol to a final concentration of 0.1 mg/L and directly injected into ESI source for MS analyses. The solutions were kept at −20 °C and analyzed within 24 h after final dilution. The latter solutions were added with either ammonia, 1% *v*/*v*, (ESI(-)) or formic acid, 1% *v*/*v*, (ESI(+)). All mass spectra were recorded in the *m*/*z* 100–1000 range (resolution of 650,000 at *m*/*z* 400) in three independent replicates, and the measurements were based on the “monoisotopic” ion. The univocal molecular formula has been gathered to several metabolites in the range of ±5 ppm by the free tool Mass TRIX (available online: http://masstrix3.helmholtz https://www.muenchen.de/masstrix3/index and accessed on 13 July 2022). Collision-induced dissociation (CID) experiments were carried out on mass-selected ions left to collide with He gas in a linear ion trap mass spectrometer LTQ XL (Thermo Fisher, Waltham, MA, USA). These experiments provide complementary information for peak identification, further verified in comparison with reference spectra obtained from the literature or specialized database, like Metlin (https://metlin.scripps.edu accessed on 20 June 2022). ESI parameter settings were: flow rate of 120 µL h^−1^, nebulizer gas pressure was set at 1.0 bar, the drying gas flow rate at 4.0 L min^−1^ at a temperature of 200 °C, and the capillary exit voltage at 200 V.

### 2.6. Evaluation of Antiradical Activity by DPPH Assay

Tests were performed according to Cairone et al. [21] using a UV/VIS Lambda 25 spectrophotometer Perkin Elmer (Waltham, MA, USA). A total of 0.5 mL of extract solutions (about 0.25 mg in 0.5 mL ethanol) were added to 2 mL of the DPPH ethanolic solution 100 µM and 0.5 mL of ethanol. A blank solution made with 2 mL of the same DPPH solution and 1 mL of ethanol was used. A calibration curve (y = 0.5171e − 3x; R^2^ = 0.9994), with gallic acid as a reference, was used. The antiradical activity was expressed as gallic acid equivalents (GAE) as reported in Table S4, where the antiradical activity is expressed as Inhibition % calculated as: Control Abs—Sample Abs/Control Abs × 100.

### 2.7. Evaluation of Antimicrobial Activity

The antimicrobial activity of each sample on the bacterial strains *Staphylococcus aureus* ATCC 29213, *Lactobacillus rhamnosus* GG ATCC 53,103, and *Pseudomonas aeruginosa* ATCC 27,853, each grown in its corresponding medium and conditions, was tested. The minimum inhibitory concentration (MIC) was evaluated using the broth microdilution method according to Clinical and Laboratory Standards Institute guidelines [22].

### 2.8. Cell Cultures

The human acute monocytic leukemia cell line (THP-1), purchased from the American Type Culture Collection (ATCC, Rockville, MD, USA), was cultured in RPMI (Roswell Park Memorial Institute) 1640 medium (Merck, Milan, Italy) supplemented with 10% fetal bovine serum (Merck, Milan, Italy), 2 mM *L*-glutamine, and 10 mM HEPES (4-(2-hydroxyethyl)-1-piperazineethanesulfonic acid) at 37 °C in a humidified 5% CO_2_ incubator. For differentiation, THP-1 cells were incubated in the presence of 20 ng/mL of phorbol myristate acetate (PMA) (Merck, Milan, Italy) for 72 h [23].

### 2.9. MTS Assay

Metabolic cell activity was performed by MTS assay in 96-well plates (Thermo Fisher Scientific, Waltham, MA, USA). Undifferentiated and differentiated cells were incubated with an activating stimulus (1 mM H_2_O_2_). After 8 h incubation, the cells were treated with garlic extracts (5, 10, 25, 50, 100 μg/mL). At the established time points (24 and 48 h), the incubation medium was harvested, and fresh complete RPMI containing 3-(4,5-dimethylthiazol-2-yl)-5-(3-carboxymethoxyphenyl)-2-(4-sulfophenyl)-2*H*-tetrazolium (MTS) at a concentration of 0.5 mg/mL was added to each well, both in untreated cells (assay control) and in treated ones. Cells were incubated for 4 h at 37 °C and 5% CO_2_. The absorbance was measured at 490 nm by means of a spectrophotometer (Multiscan GO Thermo Fisher Scientific, Waltham, MA, USA). The percentage of metabolically active cells was calculated by the equation (A/B) *100, where A = absorbance of treated samples and B = absorbance of the untreated control (set as 100%). All assays were performed in triplicate. The concentration and time incubation of H_2_O_2_ was chosen in accordance with preliminary results [23].

### 2.10. Analysis of Peroxynitrite Generation

Monocytes and macrophages (2 × 10^5^ cells/well) were grown and exposed to H_2_O_2_ and extracts in 6-well plates. At the established time points (24 and 48 h), the DAX-J2™ PON Green probe (Cell 205 Meter™ Fluorimetric Intracellular Peroxynitrite Assay Kit, AAT Bioquest, Pleasanton, CA, USA) was used for detecting the production of peroxynitrite by flow cytometry. Briefly, the exposure medium was removed, replaced with a fresh one containing 1 µL/mL of DAX-J2™ PON Green 500 ×, and incubated at 37 °C and 5% CO_2_ in the dark for 1 h. Then, cells were processed according to the manufacturer’s instructions. The analysis was performed by using a CytoFLEX Flow Cytometer (Beckman Coulter, Miami, FL, USA) equipped with a 488 nm laser with an FL1 (FITC) detector in a linear mode. The experiment was performed in triplicate. Relative fluorescence emissions of gated cells by means of their forward and side scatter properties (FSC/SSC) were analyzed with the CytExpert software (Beckman Coulter, Indianapolis, IN, USA), and they were expressed as mean fluorescence intensity (MFI) ratios on the unstained control [23].

### 2.11. Western Blot

Western blot analysis was performed as previously reported [24]. Monocytes and macrophage cells, after incubation of 24 and 48 h, were collected and lysed in radioimmunoprecipitation assay (RIPA) buffer. The proteins were quantified by a bicinchoninic acid assay. Total protein extracts were separated on a 10% sodium dodecyl sulfate–polyacrylamide gel and transferred to nitrocellulose membranes. Blots were probed and incubated with the following primary antibodies: anti-iNOS (NOS2 (N-20), sc-651), anti-nitrotyrosine (PNK, sc-55256), anti-COX-2 (ab52237), and p-NF-kB (p65) (sc-166748). The Super Signal Ultra chemiluminescence detection reagents (Pierce Biotechnology, Rockford, IL, USA) were used to detect the protein expression. A rabbit anti-human β-actin antibody (A5441; Sigma-Aldrich) was used as a control. A gel analysis software package (Gel Doc 1000; Bio-Rad) was employed to analyze the blot images. Results were expressed as mean values ± standard deviations (S.D.) of normalized densitometric values on the loading control.

### 2.12. Biological Data and Statistical Analysis

Each assay was replicated at least three times. Data are expressed as mean ± SD (standard deviation), and statistical significance was determined using the XLStat software (New York, NY, USA). A statistical approach was also carried out to the data obtained by ESI FT-ICR MS analyses by using the free software MetaboAnalyst (https://www.metaboanalyst.ca accessed on 8 July 2022) [25]. Raw data were aligned in a xy file within 5 ppm difference tolerance, relative intensities were normalized by sum, and the Pareto scaling method was applied to investigate the whole ‘Sulmona red garlic’ metabolome variation among the extracts of W, R, and B.

Statistical analyses for data obtained from monocytes/macrophages were performed by one-way analysis of variance (ANOVA) followed with Tukey’s multiple comparison test by means of the Prism 5.0 software (GraphPad, San Diego, CA, USA). All results were expressed as mean values ± standard deviations. Values of *p* ≤ 0.05 were considered statistically significant.

## 3. Results and Discussion

### 3.1. Hydroalcoholic Extraction

The adopted extraction parameters are crucial for the bioactive fraction isolation, being the last influenced by several factors such as time, extractant matrix weight and moisture, solvent choice, solvent/matrix ratio, and temperature. Several extraction techniques are reported in the literature using mainly solvents such as water, methanol, ethanol, and acetone. Previous published data confirmed that water is an efficient solvent for this extraction, being correlated with the highest extraction yields [15,26].

The main issues related to the use of aqueous extracts are the limited stability and the time-consuming step to obtain dry samples by conventional methods or by the expensive freeze-drying process. For these reasons the extraction protocol applied in the present work was based on the use of hydroalcoholic solution at room temperature, already reported by Recinella et al. [19] as an efficient extraction system. The hydroalcoholic extraction mixture represents a good compromise, both allowing to obtain relatively high extraction yields and solving, at the same time, the evaporation step problems. The application of such sustainable extraction method to garlic peels, a waste of the agronomic practices, would agree with the general principles of circular economy and green chemistry. The percentage yield of dry extract from plant material (R, W, B) were calculated and reported in Table S2.

As shown by the literature, the garlic bulb is mainly composed of water (65%), followed by carbohydrates (28%, mainly represented by fructans), sulfur compounds (1–4%), proteins (2%), fibers (1.5%), and free amino acids (1–1.5%), whereas cellulose, hemicellulose, and lignin are the main constituents of the peels [11]. The lowest extraction yields (2.2 and 3.3 g/100 g fresh weight, respectively, in 2020 and 2021 harvesting) were reported for W, a little lower than the values coming from R (3.9 and 5.2 g/100 g), as well as much higher yields were obtained by B (about 15 and 18 g/100 g) in agreement with the presence of co-extractable sugars. In each case, a higher extraction yield was afforded by the vintage in July 2021. Garlic white and red peels, as well as discarded small bulbs, could represent an interesting waste to recycle in co-product for the content, not only of antioxidant compounds but also of natural fibers. Nowadays, lignocellulosic fibers obtained from waste materials are attracting great interest in the production of bio-based products and for the production of useful biopolymeric materials [27].

### 3.2. Colorimetric Analysis

**Figure 1:**
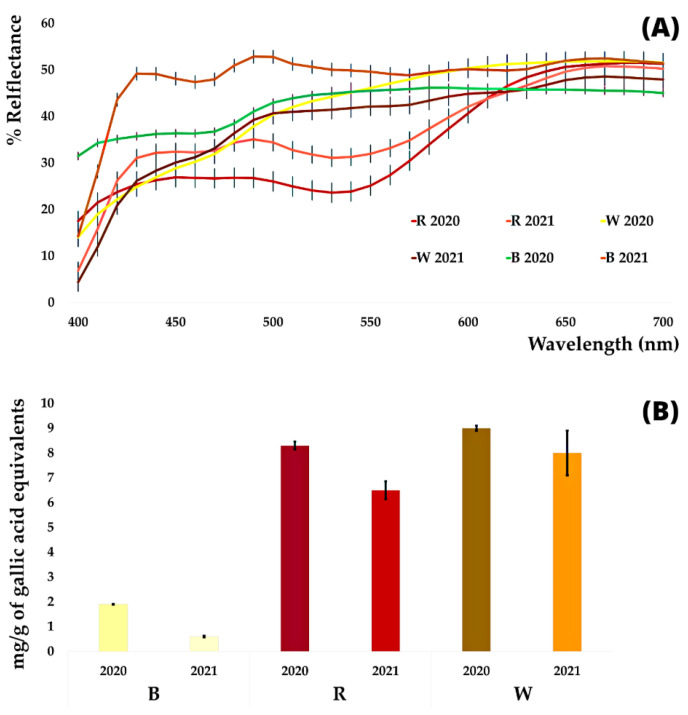

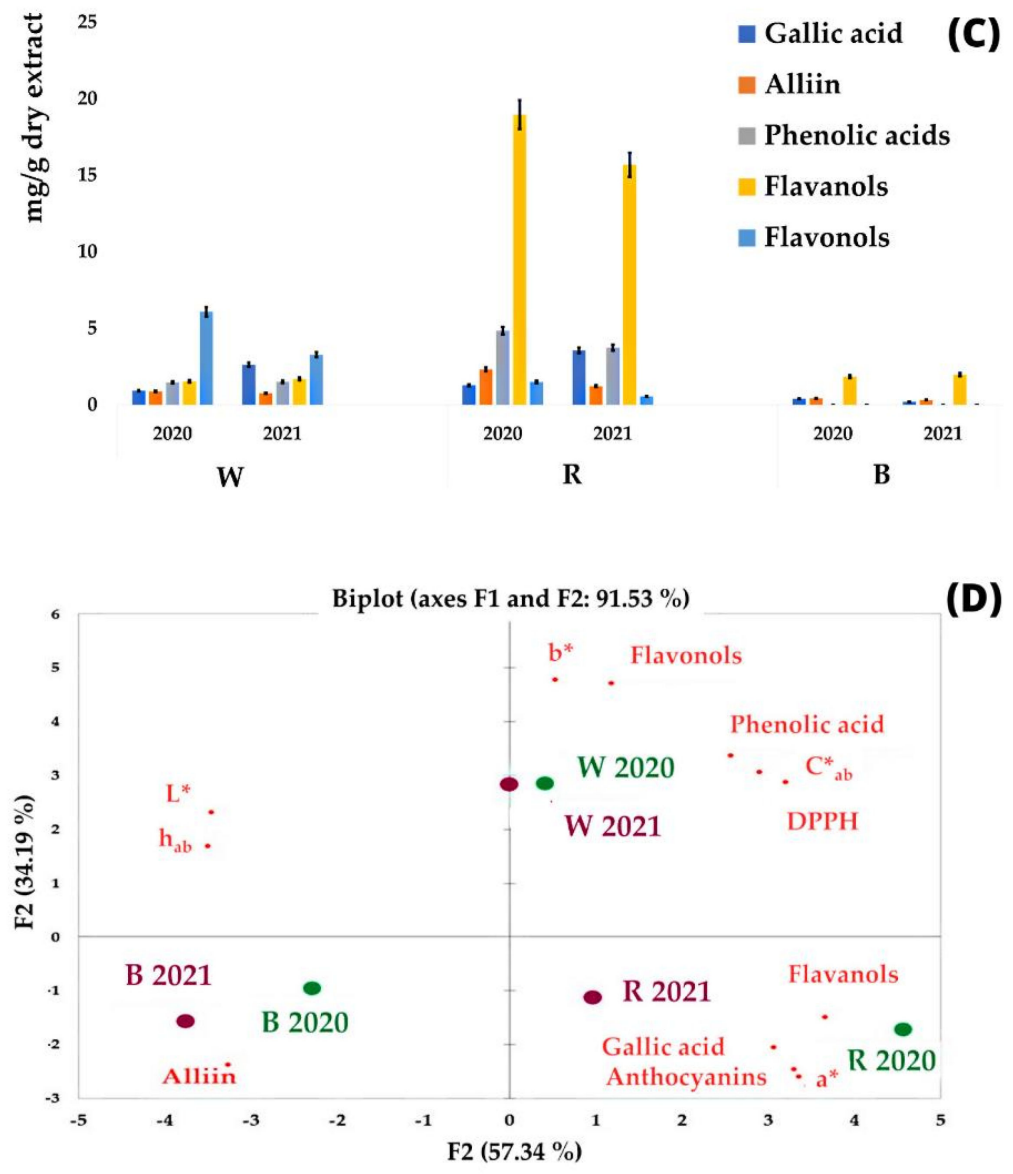
Reflectance curves (**A**), antiradical activity (**B**), bioactive compounds composition according to HPLC-DAD analyses (**C**), and PCA analysis of the obtained hydroalcoholic extracts (**D**). The reported deviation standard (SD) is <5%, *p*-value ≤ 0.05.

To evaluate the different garlic profiles, the three hydroalcoholic extracts were submitted to colorimetric analysis. The CIEL*a*b* data and the resulting reflectance profile curves are reported in Figure 1A (see also Table S3 and Figure S2).

The luminance L* ranges between 62 and 76, denoting clear and brilliant samples, only a little darker when a red component is present; a* values, near to 0 in B and W, rise to 17 in R coming from the harvest 2020, but it turns to a lower 8 (pale red or pink nuance) in the harvest 2021. The small residue presence of a yellow nuance in B (b* between 2 and 9) results concentrated in W, with b* values rising to 16–21. The highest values of a* were recorded for R, evidently correlated with the characteristic pigment responsible for the red coloration of the ‘Sulmona red garlic’. To our knowledge, only one study is available in the literature in which, on the basis of MS analysis, an organosulphur structure was proposed to provide this color [28]. On the contrary, on the basis of high-performance liquid chromatography-mass spectrometry (HPLC-MS), some authors attributed the red color of selected Australian garlic cultivars to specific anthocyanins [29]. Moreover, anthocyanins were also shown in some garlic samples by MS analysis in our previous work [16].

The very pale yellow exhibited by B and concentrated in W is probably associated with pigments such as flavonoids [30]. A marked difference is shown between reflectance curves of R, by the two different harvesting date, between 600 and 650 nm, associated with the significant decrease in a* value in the harvesting 2021. Comparing these data with other previously published on hydroalcoholic extracts of garlic powder [19], values reported on hydroalcoholic garlic powders (L* = 60, a* = −0.8, and b* = 7) only partially agree with those found for W, showing little higher b* values. Finally, in the work carried out on different garlic cultivars, the experimental mean values (L* about 44, a* about 7, and b* about 24) seemed to show a higher pigments content, not only denoted by the higher values of a*, but also confirmed by the lower luminance expressed by the sample [16].

### 3.3. DPPH Analysis

There is evidence in the literature that phenolic and organosulphur compounds, present in the phytocomplex of garlic, and highly bioavailable in humans, have a beneficial effect on health, as they are able to counteract the harmful action of free radicals [31]. The DPPH in vitro assays (Figure 1B; Table S4), highlight similar trend of activities in sample from different years confirming for B the lowest antiradical inhibition activity (2.2–3.2%), while the highest inhibition was recorded for W 2021 (about 39%). Interesting activity (17–20%) was also expressed by R. These antiradical activity results well correlated with the polyphenols content shown by the HPLC and MS analyses described in the next paragraphs, but our findings showed only partial correlation with literature data, where inhibition range 20–45% is related to garlic cloves or bulb from different cultivars [24,32,33].

### 3.4. HPLC-DAD Analysis

Chromatograms of the dried hydroalcoholic extracts of R, W, and B were recorded at 280 nm for the identification of benzoic and hydroxycinnamic acids, flavanols, and organosulphur compounds, at 360 nm for the identification of flavonols, and at 520 nm for the identification of the red pigments.

According to the literature, extracts were exceptionally rich in different bioactive compounds and some different molecules or classes were identified in the chromatograms [32,34,35]. Chromatograms are reported in Figure S3. R and W showed a rich phytocomplex characterized by gallic acid, alliin, catechin, epicatechin, caffeoyl quinic derivatives, caffeic, *p*-coumaric, ferulic and sinapic acids, myricetin, quercetin-3-galactoside, quercetin, kaempferol, and a kaempferol-monoglycosyl derivative. In contrast, a simpler profile was shown by B, consisting mainly of gallic acid, alliin, and catechin. As reported in Table S5, bioactive molecules were more represented in tunicae, except alliin and catechins. Epicatechin, the most represented compound in tunicae, with higher values in R, was in both cases more expressed in harvest 2020.

By the comparison (Figure 1C) of alliin content and the mainly represented bioactive polyphenols in the three separate garlic parts, R showed the richest phytocomplex with a higher phenolic acids content and a significant presence of epicatechin. On the contrary, flavonols are more represented in W. Harvest 2020 and are generally richer in bioactive compounds, with the only exception represented by gallic acid. This evidence reflects the above-reported antiradical activity. The data are hardly comparable with the literature because those are mainly related to the garlic clove or garlic powder, but not to the separated parts [19,31,36].

### 3.5. Principal Component Analysis (PCA)

The PCA was carried out on data related to CIEL*a*b* parameters, HPLC-DAD and DPPH analysis in order to find the best correlation. The values have been scaled using XLSTAT 2021 software with the unit variance scale. As shown in Figure 1D, the *x*-axis represents the first PCA dimension (F1, 57.34% of total variance), whereas the *y*-axis is the second PCA dimension (F2, 34.19% of total variance). Extracts related to W of both harvest dates correlate to the antiradical activity with the presence of the highest flavonols and phenolic acids content, the highest color saturation (Chroma, C*ab), and positive b* values. Extracts related to R correlate to a slightly less evident antiradical activity with a richer presence of flavanols. Gallic acid and anthocyanins, more represented in R, show a lower correlation with the antiradical activity. B extracts, associated with the highest content of alliin, correlate with higher values of lightness and tonality (h_ab_) due to the lower pigment and bioactive contents.

### 3.6. ESI FT-ICR Assignment

The ESI FT-ICR mass spectra of hydroalcoholic extracts showed peaks due to protonated compounds or alkali metal adducts for positive polarity mode and deprotonated species or chloride adducts for negative polarity mode. This method has been already exploited to the (moderately) polar fraction of several complex matrices, such as essential oils and foodstuffs, guaranteeing a broad and reliable untargeted chemical profile [37,38]. However, an accurate metabolite quantification in direct infusion ESI FT-ICR MS is hampered due to possible ion-suppression effects and signal response disparities.

The major metabolites in hydroalcoholic extracts of R2020, W2020, B2020, R2021, W2021, and B2021 revealed by ESI FT-ICR MS are listed in Table 1. Table S6 (ESI (+)) and Table S7 (ESI (−)), available as Supplementary Materials, show comprehensive lists of assigned elemental formulas, together with the putative compound annotations, the theoretical *m*/*z* ratios, the mass deviation, and the intensity, investigated in either ionization mode.

For each extract, more than 400 formulas have been identified in both polarity modes (Table 2). On the whole, a larger number of hits has been identified in positive ionization mode. While B displays a similar number of assigned compounds in both harvest years, R presents relatively richer diversity in 2020 than in 2021, in the opposite trend with respect to W. Some higher intensity peaks have been assayed by CID experiments confirming molecular assignments, like in the case of anethole [M+H]^+^ *m*/*z* 149 (C_10_H_12_O) and ferulic acid [M+H]^+^ *m*/*z* 195 (C_10_H_10_O_4_) (Figure S4).

**Figure 2:**
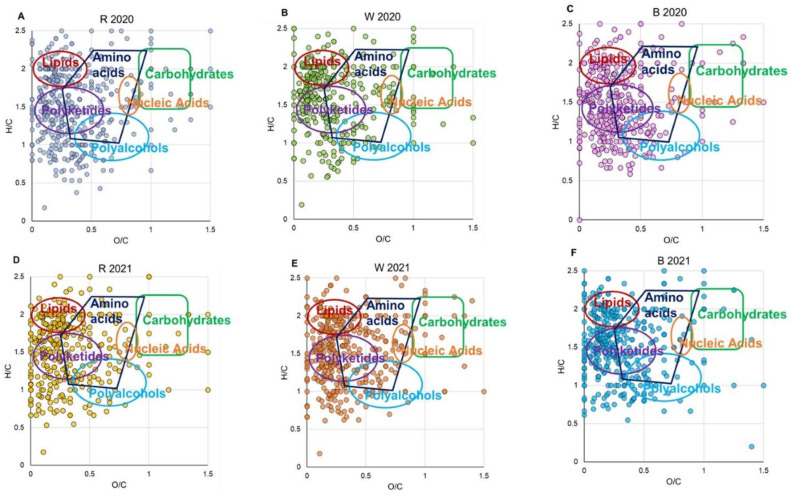
Van Krevelen diagrams (elemental plot) obtained from the molecular formulas achieved by ESI FT-ICR MS analyses of hydroalcoholic extracts of R 2020 (**A**), W 2020 (**B**), B2020 (**C**), R2021 (**D**), W2021 (**E**), and B2021 (**F**). Each point on the graph represents a distinctive molecular formula.

**Figure 3:**
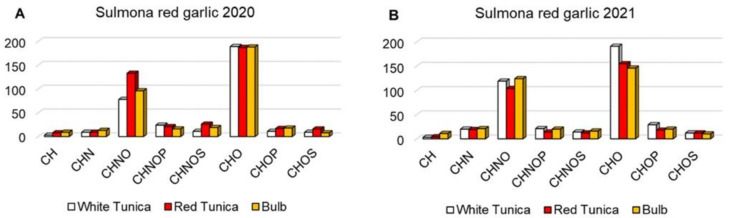
Histograms of relative frequency of CH, CHN, CHNO, CHNOP, CHNOS, CHO, CHOP, and CHOS comparing W, R, B 2020 (**A**) and W, R, B 2021 (**B**).

An overview of the assigned molecular formulas is assisted by van Krevelen diagrams (vKd), known as elemental ratio analysis, and relative frequency histograms. VKd affords a qualitative visualization of densities of molecular classes based on their individual H/C versus O/C ratios, as shown in Figure 2. R2020 shows a relatively higher density of molecular formulas in the areas of lipids and polyketides, followed by amino acids and (relatively much less) polyalcohols, carbohydrates, and nucleic acids (Figure 2A). W2020 displays a larger number of hits in the region of lipids but fewer entries for polyketides, amino acids, and polyalcohols (Figure 2B). W2021 (Figure 2E) shows the most abundant entries for amino acids, while B2020 and B2021 (Figure 2C and F) clearly represent a large population of metabolites in the regions of lipids and polyketides. VKd may be useful to elucidate metabolic pathways among entries thanks to trend lines with characteristic slopes or intercepts that may be envisaged. Figure S5 shows lines associated with paths, including hydrogenation/dehydrogenation (lines A), oxidation/reduction (lines B), hydration/dehydration (lines C), and methylation/demethylation or alkyl chain elongation (lines D). As shown in Figure 3, the averaged relative frequency distribution histograms of CH, CHN, CHO, CHNO, CHOP, CHOS, CHNOP, and CHNOS elemental compositions reveal that all extracts present the major biodiversity of CHO species (more hits in W 2021), with organic acids, polyphenols, lipids, and sugars, followed by CHNO compounds (more hits in R 2020), mostly represented by amino acids and alkaloids and, in smaller amount by CHNOS, with metabolites responsible for characteristic garlic flavor and bioactivity, CHOP, CHNOP, and CHOS, while CH hits are barely represented. Some metabolites have been recorded in all samples, like sebacic, azelaic, and myristic acids, α-tocopheronolactone, arginine, and apiole, known for their antioxidant activity and antitumor effects [39]. Other phytochemicals were also widely identified, such as ferulic acid, citronellic acid, fraxetin, palmitic acid, cysteic acid, asparagine, allantoic acid, pimelic acid, ribose, glucose, purine, and pyridine. Hereafter, results will be presented according to the compound classes.

#### 3.6.1. Amino Acids and Derivatives

ESI FT-ICR MS revealed several essential amino acids, including arginine, leucine, lysine, valine, glycine, and taurine, with tryptophan observed only in red tunica. Non-essential amino acids asparagine, alanine, and citrulline have also been found. Amino acids derivatives, such as proline-betaine, and small peptides, such as *L*-prolyl-*L*-proline and glycylproline (in R and B, 2021), *L*-isoleucyl-*L*-proline (in W 2020), aspartyl-proline (in W and B, 2021), *N*-acetyl-leucine (B), and cysteinylglycine (R) were identified. Glutathione and derivatives, including *S*-(formylmethyl)glutathione (in R) and *S*-(hydroxymethyl)glutathione (in W), were also recovered. According to a previous report, allysine, a key component in garlic, was found in B [16].

An interesting case regards the assignment of a peak detected at *m*/*z* 178.05310 in red tunica and, to a smaller extent, bulb samples (Figure S6), corresponding to the molecular formula C_6_H_11_NO_3_S and compatible with three different protonated species: alliin, *S*-(1-propenyl)-*L*-cysteine sulfoxide (1-PeCSO) and *N*-formylmethionine. Comparison between the CID mass spectrum and Metlin database displays the occurrence of fragments (*m*/*z* 150, 160 in red) belonging to *N*-formylmethionine and a fragment (*m*/*z* 88 in blue) fitting to [C_3_H_5_NO_2_+H]^+^, present in both alliin and 1-PeCSO spectra (Figure S7). All species may thus contribute to *m*/*z* 178, with the compatible presence of 1-PeCSO as a potential key compound associated with red tunica pigmentation [28].

#### 3.6.2. Organosulphur Compounds

ESI FT-ICR MS analyses of R have identified several OSCs, including alliin and allicin, *S*-allyl-*L*-cysteine, and homocysteinesulfinic acid. Other OSCs have also been found, comprising dipropyl disulfide (R 2020 and B 2020, 2021), allylsulfide (R 2021 and W 2020), and diethyl sulfide (W 2020 and B 2021). Interestingly, cysteic acid was detected in all tunica extracts except for W 2020.

#### 3.6.3. Organic Acids

Several peaks have been attributed to organic acids, such as phenolic acids. *p*-Coumaric acid (all samples), ferulic, ascorbic acid, and malic acid were widely spread. Moreover, W showed the presence of glutaric acid and feruloyl-glucose. Caffeic and dihydrocaffeic acids were identified in almost all samples, while R showed the presence of citric, quinic, hydroxy-, and glutamic acids. Interestingly, dicarboxylic acids, including azelaic, allantoic, and pimelic acids, traditionally used as ingredients in functional foods and as skin protectants, were discovered in all samples (despite allantoic and pimelic acids being absent in B 2020 and R 2021) [40].

#### 3.6.4. Fatty Acids

The CHO molecular formulas classified as free fatty acids (FA), mainly obtained in the negative ionization mode, present compounds in the group of saturated, i.e., stearic, palmitic, (methyl)-myristic, and unsaturated (linderic, oleic, palmitoleic) acids spread in all samples. Conversely, myristoleic acid was detected exclusively in samples harvested in 2020. In addition, ω-FA (omega-fatty acids), such as ω-3 linolenic and ω-6 linoleic acids, have been observed in W 2020 and B 2021, whereas R was rich in ω-3 eicosapentaenoic acid.

#### 3.6.5. Miscellaneous

Several other nutrients have been detected by ESI FT-ICR analysis, including polyphenols and their derivatives, known for their strong antioxidant and antimicrobial activities [24]. Sulfated flavonoids (e.g., quercetin 3-(3″-sulphatoglucoside) and quercetin 3-rhamnoside-3’-sulfate) have been revealed in W 2020, and quercitrin in R 2020 [41], as well as flavones and flavanones (e.g., apigenin 5,7-dimethyl ether 4’-galactoside, naringenin 7-*O*-(2″,6″-di-*O*-α-rhamnopyranosyl)-β-glucopyranoside, and apigeniflavan) have been detected in R 2021 and B 2020, and apigenin 7-methyl ether 5-(6″-malonylglucoside), turned out in W 2021. Noteworthy, purpurogallin, a red-orange compound, and rosinidin, a cyanidin-derived pigment, were found exclusively in R 2020. In addition, red tunica stood out for its content of chalcone, dihydrochalcone, and flavonoids, all metabolites with countless biological activities [42]. All samples contain terpenoids such as apiole, whereas gingerol has been observed only in 2021 extracts. Among vitamins, retinoic acids and vitamin D3 derivatives are widely spread. δ-tocopherol has been found exclusively in R samples, β-tocopherol in B 2021, and β-carotene in W 2021. Moreover, caffeoyl alcohol, a relevant lignol precursor, has been identified in W 2021 and B 2020, whereas fraxetin in most samples, bergapten in extracts 2021, and umbelliferone in R.

#### 3.6.6. Statistical Analysis: Principal Component Analysis

The application of PCA as a statistical method for data visualization has allowed us to gain an explorative data analysis by clustering samples based on relevant similarities/differences. Figure S8A displays the PCA scores obtained from MS data acquired in negative ionization mode, which seemed more interesting. The first two components point out significant discrimination among samples close to 70%. Whereas the first component enables us to discern among the harvest year 2020 samples clustering on the left and 2021 on the right, the second one differentiates W from R. Notably, B is closer to R than to W datasets. Moreover, dendrograms (Figure S8B) confirm a correlation between samples harvested in the same year. Metabolites presenting *p*-value < 0.05 were considered statistically relevant. Among them, in negative ionization, palmitic ([C_16_H_32_O_2_-H]^−^), stearic ([C_18_H_36_O_2_-H]^−^), and phosphatidic acids (22:4/0:0) ([C_25_H_43_O_7_P-H]^−^) stood out, as shown in Figure S9.

### 3.7. Antibacterial Activity

All the garlic extracts showed no antibacterial activity against the three strains tested (*S. aureus*, *P. aeruginosa*, and *L. rhamnosus*) with MIC values higher than 800 μg/mL for both the harvesting years (data not shown). The antibacterial activity of garlic is confirmed by different studies [29,30]; however, the extraction procedure, based on concentrating particular compounds, might contribute to determining the properties of the extract and so its efficacy against different bacterial strains. Anyhow, the data related to *L. rhamnosus* species evidenced no toxicity against one of the most representative probiotics in the human gut.

### 3.8. Biological Evaluation

#### 3.8.1. Effect of Garlic Extracts on Cell Viability

The effect on cell survival of the single garlic extracts on undifferentiated (monocyte) and differentiated (macrophages) THP-1 cells were assessed through dose-response experiments (concentrations of 5, 10, 25, 50, and 100 μg/mL) using the MTS assay after 24 and 48 h of treatment. The viability of THP-1 cells did not change within all the ranging concentrations examined, suggesting high biocompatibility (data not shown). To evaluate the potential cytoprotective effect of garlic extracts toward oxidative stress, we used an H_2_O_2_-induced cytotoxicity cell model (H_2_O_2_ concentration = 1 mM) and an incubation time of 8 h, identified with preliminary experiments by MTS assay (Figure S10A–D). As demonstrated in Figure S10A,C, the metabolic activity showed that monocytes incubated with H_2_O_2_ at 24 and 48 h, respectively, significantly decreased their viability (about 20%) with respect to the untreated group (100%). The extracts exerted no change in cellular metabolic activity against the oxidant effect of H_2_O_2_. In particular, the stimulation with H_2_O_2_ reduced the cell metabolic activity compared to controls. All the garlic extracts tested did not or weakly result in a significant recovery as regards the metabolic activity of monocytes compared to H_2_O_2_ alone. The most effective extract is represented by the red tunica one, able to restore the metabolic activity to values similar to untreated cells already at a concentration of 10 μg/mL after 24 h of incubation (125%) and even more at 100 μg/mL. On the contrary, despite the more pronounced pro-oxidant effect in macrophages, garlic extracts showed a strong ability to counteract this cytotoxic stimulus and thus protect cells as regards their metabolic activity. In conclusion, a significant decrease in viability was observed in H_2_O_2_-stimulated THP-1 macrophage-like cells both at 24 and 48 h with respect to untreated samples. Cells exposed to the three different garlic extracts at all the concentrations (5–100 μg/mL) showed increased viability in a significant manner if compared to H_2_O_2_-stimulated cells (Figure S10B,D).

The biological evaluation was carried out on both harvest dates (2020–2021). Since there were no statistically significant differences (data not shown), the subsequent evaluations were carried out with the extracts referring to the 2020 collection.

#### 3.8.2. Garlic Extracts Modulate iNOS and COX-2 Protein Expression in H_2_O_2_-Stimulated THP-1 Cells

As widely known, in response to chemical or physical stimuli, activated monocyte/macrophage cells are crucial in the inflammatory response, mediating the secretion of various cytokines/chemokines, oxygen reactive species (ROS), nitrogen reactive species (RNS), and growth factors [43]. Based on viability and cytotoxicity data, the concentrations of 10 and 50 μg/mL were used in further experiments to investigate whether the W, R, and B extracts could own an anti-inflammatory activity on immunocompetent cells.

**Figure 4:**
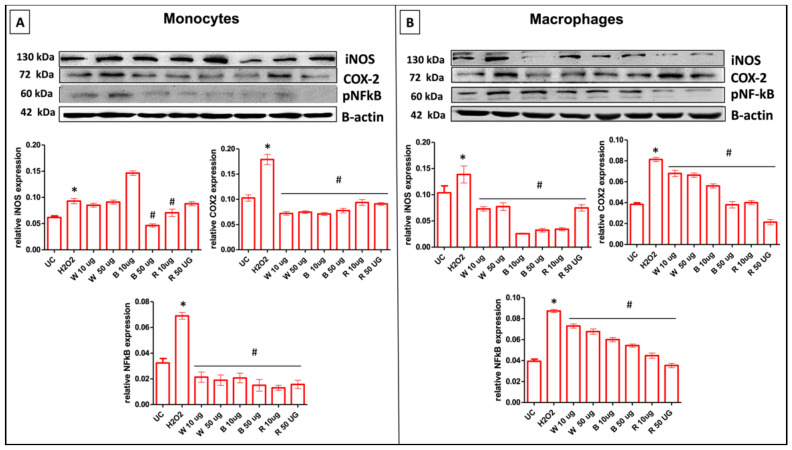
iNOS, COX-2, and NF-ĸB expression levels in undifferentiated (monocytes) and differentiated (macrophages) cells. Effect of different concentrations (10 and 50 μg/mL) of garlic extracts, at 24 h of incubation, on the expression of iNOS, COX-2, and p-NF-ĸB protein in H_2_O_2_-stimulated cells. Representative image of Western blot experiments on undifferentiated (monocytes) cells (**A**) and differentiated cells (macrophages) (**B**) (top). At the bottom, averaged band density of iNOS, COX-2, and p-NF-ĸB normalized vs. β-actin. The data represented means ± SD (*n* = 3). * *p* < 0.05 vs. UC; ^#^ *p* < 0.05 vs. H_2_O_2_-treated cells. UC: untreated control, W: white tunica, B: bulb, R: red tunica.

Therefore, we investigated the effect of the three extracts on the protein expression of the well-known proinflammatory mediators iNOS and COX-2 after H_2_O_2_ stimulation for 24 h in undifferentiated and differentiated THP-1 cells (Figure 4). As expected, the H_2_O_2_ stimulation upregulates iNOS protein levels in both the cell type (0.09 for monocytes and 0.14 for macrophages) with respect to untreated controls (0.06 and 0.10, respectively). In monocytes, the exposure to extracts following the H_2_O_2_ stimulation led to a significant anti-inflammatory response, with differences among them. The best scenario for the decrease in the iNOS protein expression resulted in the presence of B extracts at the highest concentration (50 μg/mL) (0.04), while R showed a significant reduction only at the lowest concentration (10 μg/mL) (0.07) (Figure 4A). In parallel, the anti-inflammatory effect was more pronounced for macrophages. All the garlic extracts tested here exerted a distinct inhibitory effect on the iNOS protein compared to H_2_O_2_ alone. The densitometric analysis revealed a more drastic reduction in the presence of B, at both doses, along with in the presence of R at the lowest concentration (10 µg/mL) (Figure 4B).

As for COX-2 protein expression, the H_2_O_2_ treatment caused a similar effect in both the two cell phenotypes, with significant induction of this protein if compared to untreated cells. All the garlic extracts tested in H_2_O_2_-stimulated monocytes displayed a significant reduction in the expression of COX-2 (Figure 4A). As regards macrophages, the COX-2 down-regulation was more significant for all extracts. Notably, a drastic reduction in the protein expression of COX-2 in the presence of R at both doses can be highlighted (Figure 4B). It has been broadly reported that the transcription factor NF-κB is one of the master regulators of proinflammatory processes, driven by genes such as iNOS and COX-2. In this light, we, therefore, investigated the effect of garlic extracts in H_2_O_2_-stimulated monocytes/macrophages in terms of NF-ĸB modulation at a protein level by immunoblot analysis. Our results showed that the inhibition of NF-ĸB in H_2_O_2_-stimulated monocytes/macrophages exposed to garlic extracts, mainly in the presence of R at both concentrations, provides a plausible explanation for the suppression of the proinflammatory proteins iNOS and COX-2 (Figure 4A,B).

#### 3.8.3. Garlic Extracts Attenuate Nitrosative Stress in H_2_O_2_-Stimulated THP-1 Cells

The upregulation of iNOS expression is correlated with the degree of inflammation as well as the presence of NO. Although NO can be directly harmful to target cells, in inflammatory conditions, large amounts of NO and superoxide are formed, and their combination leads to the formation of reactive nitrogen species, such as the peroxynitrite (ONOO^−^) [44].

**Figure 5:**
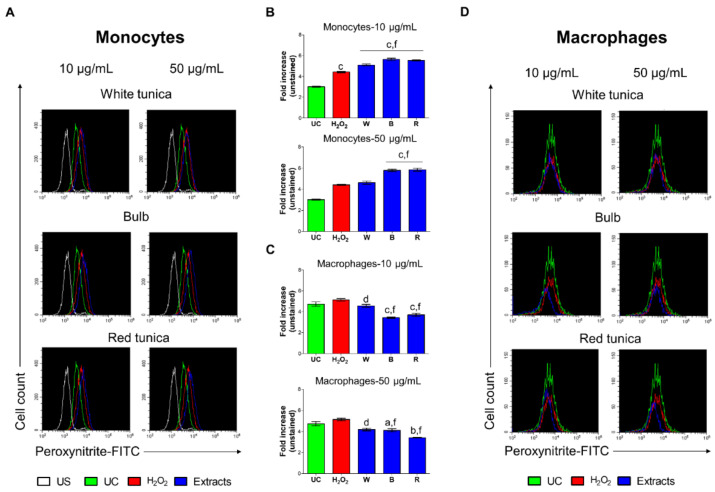
Generation of peroxynitrite by monocytes and macrophages after 48 h of exposure. (**A**,**D**) Histograms were obtained by flow cytometry and are generated by plotting the cell count (*y*-axis) and the FITC fluorescence emission (*x*-axis). (**B**,**C**) Bar graphs show the fold increase in the mean fluorescence intensities (MFIs) related to the emissions in the FL-1/FITC channel, which are proportional to the generation of peroxynitrite. Values are the ratios of the MFI generated from each sample on the unstained control (negative). a = *p* < 0.01, b = *p* < 0.001 and c = *p* < 0.0001 between treated samples and UC; d = *p* < 0.01, e = *p* < 0.001, and f = *p* < 0.0001 between samples and H_2_O_2_. UC = untreated control; US = unstained sample.

In this study, we detected intracellular ONOO^−^ by fluorescence-based quantitative measurements. In monocytes, peroxynitrite levels were significantly enhanced after the H_2_O_2_ stimulation at both times of exposure (MFI = 4.1 at 24 h and MFI = 7.9 at 48 h) compared to untreated cells (MFIs = 2.5 and 4.3, respectively). After 24 h of treatment with garlic extracts (Figure S11), W exerted no effect, whereas B and R increased peroxynitrite levels in H_2_O_2_-treated monocyte cells at both concentrations (Figure S11A,B). On the contrary, a longer treatment time (48 h) caused a reduction in peroxynitrite in the same cells, although the reduction was significant only when extracts were used at 50 μg/mL (Figure 5A,B). In PMA-differentiated cells, the effect of the oxidant was not much pronounced (24 h) (Figure S11B,C) or resulted even reduced (48 h) (Figure 5B,C) regarding the peroxynitrite production compared to untreated cells. Garlic extracts showed cytoprotection related to H_2_O_2_-induced peroxynitrite production, an effect that became statistically significant when R was administered for 24 h at both concentrations.

**Figure 6:**
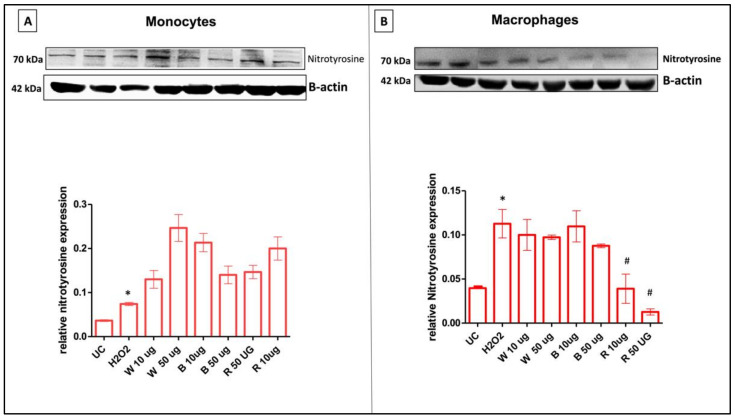
Nitrotyrosine protein expression in undifferentiated (monocytes) and differentiated (macrophages) cells. Effect of different doses (10 and 50 μg/mL) of garlic extracts after 24 h of incubation on the expression of 3-nitrotyrosine protein in H_2_O_2_ stimulated cells. Representative image of Western blot experiment on undifferentiated (monocytes) cells (**A**) and differentiated cells (macrophages) (**B**) (top). At the bottom, averaged band density of protein normalized vs. β-actin. The data represented means ± SD (*n* = 3). * *p* < 0.05 vs. UC; ^#^ *p* < 0.05 vs. H_2_O_2_-treated cells.

Lastly, the nitration of tyrosine residues as 3-nitrotyrosine (3-NT), generally considered a biomarker of peroxynitrite production, was evaluated. Post-translationally modified nitrated proteins were shown to accumulate in both monocytes and macrophages after H_2_O_2_ stimulation for 24 h. At the same experimental time, when cells are exposed to the three garlic extracts, differences in the responsiveness between undifferentiated and differentiated cells can be highlighted, confirming the trend observed for peroxynitrite. Indeed, in monocytes, the presence of garlic extracts resulted in a significant increase in tyrosine nitration at 24 h when compared to control cells (Figure 6A). Conversely, in PMA-differentiated cells, garlic extracts were able to restore values of tyrosine nitration similar to the control cells. Moreover, R was observed to behave as the most promising antioxidant and anti-inflammatory extract among those investigated (Figure 6B).

## 4. Conclusions

A sustainable extraction method was developed and applied to garlic peels and small peeled garlic bulbs, which represent a waste of agronomic practices. Extracts obtained by red and white tunicae exerted a significant antiradical activity, and PCA analysis of color parameters, polyphenol contents, and DPPH assays showed significant differences among the different obtained samples, also confirmed by the metabolite profile obtained by direct infusion high-resolution mass spectrometry (ESI FT-ICR MS). MS analyses revealed more than 400 molecular formulas and highlighted the great abundance of lipids, polyketides, amino acids, and organosulfur compounds. Statistical analysis displayed a clear separation between two different harvest years and the closeness between R and B samples.

Garlic extracts were here tested, under oxidative stress conditions, on immunocompetent cells to demonstrate cytoprotection in terms of viability and expression of proinflammatory proteins. Our data highlight that all the garlic extracts counteract nitrosative stress in H_2_O_2_-stimulated monocytes/macrophages, downregulating expression levels of inflammation-related proteins (COX-2 and iNOS) via the modulation of the transcription factor NF-ĸB. In particular, the red tunica extract emerged as the most promising one in terms of nitrosative stress counteraction.

The multimethodological approach afforded a detailed fingerprint of the considered wastes allowing us to hypothesize their reuse in different applications. The presence of amino acids, polyphenols, organic acids, organosulphur compounds, lipids, and so on suggests potential employment in the cosmetic, pharmaceutic, and food supplements industries [45,46].

## Figures and Tables

**Table 1:** Major metabolites in hydroalcoholic extracts of red and white tunicae and bulb of ‘Sulmona red garlic’ collected in 2020 and 2021 and revealed by ESI FT-ICR MS.

*ESI (+) FT-ICR MS*									
Putative Annotation	Adduct	Formula (M)	Theoretical m/z ^a^	ppm ^b^ R2020	ppm ^b^ W2020	ppm ^b^ B2020	ppm ^b^ R2021	ppm ^b^ W2021	ppm ^b^ B2021
*Carbohydrates*									
Hexose	[M+Na]^+^	C_6_H_12_O_6_	203.05261	−1.4	0.2	-	-	0.0	-
	[M+K]^+^	C_6_H_12_O_6_	219.02655	4.3	−0.4	-	-	−0.6	-
Mannitol	[M+Na]^+^	C_6_H_14_O_6_	205.06814	-	0.6	-	-	1.5	-
Lactose	[M+Na]^+^	C_12_H_22_O_11_	365.10543	−1.3	-	-	-	2.0	-
*Amino acids*									
Glycine	[M+Na]^+^	C_2_H_5_NO_2_	98.02139	-	−1.4	-	−2.9	-	-
Leucine/ Isoleucine	[M+H]^+^	C_6_H_13_NO_2_	132.10191	1.0	−1.3	-	-	-	-
	[M+Na]^+^	C_6_H_13_NO_2_	154.08385	−4.6	3.4	-	-	-	-
Valine	[M+H]^+^	C_5_H_11_NO_2_	118.08626	2.2	2.4	-	-	-	-
Asparagine	[M+K]^+^	C_4_H_8_N_2_O_3_	171.01665	−2.9	-	-	4.3	0.7	-
Phenylalanine	[M+H]^+^	C_9_H_11_NO_2_	166.08626	1.1	-	−2.7	−1.4	-	-
Lysine	[M+H]^+^	C_6_H_14_N_2_O_2_	147.11280	0.2	−0.5	3.8	-	-	-
Arginine	[M+H]^+^	C_6_H_14_N_4_O_2_	175.11895	0.3	0.1	−0.2	0.0	-	−0.2
	[M+K]^+^	C_6_H_14_N_4_O_2_	213.07484	0.4	-	-	-	2.1	−4.3
Allysine	[M+H]^+^	C_6_H_11_NO_3_	146.08079	-	-	-	-	-	2.6
	[M+Na]^+^	C_6_H_11_NO_3_	168.06337	-	-	−1.5	-	-	-
*Organic acids*									
Cysteic acid	[M+H]^+^	C_3_H_7_NO_5_S	170.01141	4.3	-	-	2.1	−4.1	-
Coumaric acid	[M+H]^+^	C_9_H_8_O_3_	165.05462	−1.4	3.2	4.6	1.1	−4.2	3.1
Ferulic acid	[M+H]^+^	C_10_H_10_O_4_	195.06519	4.3	3.6	3.9	-	3.5	-
Azelaic acid	[M+Na]^+^	C_9_H_16_O_4_	211.09408	−1.6	−0.3		−1.2	−0.8	−1.3
Ascorbic acid	[M+Na]^+^	C_6_H_8_O_6_	199.02174	-	-	-	−2.2	-	−2.9
*Fatty acids*									
Decenedioic acid	[M+Na]^+^	C_10_H_16_O_4_	223.09408	−2.8	-	-	-	−0.9	−2.4
Palmitic acid	[M+Na]^+^	C_16_H_32_O_2_	279.22945	−0.3	-	-	-	-	-
Palmitoleic acid	[M+Na]^+^	C_16_H_30_O_2_	277.21380	1.7	0.1	-	-	-	-
Oleic acid	[M+H]^+^	C_18_H_34_O_2_	283.26316	2.4	-	-	-	-	-
Myristic acid	[M+Na]^+^	C_14_H_28_O_2_	251.19807	-	0.3	-	-	-	-
	[M+K]^+^	C_14_H_28_O_2_	267.17237	-	-	-	−1.1	-	3.4
Myristoleic acid	[M+Na]^+^	C_14_H_26_O_2_	249.18250	−0.8	−1.2	-	-	-	-
*Other compounds*									
Purpurogalin	[M+H]^+^	C_11_H_8_O_5_	221.04445	2.0	-	-	-	-	-
Rosinidin	[M+H]^+^	C_17_H_15_O_6_	316.09414	2.9	-	-	-	-	-
Apiole	[M+H]^+^	C_12_H_14_O_4_	223.09572	-	3.4	3.4	2.0	-	-
	[M+Na]^+^	C_12_H_14_O_4_	245.07843	3.4	−2.0	-	-	0.1	−2.1
Gingerol	[M+Na]^+^	C_21_H_34_O_4_	373.23484	-	-	-	0.2	−1.9	-
***ESI (−) FT-ICR MS***									
**Putative** **Annotation**	**Adduct**	**Formula (M)**	**Theoretical m/z**	**ppm R2020**	**ppm W2020**	**ppm B2020**	**ppm R2021**	**ppm W2021**	**ppm B2021**
*Carbohydrates*									
Hexose	[M-H]^−^	C_6_H_12_O_6_	179.05599	4.3	−0.4	-	-	−0.6	-
*Amino acids*									
Asparagine	[M-H]^−^	C_4_H_8_N_2_O_3_	131.04622	-	2.4	-	-	-	-
*Fatty acid*									
Octenoic acid	[M-H]^−^	C_8_H_14_O_2_	141.09210	3.0	-	-	-	-	-
Palmitic acid	[M-H]^−^	C_16_H_32_O_2_	255.23295	−0.4	−0.2	−1.1	2.8	-	1.0
Palmitoleic acid	[M-H]^−^	C_16_H_30_O_2_	253.21719	-	-	-	-	0.5	-
Oleic acid	[M-H]^−^	C_18_H_34_O_2_	281.24860	1.1	-	1.2	−0.3	−0.1	−2.0
Myristic acid	[M-H]^−^	C_14_H_28_O_2_	227.20176	-	-	-	-	−0.5	-
	[M+Cl]^−^	C_14_H_28_O_2_	263.17833	2.1	0.4	-	−2.1	1.9	-
Stearic acid	[M-H]^−^	C_18_H_36_O_2_	283.26425	−1.2	−0.4	−0.5	0.9	-	-
Lauric acid	[M-H]^−^	C_12_H_24_O_2_	199.17035	2.4	-	−0.7	-	−2.4	-
*Organic acids*									
Quinic acid	[M-H]^−^	C_7_H_12_O_6_	191.05611	3.3	-	-	-	-	-
Malonic acid	[M+Cl]^−^	C_3_H_4_O_4_	138.98036	4.5	-	-	-	-	-
Oxalacetic Acid	[M-H]^−^	C_4_H_4_O_5_	130.99856	-	-	-	-	-	0.3
Ferulic acid	[M+Cl]^−^	C_10_H_10_O_4_	229.02731	-	2.1	-	-	-	-
Malic acid	[M-H]^−^	C_4_H_6_O_5_	133.01389	-	-	-	2.7	-	-
*Other compounds*									
Bergapten	[M-H]^−^	C_12_H_8_O_4_	215.03587	-	-	-	−4.1	0.6	-
Chamazulene	[M+Cl]^−^	C_14_H_16_	219.09499	-	-	-	-	−1.8	0.0
β-Tocopherol	[M-H]^−^	C_28_H_48_O_2_	415.35786	-	-	-	-	-	0.7

^a^ stands for theoretical mass-to-charge ratio. ^b^ the error expressed in parts per million (ppm).

**Table 2:** Number of molecular formulas (MF) identified by ESI FT-ICR MS in extracts of white and red tunicae and bulb of ‘Sulmona red garlic’ collected in 2020 and 2021 ^a^.

Extract	Ion Type	Identified MF			
		2020		2021	
White tunica	ESI (+)	270	341	322	414
	ESI (−)	106		121	
Red tunica	ESI (+)	362	417	267	340
	ESI (−)	106		101	
Bulb	ESI (+)	282	374	292	375
	ESI (−)	116		104	

^a^ Some compounds yield ion signals in both positive and negative ion modes, making the total number different from the sum of ESI (+) and ESI (−) identified compounds.

## Data Availability

Data are available within the manuscript and Supplementary Materials.

## Supplementary Materials

The following supporting information can be downloaded at: https://www.mdpi.com/article/10.3390/antiox11112088/s1, Table S1: Retention time, calibration curve and correlation coefficient of reference compounds; Figure S1: Chromatogram of standard compounds; Table S2: Extraction yield of obtained hydroalcoholic extracts; Table S3: CIEL*a*b parameters of the hydroalcoholic dried extracts; Figure S2: Reflectance curves; Table S4: DPPH analysis of the obtained extracts expressed as Inhibition %; Figure S3: Chromatograms related the different analyzed samples; Table S5: HPLC-DAD quantitative analysis; Figure S4: Comparison between experimental ESI(+) CID assay performed by using LTQ XL ion trap and reference data; Figure S5: Van Krevelen diagram obtained by ESI FT-ICR MS analysis of hydroalcoholic extracts from ‘Sulmona red garlic’; Figure S6: Enlargement of ESI(+) FT-ICR MS of red tunica 2021 extract; Figure S7: Comparison between experimental ESI(+) CID assay of peaks at *m*/*z* 178.0 performed by using LTQ XL ion trap and reference data; Figure S8: Scores plot of the PCA analyses of the FT-ICR MS data for white and red tunica, and bulb of ‘Sulmona red garlic’ extracts; Figure S9: Box plots of most statistically relevant features discovered in ESI(-) data set; Figure S10: Cell metabolic activity (MTS assay) of undifferentiated (monocytes) and differentiated (macrophages), after 24 h and 48 h; Figure S11: Generation of peroxynitrite by monocytes and macrophages after 24 h exposure; Table S6: Untargeted metabolic profiling of ‘Sulmona red garlic’ hydroalcoholic extracts by ESI(+) FT-ICR MS; Table S7: Untargeted metabolic profiling of ‘Sulmona red garlic’ hydroalcoholic extracts by ESI(-) FT-ICR MS.
