# Supplementary material for: Characterization and Valorization of ‘Sulmona Red Garlic’ Peels and Small Bulbs
Source: Antioxidants (Basel). 2022 Oct 22;11(11):2088. doi: 10.3390/antiox11112088 (PMC9686930; doi:10.3390/antiox11112088)
Supplement: Supplementary file 1 [file antioxidants-11-02088-s001.zip › antioxidants-1958246-supplementary.pdf]

Characterization and valorization of ‘Sulmona red garlic’ peels and small bulbs

Alba Lasalvia[a,†], Francesco Cairone[a,†], Stefania Cesa[a,*], Alessandro Maccelli[a], Maria Elisa Crestoni[a], Luigi Menghini[b], Simone Carradori[b,*], Beatrice Marinacci[b], Marialucia Gallorini[b], Osama Elsallabi[c,d], Mirko Pesce[c], Antonia Patruno[c]

[a]Department of Drug Chemistry and Technology, “Sapienza” University of Rome, P.le Aldo Moro 5, 00185 Rome, Italy.

[b]Department of Pharmacy, “G. d’Annunzio” University of Chieti-Pescara, via dei Vestini 31, 66100 Chieti, Italy.

[c]Department of Medicine and Science of Aging, "G. d'Annunzio" University of Chieti-Pescara, via dei Vestini 31, 66100 Chieti, Italy.

[d]Department of Biosciences and Nutrition, Karolinska Institutet, SE-141 57 Huddinge, Sweden.

Table S1. Retention time, calibration curve and correlation coefficient of reference compounds.

	**Retention time (min)**	**Calibration curve (µg/mL)**	**Correlation coefficient**
Gallic acid	4.86	y = 15.51x + 37.06	0.9987
Alliin	6.89	y = 6.35x + 50.34	0.9987
Catechin	15.48	y = 5.18 x - 24.29	0.9961
Chlorogenic acid	18.01	y =12.02x - 3.95	0.9991
Caffeic acid	19.01	y = 35.23x – 28.86	0.9989
Epicatechin	20.98	y = 2.47x + 58.32	0.9982
p-Coumaric acid	24.71	y = 42.12x – 19.25	0.9987
Cyanidin-3-rutinoside	27.01	y = 16.58x + 34.53	0.9987
Ferulic acid	27.38	y = 20.65x + 22.96	0.9993
Sinapic acid	27.91	y = 11.37x + 9.92	0.9987
Myricetin	32.40	y = 21,51x – 5.93	0.9991
Quercetin	37.78	y = 21.69x + 24.12	0.9995
Kaempferol	42.25	y = 25.94x + 27.50	0.9988

Figure S1. Chromatogram of standard compounds. Panel A: 1. Gallic acid; 2. Alliin; 3. Catechin; 4. Chlorogenic acid; 5. Caffeic acid; 6. Epicatechin; 7. *p*-Coumaric acid; 8. Cyanidin-3-rutinoside; 9. Ferulic acid; 10. Sinapic acid; Panel B 11. Myricetin; 12. Quercetin; 13. Kaempferol.

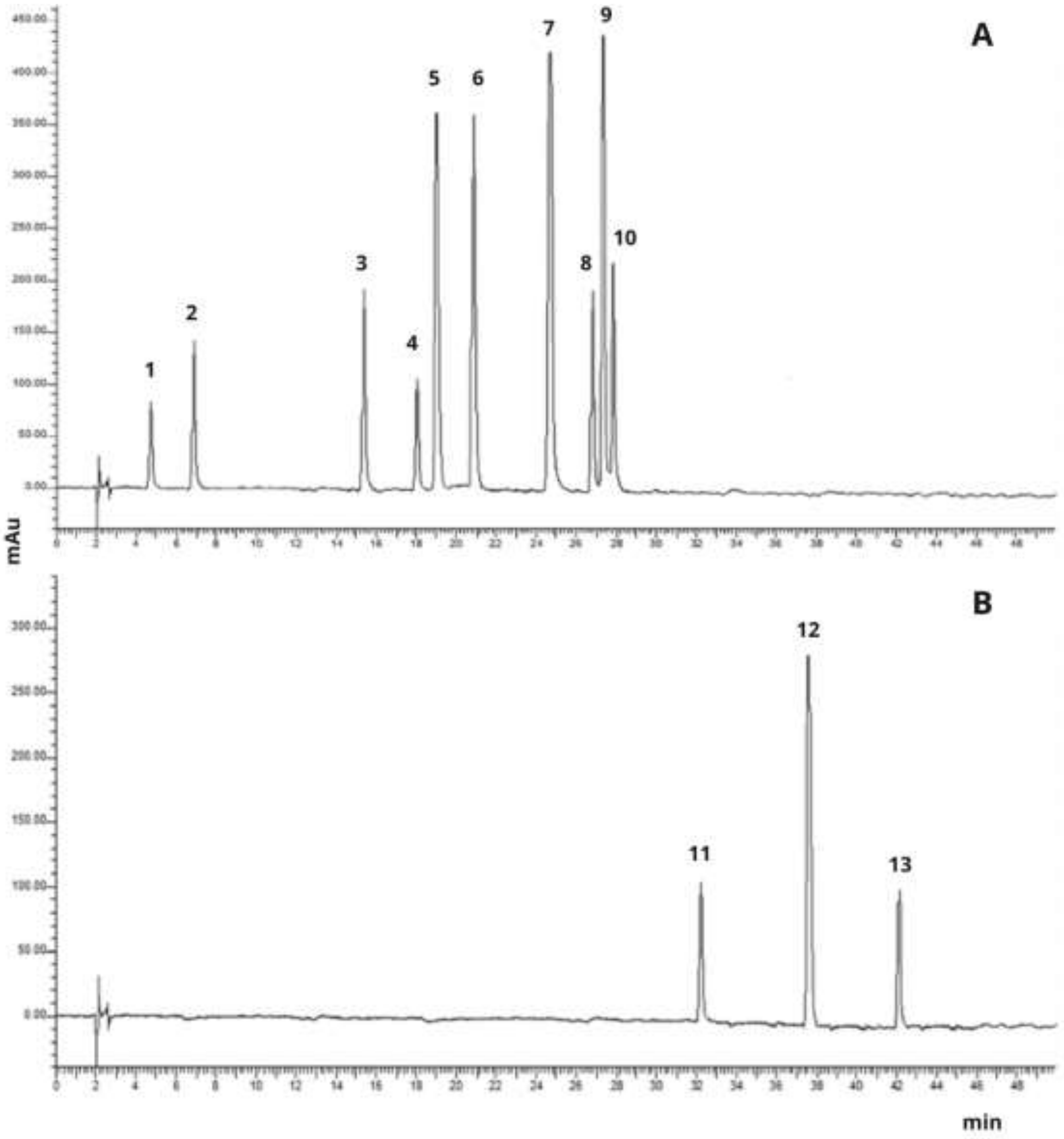

Table S2. Extraction yield of obtained hydroalcoholic extracts (p value < 0.002).

Harvest date	2020	2021
**Sample**	**Yield %**	
**W**	2.2 ± 0.5	3.3 ± 0.1
**R**	3.9 ± 0.5	5.2 ± 0.4
**B**	14.9 ± 1.9	17.9 ± 0.7

Table S3. CIEL*a*b parameters of the hydroalcoholic dried extracts. ^{a}p value ≤ 0.002; ^{b}p value ≤ 0.01; ^{c}p value ≤ 0.001; $^{a,b}p$ value ≤ 0.02; $^{a,c}p$ value ≤ 0.05; $^{a,c}p$ value ≤ 0.001.

Sample	Harvest	L*	a*	b*	C^*_{ab}	h_{ab}
**W**[a]	2020	73.1 ± 0.9	-0.5 ± 0.7	21.3 ± 5.4	21.4 ± 5.4	91.3 ± 1.6
	2021	70.8± 0.3	-2.2 ± 0.1	16.0 ± 0.4	16.1 ± 0.4	97.8 ± 0.7
**R**[b]	2020	61.8 ± 4.6	17.1 ± 2.6	6.5 ± 1.2	18.8 ± 1.6	23.2 ± 1.7
	2021	66.1 ± 0.1	8.3 ± 0.1	5.5 ± 0.1	10.0 ± 0.1	33.6 ± 0.1
**B**[c]	2020	72.4 ± 4.8	-1.7 ± 0.2	9.4 ± 2.3	9.5 ± 2.4	101.7 ± 5.1
	2021	76.1± 0.1	-1.7 ± 0.1	2.4 ± 0.1	2.97 ± 0.1	125.0 ± 2.3

Figure S2. Reflectance curves. Panel A, white tunica, 2020 and 2021; Panel B, red tunica, 2020 and 2021; Panel C; bulb, 2020 and 2021.

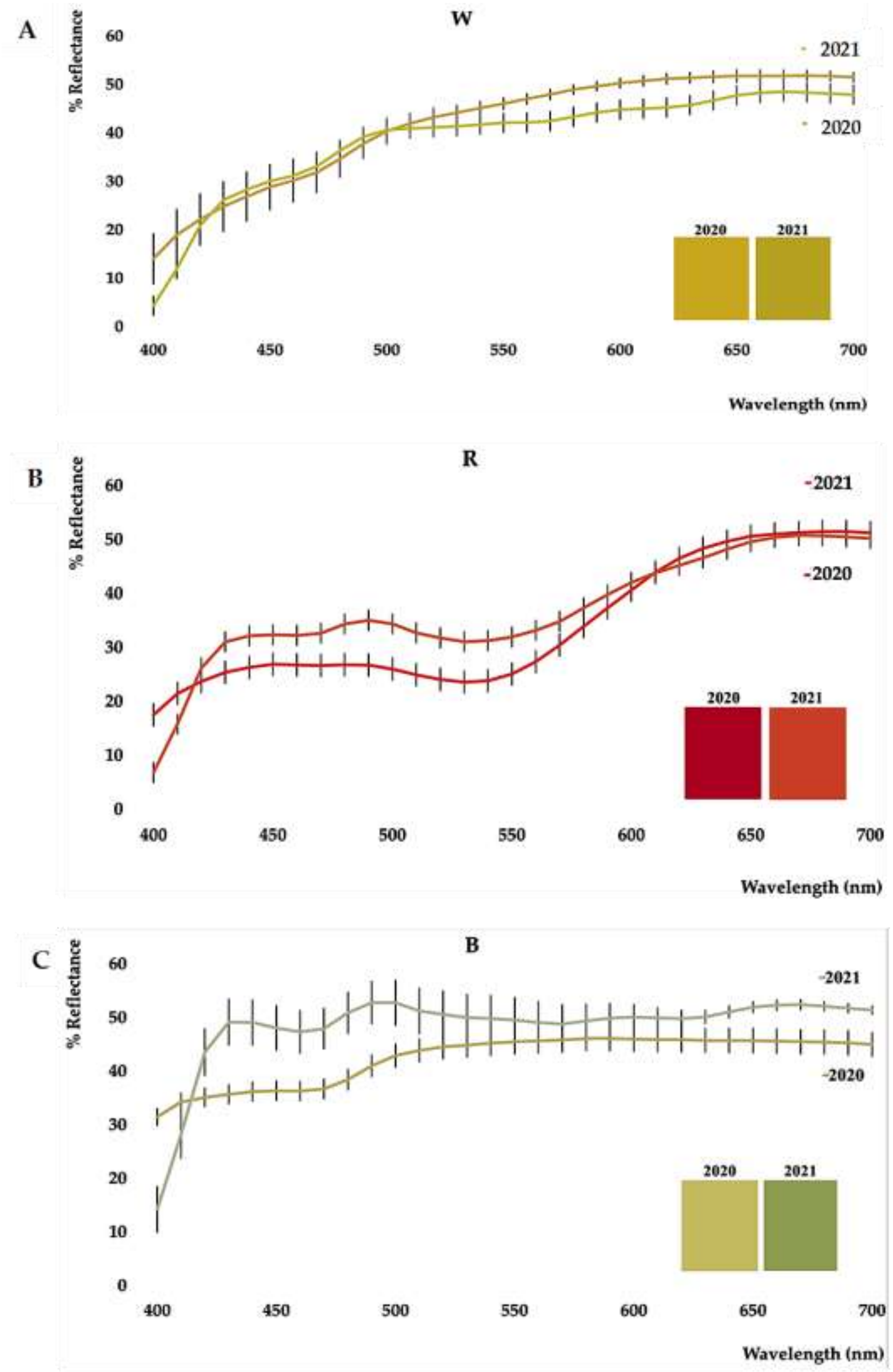

Table S4. DPPH analysis of the obtained extracts expressed as Inhibition % calculated as: Control Abs – Sample Abs/Control Abs x 100. p value ≤ 0.05.

**Harvest date**	**July 2020**	**July 2021**
**Sample**	**DPPH (Inhibition %)**	
W	24.19 ± 0.09	39.21 ± 2.50
R	19.76 ± 0.16	16.70 ± 1.14
B	3.25 ± 0.12	2.19 ± 0.04

Figure S3. Chromatograms related the different analyzed samples. Panel A (W-2020, W-2021); Panel B (R-2020; R-2021); Panel C (R-2020, R-2021 and B-2020, B-2021). Identified peaks: 1. Gallic acid; 2. Alliin; 3. Catechin; 4. Chlorogenic acid; 5. Caffeic acid; 6. Epicatechin; 7. *p*-coumaric acid; 8. Ferulic acid; 9. Sinapic acid; 10. Myricetin; 11. Quercetin; a. cinnamic acid derivative; b. kaempferol derivative; c,d,e. anthocyanins.

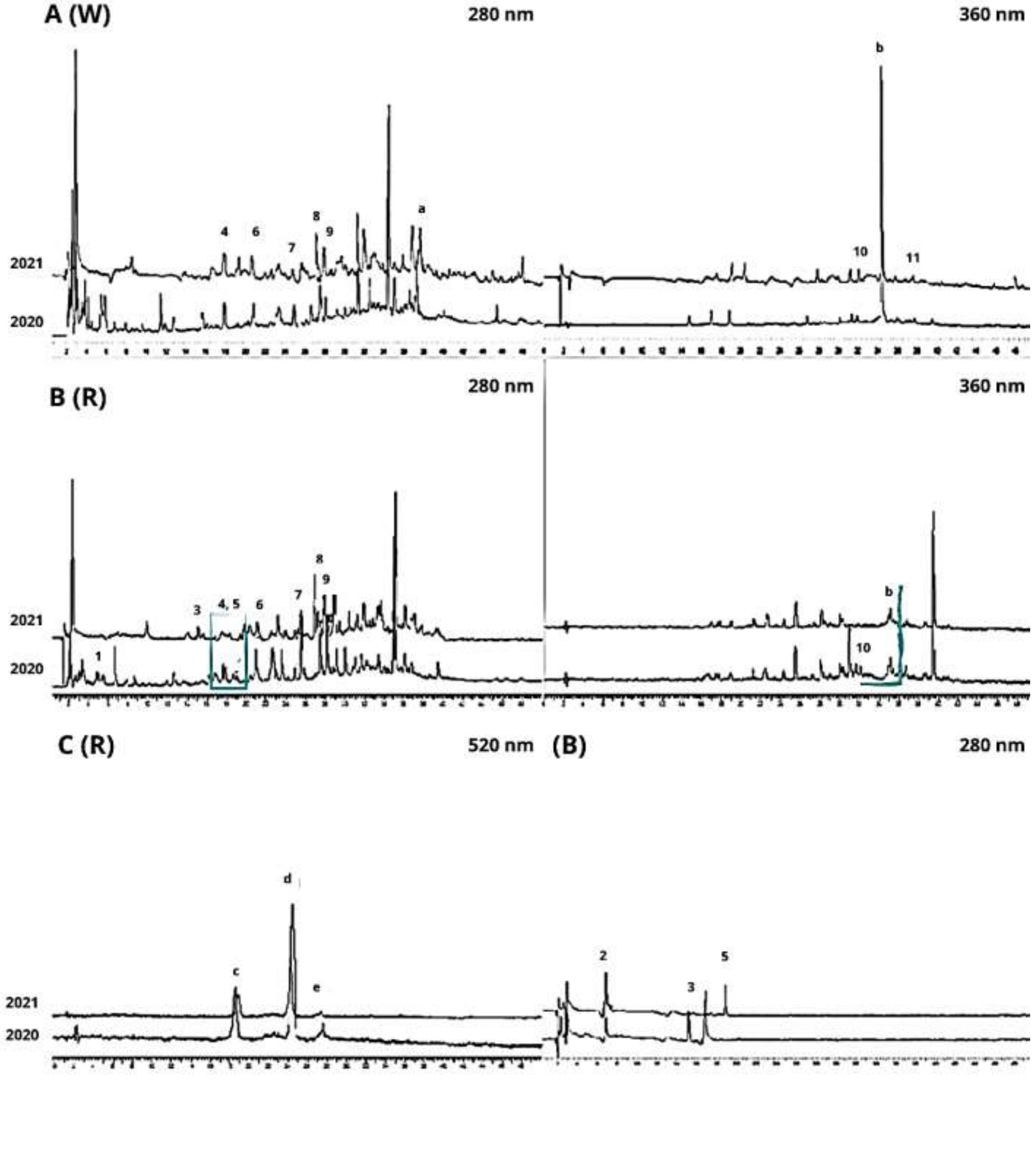

Table S5. HPLC-DAD quantitative analysis. Values are expressed as mg/g dry extract ± SD; NI: not identified; ---: not searched; *expressed as equivalents of cyanidin-3-rutinoside; **expressed as equivalents of Kaempferol; ***expressed as equivalents of *p*-coumaric acid. [a]*p* value ≤ 0.002; [b]*p* value ≤ 0.001; [c]*p* value ≤ 0.87; [a,b]*p* value ≤ 0.03; [a,c]*p* value ≤ 0.002; [b,c]*p* value ≤ 0.009.

	**White tunica (W)[a]**		**Red tunica (R)[b]**		**Bulb (B)[c]**	
	**2020**	**2021**	**2020**	**2021**	**2020**	**2021**
**Gallic acid**	NI	NI	0.03 ± 0.01	NI	NI	NI
**Alliin**	NI	NI	NI	NI	0.20 ± 0.03	0.26 ± 0.08
**Catechin**	NI	NI	NI	0.84 ± 0.02	2.05 ± 0.06	NI
**Chlorogenic acid**	0.32 ± 0.02	0.58 ± 0.03	NI	NI	NI	NI
**Caffeic acid**	NI	NI	0.21 ± 0.02	NI	NI	0.20 ± 0.06
**Epicatechin**	2.08 ± 0.10	1.56 ± 0.08	5.95 ± 0.07	2.29 ± 0.2	NI	NI
***p*-Coumaric acid**	0.10 ± 0.01	0.14 ± 0.01	0.08 ± 0.01	NI	NI	NI
**Anthocyanins***	---	---	2.50 ± 0.07	0.67 ± 0.02	---	---
**Ferulic acid**	0.29 ± 0.01	0.43 ± 0.02	0.92 ± 0.01	0.37 ± 0.03	NI	NI
**Sinapic acid**	0.32 ± 0.02	0.63 ± 0.03	0.40 ± 0.03	0.67 ± 0.003	NI	NI
**Myricetin**	0.08 ± 0.01	0.16 ± 0.01	0.10± 0.01	NI	NI	NI
**Kaempferol derivative****	1.64 ± 0.08	1.97 ± 0.15	0.80 ± 0.01	0.15 ± 0.01	NI	NI
**Quercetin**	0.04 ± 0.01	0.08 ± 0.01	NI	NI	NI	NI
**Cinnamic acid derivative*****	0.55 ± 0.03	0.54 ± 0.03	NI	NI	NI	NI

SD: standard deviation.

Figure S4. Comparison between experimental ESI(+) CID assay performed by using LTQ XL ion trap and reference data obtained from Metlin database has allowed to confirm the assignments of the following peaks: Panel A) anethole *m/z* 149; Panel B) ferulic acid *m/z* 195.

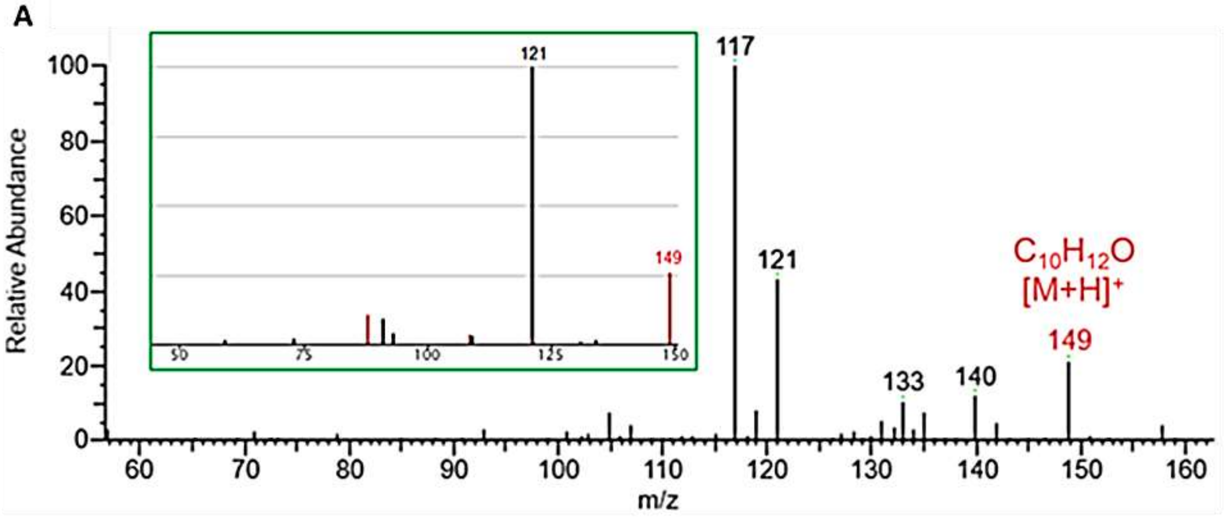

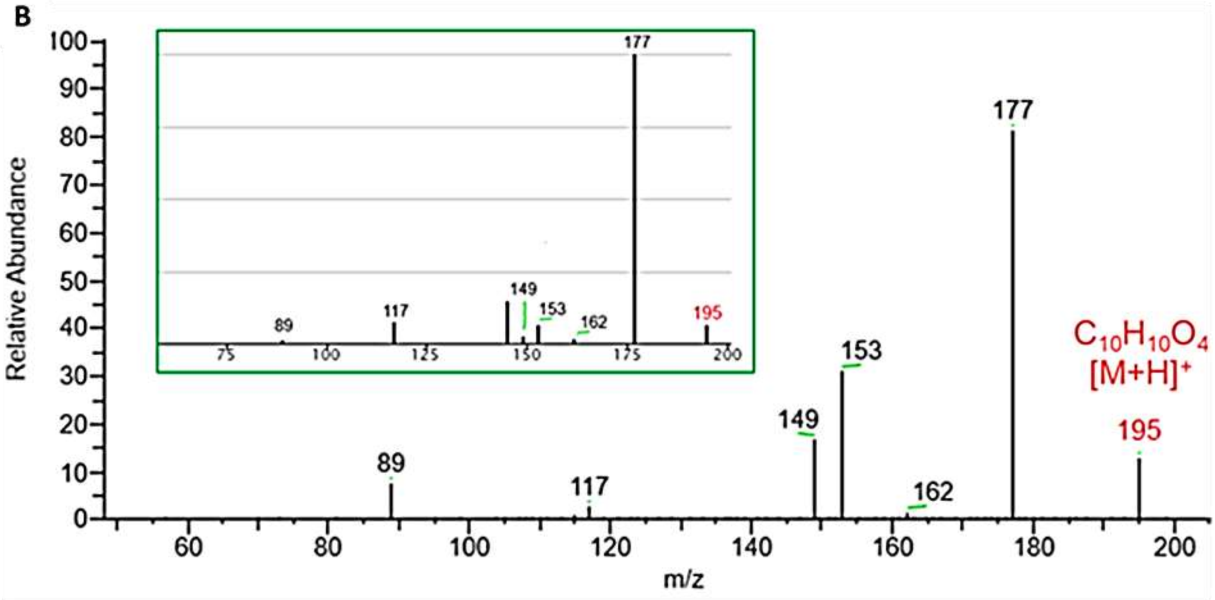

Figure S5. Van Krevelen diagram obtained by ESI FT-ICR MS analysis of hydroalcoholic extracts from ‘Sulmona red garlic’. The diagrams show broken lines with the following meaning: (A) lines related to (de)hydrogenation paths; (B) lines associated to oxidation or reduction reactions; (C) lines that describe hydration and condensation processes; (D) lines that correspond to (de)methylation paths. Noteworthy, peaks along A-lines include myristoleic ($C_{14}H_{26}O_2$)/myristic acids ($C_{14}H_{28}O_2$) for O/C= 0.14, palmitoleic ($C_{16}H_{30}O_2$)/palmitic ($C_{16}H_{32}O_2$) acids for O/C= 0.12; carnosol ($C_{20}H_{26}O_4$)/carnosic acid ($C_{20}H_{28}O_4$) for O/C= 0.2; items along B-lines encompass cysteine ($C_3H_7NO_2S$)/cysteic acid ($C_3H_7NO_5S$) for H/C= 2.3, oleic acid ($C_{18}H_{34}O_2$)/octadecadienoic acid ($C_{18}H_{34}O_4$) for H/C= 1.8, abscisic alcohol ($C_{15}H_{22}O_3$)/xanthoxic acid ($C_{15}H_{22}O_4$) for H/C= 1.4, ferulaldehyde ($C_{10}H_{10}O_3$)/ferulic acid ($C_{10}H_{10}O_4$) for H/C= 1; hits along D-lines include myristic ($C_{14}H_{28}O_2$)/palmitoleic ($C_{16}H_{30}O_2$)/linoleic ($C_{18}H_{32}O_2$) acids.

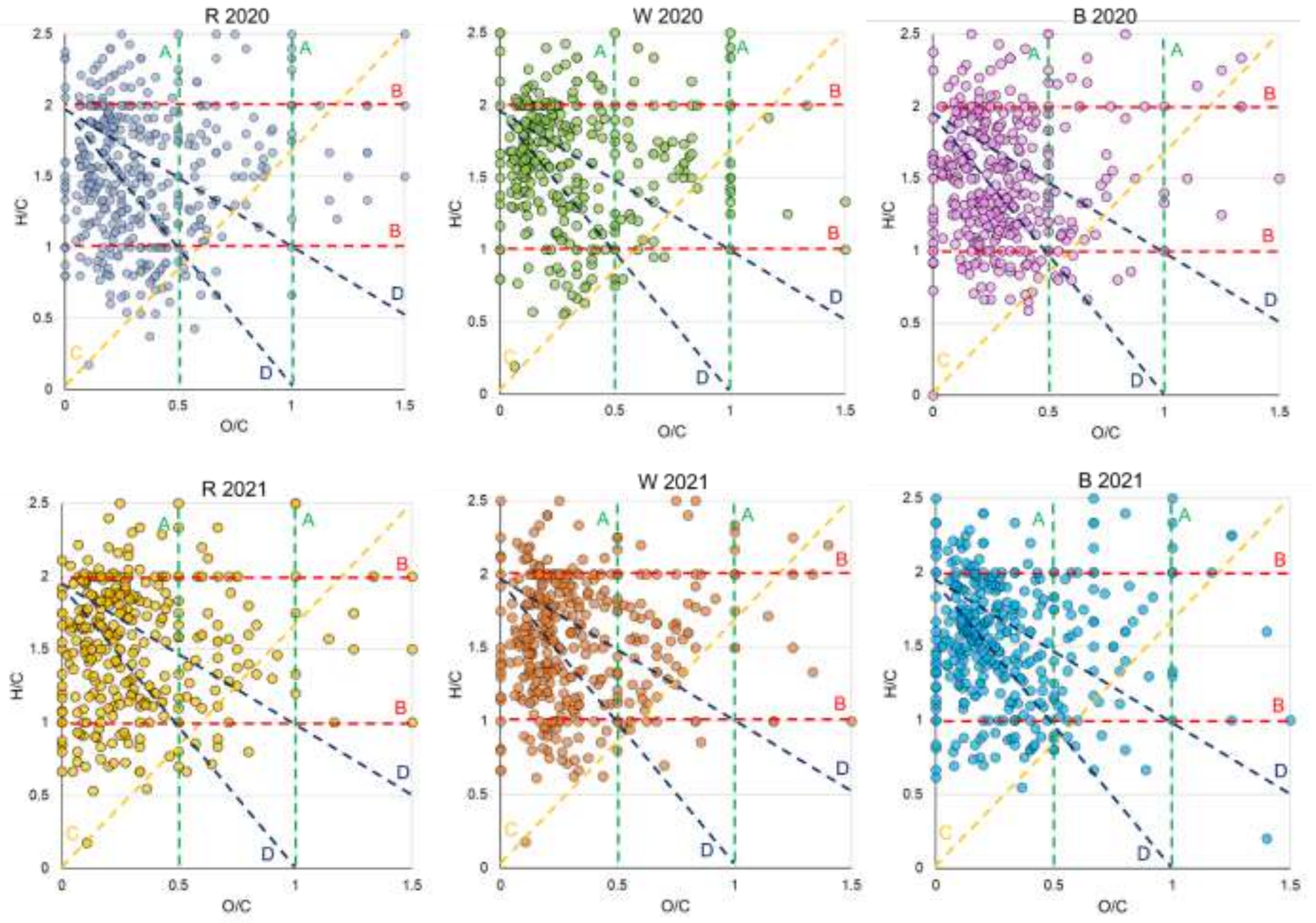

Figure S6. Enlargement of ESI(+) FT-ICR MS of red tunica 2021 extract. Spectrum shows protonated (*m*/*z* 178.05310) and sodiated (*m*/*z* 200.03512) adducts of metabolite with molecular formula $C_6H_{11}NO_3S$.

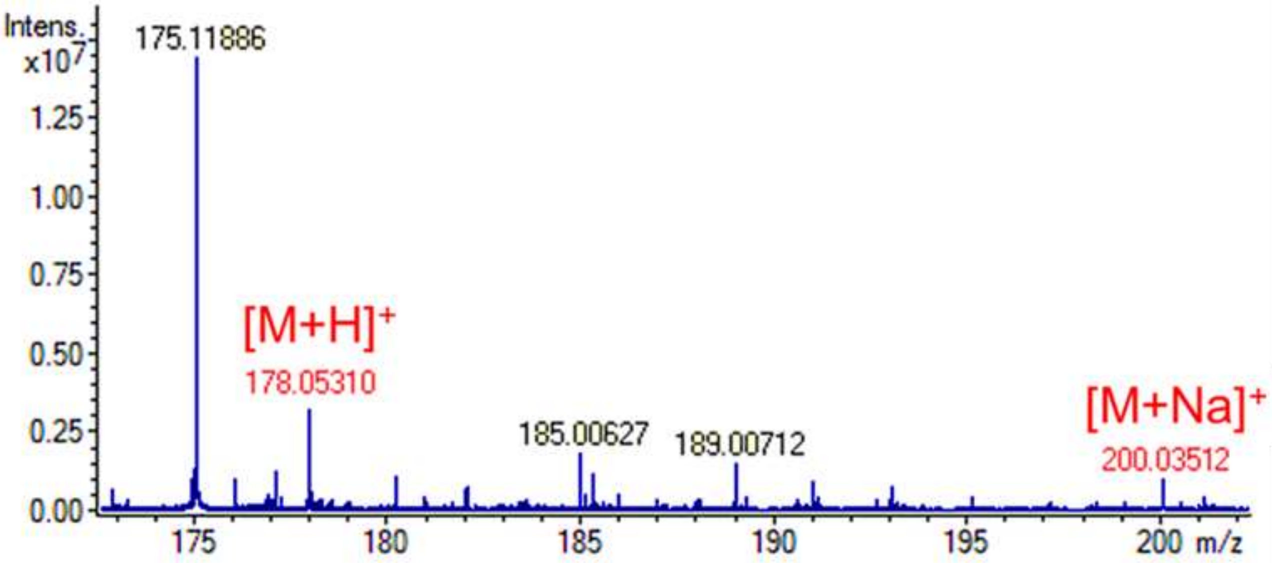

Figure S7. Comparison between experimental ESI(+) CID assay of peaks at *m*/*z* 178.0 performed by using LTQ XL ion trap and reference data obtained from Metlin database (green panel). Red fragments belonging to *N*-formylmethionine and blue fragments fit to $[C_3H_5NO_2+H]^+$ fragment present in both alliin and 1-PeCSO spectra.

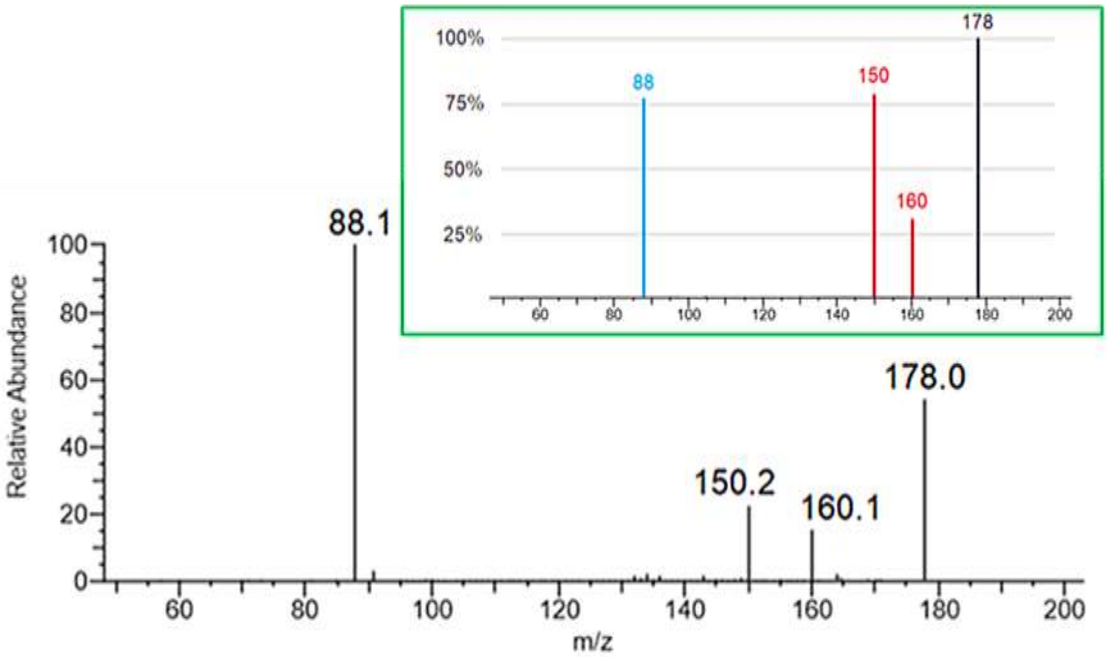

Figure S8. Panel A: Scores plot of the PCA analyses of the FT-ICR MS data for white and red tunica, and bulb of 'Sulmona red garlic' extracts. The firsts two components retain close to 70% of variation. in both cases. Dendograms, obtained on Pearson distances, are reported in panel B.

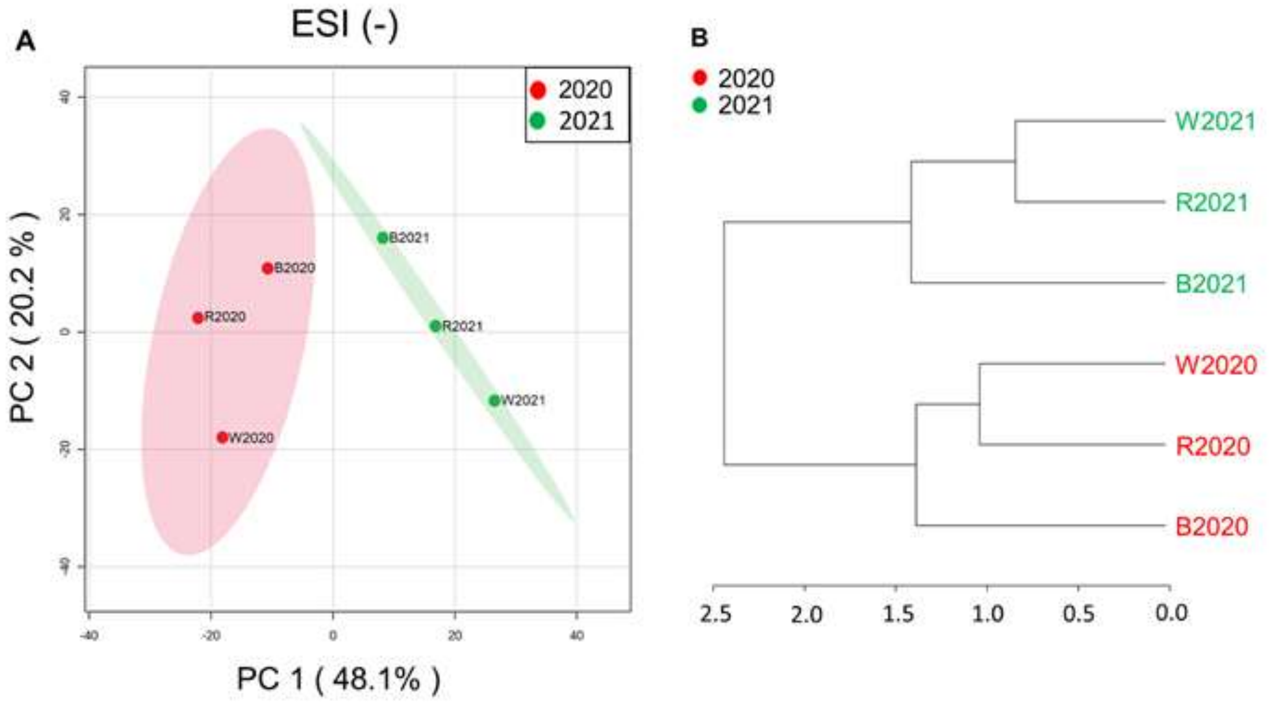

Figure S9. Box plots of most statistically relevant features discovered in ESI(-) data set. Box plots summarize the normalized concentrations and display that stearic and palmitic acids are variables more relevant for vintage 2020, as well as phosphatidic acid is more relevant for 2021. Positive polarity mode underlined (4-aminobutyl)guanidine ($[C_5H_{14}N_4+Na]^+$), isopropylmaleate ($[C_7H_{10}O_4+Na]^+$), fraxetin ($[C_{10}H_8O_5+H]^+$), butenylcarnitine ($[C_{11}H_{19}NO_4+H]^+$), falcarindiol ($[C_{17}H_{24}O_2+K]^+$) as the most significant features.

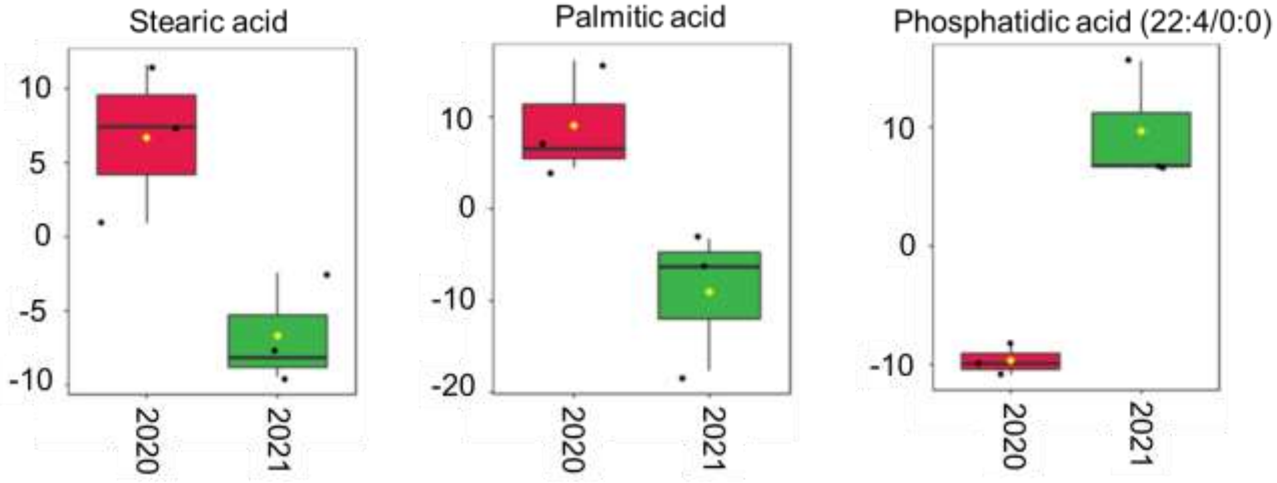

Figure S10. Cell metabolic activity (MTS assay) of undifferentiated (monocytes) and differentiated (macrophages), after 24 h and 48 h. THP-1 cells were assayed without differentiation or after differentiation as described in Materials and Methods. Undifferentiated and differentiated cells were untreated or treated with different doses (5, 10, 25, 50, 100 µg/mL) of garlic extracts (W: White Tunica; B: Bulb; R: Red Tunica). (Panel A) undifferentiated THP-1 cells at 24 h; (Panel B) differentiated THP-1 cells at 24 h; (Panel C) undifferentiated THP-1 cells and (Panel D) differentiated THP-1 cells at 48 h. The cells were stimulated with H_2O_2 as cytotoxicity model at concentration of 1 mM for 8 h. Data are expressed as the mean ± SD (*n*=3) and are represented as percentages of the untreated control (100% viability); *$p<0.05$ *vs* untreated cells (control); $^{\#}p<0.05$ *vs* H_2O_2 stimulation.

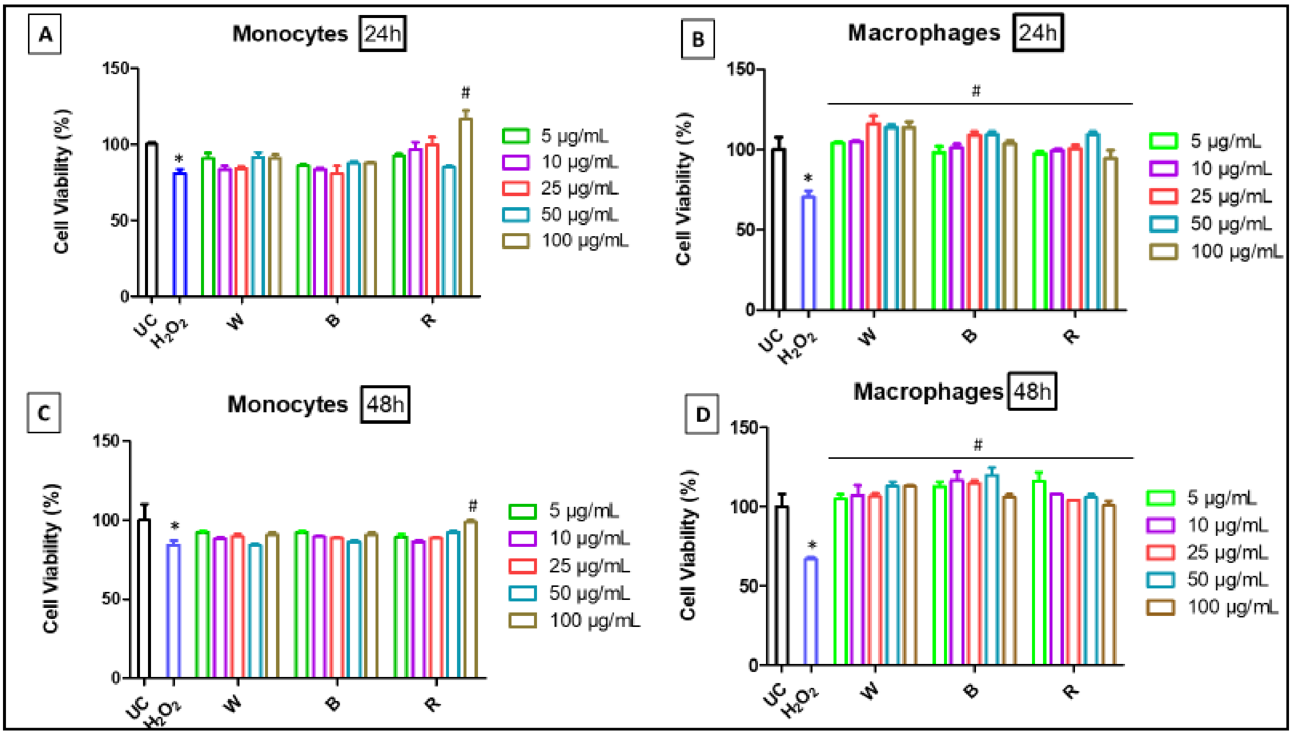

Figure S11. Generation of peroxynitrite by monocytes and macrophages after 24 h exposure. (Panels A, D) Histograms were obtained by flow cytometry and are generated by plotting the cell count (y-axis) and the FITC fluorescence emission (x-axis). (Panels B, C) Bar graphs show the fold increase of the mean fluorescence intensities (MFIs) related to the emissions in the FL-1/FITC channel which are proportional to the generation of peroxynitrite. Values are the ratios of the MFI generated from each sample on the unstained control (negative). a = $p < 0.01$, b = $p < 0.001$ and c = $p < 0.0001$ between treated samples and UC; d = $p < 0.01$, e= $p < 0.001$ and f= $p < 0.0001$ between samples and H_2O_2. UC = untreated control.

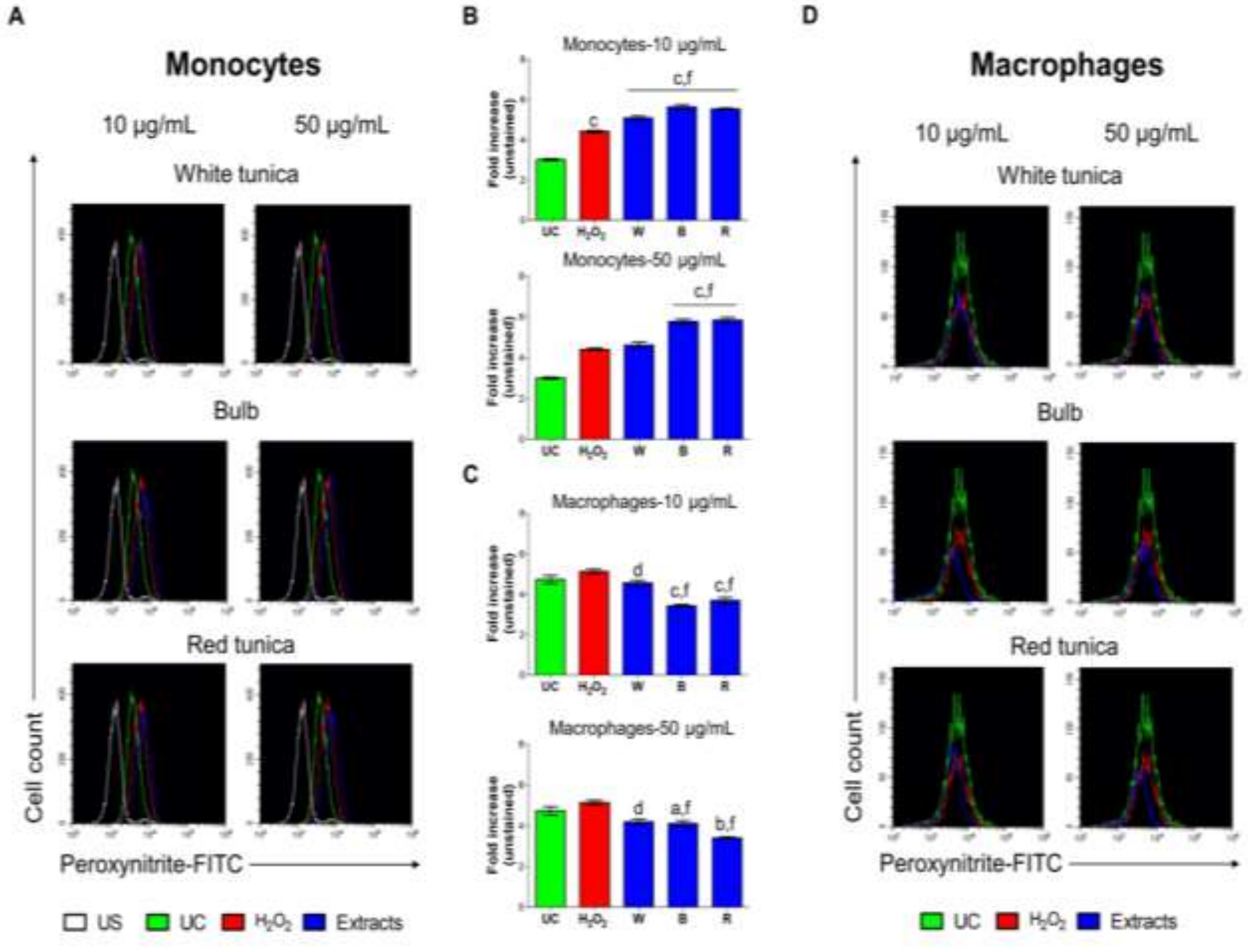

Table S6: Untargeted metabolic profiling of *Sulmona red garlic* hydroalcoholic extracts by ESI(+) FT-ICR MS

#	Putative Annotation [a]	Ion	Formula	$m/z_{Theo.}$ [b]	R2020[c]	ppm	W2020[c]	ppm	B2020[c]	ppm	R2021[c]	ppm	W2021[c]	ppm	B2021[c]	ppm
1	Dimethylamine	[M+K]+	C2H7N	84.02125					1.3E+05	-2.9						
2	Butynoate	[M+H]+	C4H4O2	85.02877					9.8E+04	-4.3						
3	Methanethiol	[M+K]+	CH4S	86.96611							1.2E+05	4.8			1.2E+05	3.2
4	Pentan-2-one	[M+H]+	C5H10O	87.08046							1.1E+05	-0.2				
5	Piperazine	[M+H]+	C4H10N2	87.09169			1.1E+05	-0.2								
6	Methylbutanamine	[M+H]+	C5H13N	88.11185											1.1E+05	2.6
7	Pyruvic acid	[M+H]+	C3H4O3	89.02329			1.2E+05	0.3	1.1E+05	4.8						
8	N,N'-Dimethylurea	[M+H]+	C3H8N2O	89.07051									1.6E+05	4.8		
9	Pentanol	[M+H]+	C5H12O	89.09632									1.7E+05	-2.6		
10	Oxamic acid	[M+H]+	C2H3NO3	90.01857	1.1E+05	-3.4			1.1E+05	-3.0						
11	Alanine	[M+H]+	C3H7NO2	90.05519			1.2E+05	-2.6							1.7E+05	5.0
12	N-(Aminomethyl)urea	[M+H]+	C2H7N3O	90.06611							8.1E+04	0.9				
13	Oxalic acid	[M+H]+	C2H2O4	91.00290											1.6E+05	-3.5
14	Propanethial S-oxide	[M+H]+	C3H6OS	91.02151			1.3E+05	-3.3					1.4E+05	2.1		
15	Imidazole	[M+Na]+	C3H4N2	91.02623											1.4E+05	4.8
16	Lactate	[M+H]+	C3H6O3	91.03855											1.6E+05	4.6
17	Diethyl sulfide	[M+H]+	C4H10S	91.05774			1.5E+05	-1.6								
18	Butanediol	[M+H]+	C4H10O2	91.07502			1.5E+05	3.7	8.5E+04	-2.7						
19	3-Aminopropane-1,2-diol	[M+H]+	C3H9NO2	92.07072					1.1E+05	-1.2	1.9E+05	-1.3			1.4E+05	3.3
20	Propynoic acid	[M+Na]+	C3H2O2	92.99443					7.6E+05	2.9					1.1E+05	0.5
21	Thioglycolic acid	[M+H]+	C2H4O2S	93.00057			2.3E+05	-1.0								
22	Butadiene	[M+K]+	C4H6	93.01011	9.6E+04	4.8							2.1E+05	4.8		
23	alpha-Aminopropiononitrile	[M+Na]+	C3H6N2	93.04197			1.2E+05	3.7								
24	3-Buten-1-amine	[M+Na]+	C4H9N	94.06277									9.6E+04	-0.5		
25	Aniline	[M+H]+	C6H7N	94.06507									1.0E+05	0.6		
26	Acrolein	[M+K]+	C3H4O	94.98940											1.4E+05	-0.3
27	Malondialdehyde	[M+Na]+	C3H4O2	95.00998					1.2E+05	3.9						
28	Butanal	[M+Na]+	C4H8O	95.04674	1.0E+05	3.6	1.5E+05	3.2								
29	Phenol	[M+H]+	C6H6O	95.04870									9.0E+04	4.6		
30	Aminopyridine	[M+H]+	C5H6N2	95.05993							2.1E+05	4.7				
31	N-Methylpyridinium	[M+H]+	C6H8N	95.07304											1.2E+05	-0.9
32	Iminoglycine	[M+Na]+	C2H3NO2	96.00542			1.6E+05	1.9					3.4E+05	-3.6		
33	Cyclopropylamine	[M+K]+	C3H7N	96.02115											1.3E+05	-1.5
34	Glyoxal	[M+K]+	C2H2O2	96.96886			2.7E+05	-2.3								
35	Glyoxylic acid	[M+Na]+	C2H2O3	96.99042											3.3E+05	-4.6
36	Methanesulfonic acid	[M+H]+	CH4SO3	96.99513			1.5E+05	2.7								
37	Lactaldehyde	[M+Na]+	C3H6O2	97.02574											1.8E+05	2.7
38	Propanoic acid	[M+Na]+	C3H6O2	97.02600	1.5E+05	-2.9										
39	Furfural	[M+H]+	C5H4O2	97.02841	1.3E+05	4.8										

#	Putative Annotation [a]	Ion	Formula	$m/z_{Theo.}$ [b]	R2020[c]	ppm	W2020[c]	ppm	B2020[c]	ppm	R2021[c]	ppm	W2021[c]	ppm	B2021[c]	ppm
**40**	2-Cyclohexen-1-one	[M+H]+	C6H8O	97.06512			1.6E+05	-3.4								
**41**	Aminoacetaldehyde	[M+K]+	C2H5NO	98.00039							1.4E+05	-1.2				
**42**	Guanidine	[M+K]+	CH5N3	98.01158					9.1E+04	-0.7						
**43**	Glycine	[M+Na]+	C2H5NO2	98.02139			1.8E+05	-1.4			1.2E+05	-2.9				
**44**	Isopropylamine	[M+K]+	C3H9N	98.03671							3.4E+05	-0.5	1.1E+05	4.6		
**45**	Deoxycytosine	[M+H]+	C4H7N3	98.07093									1.4E+05	3.5		
**46**	Sulfuric acid	[M+H]+	H2SO4	98.97540			1.3E+05	-0.4								
**47**	Thiourea	[M+Na]+	CH4N2S	98.99878											1.2E+05	-0.4
**48**	Glycolic acid	[M+Na]+	C2H4O3	99.00483							8.7E+04	4.4				
**49**	Hydroxyurea	[M+Na]+	CH4N2O2	99.01650	2.3E+05	0.3										
**50**	Propan-2-ol	[M+K]+	C3H8O	99.02067	8.7E+04	3.2										
**51**	Propyl mercaptan	[M+Na]+	C3H8S	99.02396			1.2E+05	-0.7								
**52**	Propane-1,2-diol	[M+Na]+	C3H8O2	99.04128					1.9E+05	3.7						
**53**	Imidazole-4-methanol	[M+H]+	C4H6N2O	99.05536											1.3E+05	-0.7
**54**	Guanidine	[M+K41]+	CH5N3	100.00917							1.2E+05	4.5				
**55**	Ethanolamine	[M+K]+	C2H7NO	100.01592	8.7E+04	1.4										
**56**	2-Piperidinone	[M+H]+	C5H9NO	100.07573											1.7E+05	-0.4
**57**	Cyclohexylamine	[M+H]+	C6H13N	100.11204											1.1E+05	0.4
**58**	Glycol	[M+K]+	C2H6O2	100.99994	6.8E+04	0.7										
**59**	Tiglic acid	[M+H]+	C5H8O2	101.05931			1.8E+05	3.9	4.4E+05	-4.6						
**60**	Hexanal	[M+H]+	C6H12O	101.09590									8.7E+04	1.9		
**61**	Pyridine	[M+Na]+	C5H5N	102.03137			1.5E+05	0.5			1.4E+05	-2.7	8.3E+04	-3.3	1.3E+05	4.3
**62**	1-Aminocyclopropane-1-carboxylate	[M+H]+	C4H7NO2	102.05460									1.3E+05	3.5	1.6E+05	4.8
**63**	5-Aminopentanal	[M+H]+	C5H11NO	102.09071											1.1E+05	5.0
**64**	Ethylenethiourea	[M+H]+	C3H6N2S	103.03287									8.5E+04	-4.1		
**65**	Succinic acid semialdehyde	[M+H]+	C4H6O3	103.03856											1.2E+05	4.0
**66**	N-Nitrosodiethylamine	[M+H]+	C4H10N2O	103.08690									9.4E+04	-3.0		
**67**	Hexanol	[M+H]+	C6H14O	103.11149									8.6E+04	2.4		
**68**	Cadaverine	[M+H]+	C5H14N2	103.12283							1.0E+05	1.4				
**69**	Sulfite	[M+Na]+	H2SO3	104.96205			1.6E+05	-3.5								
**70**	Allophanic acid	[M+H]+	C2H4N2O3	105.02947	7.6E+04	-1.9										
**71**	Cyanopyridine	[M+H]+	C6H4N2	105.04454									2.2E+05	1.8	9.5E+04	2.0
**72**	Hydroxybutanoic acid	[M+H]+	C4H8O3	105.05468							8.8E+04	-0.6				
**73**	Styrene	[M+H]+	C8H8	105.06979							1.3E+05	0.8				
**74**	3-Butyn-1-al	[M+K]+	C4H4O	106.98937	8.1E+04	1.5										
**75**	Isoprene	[M+K]+	C5H8	107.02576	9.0E+04	1.5									9.0E+04	0.0
**76**	Aminotriazole	[M+Na]+	C2H4N4	107.03329					1.2E+05	-4.4						
**77**	Glyceric acid	[M+H]+	C3H6O4	107.03381			1.0E+05	0.7	1.2E+05	4.6						
**78**	Cyclohexane	[M+Na]+	C6H12	107.08337											1.3E+05	-2.3
**79**	Iso-Valeraldehyde	[M+Na]+	C5H10O	109.06202			2.6E+05	3.4								

#	Putative Annotation [a]	Ion	Formula	$m/z_{Theo.}$ [b]	R2020[c]	ppm	W2020[c]	ppm	B2020[c]	ppm	R2021[c]	ppm	W2021[c]	ppm	B2021[c]	ppm
**80**	3-Buten-1-amine	[M+K]+	C4H9N	110.03670									8.7E+04	-0.4		
**81**	Malondialdehyde	[M+K]+	C3H4O2	110.98346			1.6E+05	5.0								
**82**	Resorcinol	[M+H]+	C6H6O2	111.04365			1.9E+05	3.7	4.3E+06	-3.5						
**83**	Putrescine	[M+Na]+	C4H12N2	111.08907									9.9E+04	1.8		
**84**	N-(Aminomethyl)urea	[M+Na]+	C2H7N3O	112.04786					2.9E+05	2.4						
**85**	Lactic acid	[M+Na]+	C3H6O3	113.02089			2.0E+05	0.2								
**86**	Furoic acid	[M+H]+	C5H4O3	113.02314					3.0E+05	1.6						
**87**	Butanol	[M+K]+	C4H10O	113.03625							8.1E+04	0.7	8.3E+04	5.0		
**88**	Butanediol	[M+Na]+	C4H10O2	113.05782			2.7E+05	-4.6								
**89**	Aleprolic acid	[M+H]+	C6H8O2	113.05971	1.1E+05	0.7										
**90**	2-heptenal	[M+H]+	C7H12O	113.09599			1.1E+05	0.9							1.1E+05	-4.5
**91**	epsilon-Caprolactam	[M+H]+	C6H11NO	114.09109			1.9E+05	2.2								
**92**	Glycerol	[M+Na]+	C3H8O3	115.03653			3.2E+05	0.3								
**93**	3-oxo-4-pentenoic acid	[M+H]+	C5H6O3	115.03917					3.1E+05	-1.7						
**94**	Hydrosorbic acid	[M+H]+	C6H10O2	115.07540			1.1E+05	-0.4								
**95**	3-Amino-2-piperidone	[M+H]+	C5H10N2O	115.08707							9.0E+04	-4.2				
**96**	Maleamic acid	[M+H]+	C4H5NO3	116.03413							9.2E+04	0.8	1.0E+05	-4.9		
**97**	Proline	[M+H]+	C5H9NO2	116.07060	4.8E+05	2.3	1.6E+05	-3.4								
**98**	Aminopentanamide	[M+H]+	C5H12N2O	117.10176									9.8E+04	4.1	1.4E+05	-3.0
**99**	Valine	[M+H]+	C5H11NO2	118.08626	1.9E+05	2.2	2.2E+05	2.4								
**100**	Pyrimidine	[M+K]+	C4H4N2	119.00065					1.3E+05	-0.4						
**101**	Sorbaldehyde	[M+Na]+	C6H8O	119.04662					1.1E+05	1.0						
**102**	Hydroxy-isovaleric acid	[M+H]+	C5H10O3	119.07118											1.0E+05	-1.9
**103**	2-Phenylpropene	[M+H]+	C9H10	119.08520											9.0E+04	2.7
**104**	Aminomalonic acid	[M+H]+	C3H5NO4	120.02913	7.9E+04	-1.6										
**105**	Hexenal	[M+Na]+	C6H10O	121.06195					2.6E+05	3.6						
**106**	Phenylethylamine	[M+H]+	C8H11N	122.09592									1.5E+06	4.1	2.3E+06	-4.4
**107**	Mercaptolactic acid	[M+H]+	C3H6O3S	123.01104	8.8E+04	-4.8										
**108**	Tiglic acid	[M+Na]+	C5H8O2	123.04165	7.0E+04	2.9	1.1E+05	3.7	4.5E+06	-4.8						
**109**	Benzoic acid	[M+H]+	C7H6O2	123.04455									1.7E+05	-4.0		
**110**	Erythritol	[M+H]+	C4H10O4	123.06519	1.0E+05	-3.0										
**111**	Nicotinic acid	[M+H]+	C6H5NO2	124.03930	8.7E+04	-3.3									1.5E+05	-3.1
**112**	Aminopentanal	[M+Na]+	C5H11NO	124.07361							7.5E+04	-2.6				
**113**	Valeric acid	[M+Na]+	C5H10O2	125.05650					9.4E+05	5.0						
**114**	Methylimidazole acetaldehyde	[M+H]+	C6H8N2O	125.07112			1.1E+05	-1.4								
**115**	Maltol	[M+H]+	C6H6O3	127.03937									2.1E+05	-3.1		
**116**	Thymine	[M+H]+	C5H6N2O2	127.05020	1.1E+05	1.2										
**117**	Pentanol	[M+K]+	C5H12O	127.05196											1.1E+05	0.1
**118**	5-Amino-4-imidazolecarboxyamide	[M+H]+	C4H6N4O	127.06206									9.5E+04	-4.9		
**119**	2-octenal	[M+H]+	C8H14O	127.11140							7.8E+04	2.7				

#	Putative Annotation [a]	Ion	Formula	$m/z_{Theo.}$ [b]	R2020[c]	ppm	W2020[c]	ppm	B2020[c]	ppm	R2021[c]	ppm	W2021[c]	ppm	B2021[c]	ppm
**120**	Coniine	[M+H]+	C8H17N	128.14338	7.4E+04	1.4										
**121**	6-hydroxy-2-hexynoic acid	[M+H]+	C6H8O3	129.05461									3.4E+05	0.1		
**122**	Azulene	[M+H]+	C10H8	129.07017			2.0E+05	-2.3								
**123**	3-isopropyl-3-butenoic acid	[M+H]+	C7H12O2	129.09132							7.5E+04	-2.4				
**124**	3-Aminopropane-1,2-diol	[M+K]+	C3H9NO2	130.02649	1.2E+05	3.6										
**125**	Oxoproline	[M+H]+	C5H7NO3	130.04987	4.1E+05	0.6										
**126**	Pipecolic acid	[M+H]+	C6H11NO2	130.08626	1.6E+05	0.2										
**127**	Dimethylbiguanide	[M+H]+	C4H11N5	130.10874							8.9E+04	-0.1				
**128**	Octylamine	[M+H]+	C8H19N	130.15860			2.8E+05	3.3								
**129**	Leucine	[M+H]+	C6H13NO2	132.10191	8.0E+05	1.0	2.5E+05	-1.3								
**130**	Resorcinol	[M+Na]+	C6H6O2	133.02522					9.3E+04	5.0						
**131**	Imidazole acetaldehyde	[M+Na]+	C5H6N2O	133.03699									2.0E+06	1.8		
**132**	Asparagine	[M+H]+	C4H8N2O3	133.06077	3.7E+05	-0.3										
**133**	Dicyclopentadiene	[M+H]+	C10H12	133.10118	1.2E+05	-2.8										
**134**	Aminomethylphosphonic acid	[M+Na]+	CH6NO3P	133.99754									7.5E+04	1.6		
**135**	Hydroxypyridine	[M+K]+	C5H5NO	134.00012											1.0E+05	1.1
**136**	Methylthiobutanaldoxime	[M+H]+	C5H11NOS	134.06341	1.2E+05	-1.5										
**137**	Tetrahydroisoquinoline	[M+H]+	C9H11N	134.09636											8.5E+04	0.5
**138**	Malic acid	[M+H]+	C4H6O5	135.02885							1.6E+05	-0.4	1.7E+05	-4.8		
**139**	Ureidoglycolate	[M+H]+	C3H6N2O4	135.04042					1.0E+05	-2.9						
**140**	Adenine	[M+H]+	C5H5N5	136.06177	6.9E+04	2.6										
**141**	epsilon-Caprolactam	[M+Na]+	C6H11NO	136.07338							7.0E+04	-0.7				
**142**	Phenylacetamide	[M+H]+	C8H9NO	136.07543									2.1E+05	1.9		
**143**	Hydrosorbic acid	[M+Na]+	C6H10O2	137.05679			1.6E+05	3.7								
**144**	Vinylcatechol	[M+H]+	C8H8O2	137.05930									2.7E+05	3.0		
**145**	Tetrahydropteridine	[M+H]+	C6H8N4	137.08217	7.6E+04	-0.2										
**146**	Myrcene	[M+H]+	C10H16	137.13305					7.6E+05	-4.2						
**147**	Succinimide	[M+K]+	C4H5NO2	137.99560			1.0E+05	-3.0								
**148**	Proline	[M+Na]+	C5H9NO2	138.05177			1.3E+05	1.8								
**149**	Trigonelline	[M+H]+	C7H7NO2	138.05495	1.1E+05	3.9							1.4E+05	0.8		
**150**	Tyramine	[M+H]+	C8H11NO	138.09120									2.6E+05	1.0		
**151**	Tiglic acid	[M+K]+	C5H8O2	139.01474							8.5E+04	1.8				
**152**	Levulinic acid	[M+Na]+	C5H8O3	139.03652	1.6E+05	0.3					1.2E+05	-3.9				
**153**	Hydroxybenzoic acid	[M+H]+	C7H6O3	139.03869									8.3E+05	2.0		
**154**	Urocanic acid	[M+H]+	C6H6N2O2	139.05020	1.1E+06	0.7	5.8E+05	2.6								
**155**	Caproaldehyde	[M+K]+	C6H12O	139.05144							1.4E+05	3.8				
**156**	Aspartate-semialdehyde	[M+Na]+	C4H7NO3	140.03144			2.0E+05	2.7								
**157**	Methylaminoethylphosphonate	[M+H]+	C3H10NO3P	140.04714											7.5E+04	-0.2
**158**	Aminopentanal	[M+K]+	C5H11NO	140.04790			1.4E+05	-4.8								
**159**	Valine	[M+Na]+	C5H11NO2	140.06776			3.2E+05	3.1								

#	Putative Annotation [a]	Ion	Formula	$m/z_{Theo.}$ [b]	R2020 [c]	ppm	W2020 [c]	ppm	B2020 [c]	ppm	R2021 [c]	ppm	W2021 [c]	ppm	B2021 [c]	ppm
**160**	Succinic acid	[M+Na]+	C4H6O4	141.01619			1.3E+05	-2.6								
**161**	N-Nitrosodiethylamine	[M+K]+	C4H10N2O	141.04247	7.8E+04	1.5										
**162**	Hydroxymethylcatechol	[M+H]+	C7H8O3	141.05430			1.7E+05	2.3								
**163**	Octadienoic acid	[M+H]+	C8H12O2	141.09065									3.9E+06	2.5		
**164**	Diaminopropanoate	[M+K]+	C3H8N2O2	143.02174	7.0E+04	-1.0										
**165**	Erythrose	[M+Na]+	C4H8O4	143.03145											7.3E+04	0.2
**166**	Purine	[M+Na]+	C5H4N4	143.03281							7.1E+04	0.1	3.1E+05	-0.6		
**167**	7-hydroxy-5-heptynoic acid	[M+H]+	C7H10O3	143.07027	1.1E+05	-1.8										
**168**	Octenoic acid	[M+H]+	C8H14O2	143.10736							1.7E+05	-4.9				
**169**	Proline betaine	[M+H]+	C7H13NO2	144.10147			1.4E+05	3.0	8.3E+04	-3.4						
**170**	Glyceric acid	[M+K]+	C3H6O4	144.98936			1.6E+05	2.8	9.8E+04	4.1			1.1E+05	-3.1		
**171**	Mercaptolactic acid	[M+Na]+	C3H6O3S	144.99299	1.9E+05	-4.4										
**172**	Nicotinamide	[M+Na]+	C6H6N2O	145.03666					9.8E+04	4.0						
**173**	Erythritol	[M+Na]+	C4H10O4	145.04713	8.3E+04	4.9					1.1E+05	-4.1			1.9E+05	0.6
**174**	n-valeryl acetic acid	[M+H]+	C7H12O3	145.08636							9.4E+04	-3.0				
**175**	4-Amino-1-piperidinecarboxylic acid	[M+H]+	C6H12N2O2	145.09683											7.3E+04	2.2
**176**	Guanidinobutanamide	[M+H]+	C5H12N4O	145.10792							8.0E+04	3.2				
**177**	1-(3-Aminopropyl)-4-aminobutanal	[M+H]+	C7H16N2O	145.13295											7.8E+04	4.1
**178**	Allysine	[M+H]+	C6H11NO3	146.08079											8.2E+04	2.6
**179**	Orcinol	[M+Na]+	C7H8O2	147.04204					9.4E+04	-2.7						
**180**	N-Propanoylimidazole	[M+Na]+	C6H8N2O	147.05218									4.9E+05	4.8		
**181**	Glutamine	[M+H]+	C5H10N2O3	147.07642	1.3E+06	0.5										
**182**	2,4-octadienal	[M+Na]+	C8H12O	147.07795							6.3E+04	0.6				
**183**	Lysine	[M+H]+	C6H14N2O2	147.11280	6.2E+05	0.2	1.2E+05	-0.5	3.3E+05	3.8						
**184**	Glutamic acid	[M+H]+	C5H9NO4	148.06043	4.3E+05	1.5										
**185**	Dihydroxyfumaric acid	[M+H]+	C4H4O6	149.00745							1.7E+06	4.1	2.5E+06	-2.7		
**186**	Anethole	[M+H]+	C10H12O	149.09609											1.1E+05	2.2
**187**	Trihydroxypyridine	[M+Na]+	C5H5NO3	150.01602			1.9E+05	1.0								
**188**	Methionine	[M+H]+	C5H11NO2S	150.05893											1.7E+05	-4.0
**189**	Triethanolamine	[M+H]+	C6H15NO3	150.11247	2.8E+05	1.5										
**190**	Sorbic acid	[M+K]+	C6H8O2	151.01550			1.5E+05	0.6								
**191**	Ribose	[M+H]+	C5H10O5	151.06010	2.5E+05	4.1										
**192**	Dipropyl disulfide	[M+H]+	C6H14S2	151.06091	2.5E+05	-1.6			4.6E+04	0.5					7.6E+04	0.4
**193**	Methylhypoxanthine	[M+H]+	C6H6N4O	151.06208							3.5E+05	-4.3				
**194**	3-isopropyl-3-butenoic acid	[M+Na]+	C7H12O2	151.07271			1.3E+05	1.6								
**195**	Hydrocinnamic acid	[M+H]+	C9H10O2	151.07536	1.9E+05	3.7										
**196**	Carvacrol	[M+H]+	C10H14O	151.11174	1.7E+05	-2.9	1.5E+05	1.7								
**197**	Sulfinylpyruvic acid	[M+H]+	C3H3O5S	151.97715					1.0E+05	1.6						
**198**	Cyanuric acid	[M+Na]+	C3H3N3O3	152.00666	8.9E+04	2.6										
**199**	Pyroglutamic acid	[M+Na]+	C5H7NO3	152.03181	1.4E+05	-3.6	1.0E+05	-2.3								

#	Putative Annotation [a]	Ion	Formula	$m/z_{Theo.}$ [b]	R2020[c]	ppm	W2020[c]	ppm	B2020[c]	ppm	R2021[c]	ppm	W2021[c]	ppm	B2021[c]	ppm
**200**	epsilon-Caprolactam	[M+K]+	C6H11NO	152.04756									1.4E+05	-2.2		
**201**	Guanine	[M+H]+	C5H5N5O	152.05669	1.0E+05	3.1										
**202**	Phenylpropanolamine	[M+H]+	C9H13NO	152.10699	1.1E+05	1.0										
**203**	2-Hydroxy-2,4-pentadienoate	[M+K]+	C5H6O3	152.99512			1.1E+05	-1.7								
**204**	Allyl sulfide	[M+K]+	C6H10S	153.01305			9.2E+04	2.8								
**205**	Alloxanthine	[M+H]+	C5H4N4O2	153.04002											1.2E+05	4.5
**206**	3-Amino-2-piperidone	[M+K]+	C5H10N2O	153.04290							8.9E+04	-2.8				
**207**	2-keto-n-caproic acid	[M+Na]+	C6H10O3	153.05204			1.8E+05	1.1			9.2E+04	-0.1			8.2E+04	-2.1
**208**	Vanillin	[M+H]+	C8H8O3	153.05405					2.5E+06	3.7						
**209**	(4-Aminobutyl) guanidine	[M+Na]+	C5H14N4	153.11045							6.2E+05	4.0	6.9E+05	2.9	5.0E+05	2.9
**210**	Myrtenol	[M+H]+	C10H16O	153.12752					9.2E+05	-0.8						
**211**	Thiocysteine	[M+H]+	C3H7NO2S2	153.99877											9.1E+04	2.1
**212**	Creatine	[M+Na]+	C4H9N3O2	154.05864					1.6E+05	0.4			7.2E+04	3.0		
**213**	N-Dimethyl-2-aminoethylphosphonate	[M+H]+	C4H12NO3P	154.06276	1.8E+05	4.6									3.8E+05	-4.4
**214**	Leucine	[M+Na]+	C6H13NO2	154.08385	7.0E+04	-4.6	1.3E+05	3.4								
**215**	Vanillylamine	[M+H]+	C8H11NO2	154.08595											1.1E+05	2.0
**216**	N-Carbamoylputrescine	[M+Na]+	C5H13N3O	154.09538					6.0E+05	-1.9						
**217**	N-Guanylhistamine	[M+H]+	C6H11N5	154.10839											1.9E+05	2.2
**218**	Glutaric acid	[M+Na]+	C5H8O4	155.03177			1.6E+05	-1.9							6.2E+04	-4.1
**219**	Gentisic acid	[M+H]+	C7H6O4	155.03307					4.4E+05	4.2	9.0E+04	4.7				
**220**	Pteridine	[M+Na]+	C6H4N4	155.03310									3.1E+05	-1.8		
**221**	Leucinic acid	[M+Na]+	C6H12O3	155.06839							7.5E+04	-3.4				
**222**	Ornithine	[M+Na]+	C5H12N2O2	155.07815					8.2E+05	4.9						
**223**	Boschnialactone	[M+H]+	C9H14O2	155.10666	8.8E+04	4.9							1.2E+05	3.3		
**224**	Indole	[M+K]+	C8H7N	156.02142							6.1E+04	-2.6				
**225**	Valine	[M+K]+	C5H11NO2	156.04214	7.1E+04	0.1										
**226**	Indolin-2-one	[M+Na]+	C8H7NO	156.04215			9.4E+04	-1.1								
**227**	Valine	[M+K]+	C5H11NO2	156.04215			9.4E+04	-0.1								
**228**	Histidine	[M+H]+	C6H9N3O2	156.07675	2.6E+05	1.2										
**229**	Ketomalonic acid	[M+K]+	C3H2O5	156.95338	6.8E+04	2.1										
**230**	Phosphoglycolic acid	[M+H]+	C2H5O6P	156.98929							7.4E+04	2.3				
**231**	Malic acid	[M+Na]+	C4H6O5	157.01074	1.3E+05	-3.2										
**232**	S-Dimethylsulfonium propionic acid	[M+Na]+	C5H10O2S	157.02990							6.9E+04	-3.4	1.3E+05	-0.4		
**233**	Dihydropteridine	[M+Na]+	C6H6N4	157.04804			1.2E+05	2.7								
**234**	Nonane-4,6-dione	[M+H]+	C9H16O2	157.12248									8.5E+04	-1.1		
**235**	Nitrosonaphthalene	[M+H]+	C10H7NO	158.05933							6.2E+04	4.5				
**236**	Purine	[M+K]+	C5H4N4	159.00676	1.3E+05	2.2										
**237**	Maleylacetate	[M+H]+	C6H6O5	159.02848									8.7E+04	2.0		
**238**	1,4-Naphthoquinone	[M+H]+	C10H6O2	159.04406	8.5E+04	-1.3										
**239**	Methylerythritol	[M+Na]+	C5H12O4	159.06278	1.5E+05	4.7									1.5E+05	-3.0

#	Putative Annotation [a]	Ion	Formula	$m/z_{Theo.}$ [b]	R2020[c]	ppm	W2020[c]	ppm	B2020[c]	ppm	R2021[c]	ppm	W2021[c]	ppm	B2021[c]	ppm
**240**	Tetrahydropteridine	[M+Na]+	C6H8N4	159.06409							1.9E+05	0.2				
**241**	Nonatrienal	[M+Na]+	C9H12O	159.07804	9.7E+04	-1.0										
**242**	1-decanol	[M+H]+	C10H22O	159.17427											1.5E+05	0.5
**243**	Cysteine	[M+K]+	C3H7NO2S	159.98244					8.0E+04	2.9						
**244**	Trigonelline	[M+Na]+	C7H7NO2	160.03690	9.2E+04	0.6										
**245**	Phenylethylamine	[M+K]+	C8H11N	160.05232			9.5E+04	-0.1								
**246**	4-Methylene-L-glutamate	[M+H]+	C6H9NO4	160.06033									6.0E+04	0.7		
**247**	Dimethylaniline-N-oxide	[M+Na]+	C8H11NO	160.07328	9.1E+04	1.8										
**248**	Aminopropylcadaverine	[M+H]+	C8H21N3	160.18115											7.2E+04	-2.0
**249**	Isonicotineamide	[M+K]+	C6H6N2O	161.01117	3.4E+05	2.7										
**250**	Urocanic acid	[M+Na]+	C6H6N2O2	161.03157							8.4E+04	3.6				
**251**	Hydroxypurine	[M+Na]+	C5H6N4O	161.04272									1.1E+05	4.1		
**252**	Diaminotoluene	[M+K]+	C7H10N2	161.04756	2.5E+05	-2.6										
**253**	Dehydrocarnitine	[M+H]+	C7H14NO3	161.10453					3.0E+05	0.7						
**254**	S-Allyl-L-cysteine	[M+H]+	C6H11NO2S	162.05833	1.4E+05	-4.1										
**255**	Carnitinamide	[M+H]+	C7H17N2O2	162.13692					1.5E+05	-4.0						
**256**	Methylimidazole acetaldehyde	[M+K]+	C6H8N2O	163.02682	9.6E+05	4.2										
**257**	Umbelliferone	[M+H]+	C9H6O3	163.03897	2.1E+06	0.9										
**258**	Octadienoic acid	[M+Na]+	C8H12O2	163.07348			1.5E+05	-3.2			8.4E+04	-1.2	1.8E+05	-1.6		
**259**	Safrole	[M+H]+	C10H10O2	163.07545					1.3E+06	-0.6						
**260**	Nicotine	[M+H]+	C10H14N2	163.12297	1.1E+06	1.5										
**261**	Taurine	[M+K]+	C2H7NO3S	163.97782	3.9E+05	-4.9										
**262**	2-Aminoethylphosphonate	[M+K]+	C2H8NO3P	163.98721			7.1E+05	0.8								
**263**	Aminomuconate semialdehyde	[M+Na]+	C6H7NO3	164.03181	9.1E+04	-4.1					6.5E+04	3.9	1.2E+05	2.8		
**264**	Histidinol	[M+Na]+	C6H11N3O	164.07994					1.6E+05	-3.1						
**265**	N-Methylconiine	[M+Na]+	C9H19N	164.14109							1.1E+05	-0.7	1.0E+05	-1.1	2.0E+05	1.5
**266**	5-Amino-4-imidazolecarboxyamide	[M+K]+	C4H6N4O	165.01732	1.1E+05	-2.7										
**267**	Coumaric acid	[M+H]+	C9H8O3	165.05462	2.3E+05	-1.4	6.3E+05	3.2	2.0E+06	4.6	4.2E+05	1.1	1.5E+06	-4.2	5.3E+05	3.1
**268**	2,4-Dimethyl-2E,4E-hexadien-1-ol	[M+K]+	C8H14O	165.06790							4.7E+05	-1.7				
**269**	Trihydroxypyridine	[M+K]+	C5H5NO3	165.98975			2.0E+05	2.1								
**270**	Guvacine	[M+K]+	C6H9NO2	166.02649	8.9E+04	1.5										
**271**	Tetrazolyl-glycine	[M+Na]+	C3H5N5O2	166.03355	2.0E+05	-1.8							1.5E+05	-2.0	1.2E+05	-0.8
**272**	Methylguanine	[M+H]+	C6H7N5O	166.07178							9.9E+04	-1.8			1.2E+05	3.3
**273**	Phenylalanine	[M+H]+	C9H11NO2	166.08626	1.3E+06	1.1			1.7E+05	-2.7	9.3E+04	-1.4				
**274**	Coniine	[M+K]+	C8H17N	166.09968									2.6E+05	-2.5		
**275**	Azulene	[M+K]+	C10H8	167.02569			5.4E+05	0.4								
**276**	Methylmercaptopurine	[M+H]+	C6H6N4S	167.03793			1.6E+05	4.0								
**277**	Hydroxy-3-nitrosobenzamide	[M+H]+	C7H6N2O3	167.04512	1.7E+05	0.5										
**278**	Noradrenochrome o-semiquinone	[M+H]+	C8H8NO3	167.05769	1.4E+05	-3.4										
**279**	Lysine 1,6-lactam	[M+K]+	C6H12N2O	167.05808			3.5E+05	0.3							3.1E+05	2.4

#	Putative Annotation [a]	Ion	Formula	$m/z_{Theo.}$[b]	R2020[c]	ppm	W2020[c]	ppm	B2020[c]	ppm	R2021[c]	ppm	W2021[c]	ppm	B2021[c]	ppm
**280**	Caffeyl alcohol	[M+H]+	C9H10O3	167.07021					4.8E+06	0.4						
**281**	Guanidinobutanamide	[M+Na]+	C5H12N4O	167.09033	1.5E+05	-2.7										
**282**	Caprylic acid	[M+Na]+	C8H16O2	167.10425	8.7E+04	-1.4							1.8E+05	-4.2		
**283**	1-(3-Aminopropyl)-4-aminobutanal	[M+Na]+	C7H16N2O	167.11548	7.1E+04	-0.4										
**284**	Keto-glutaramic acid	[M+Na]+	C5H7NO4	168.02658							1.5E+05	0.9			9.8E+04	-3.0
**285**	Thioguanine	[M+H]+	C5H5N5S	168.03444							6.9E+04	-3.6				
**286**	Guanidinobutanal	[M+K]+	C5H11N3O	168.05397											6.9E+04	-3.6
**287**	Allysine	[M+Na]+	C6H11NO3	168.06337					2.2E+05	-1.5						
**288**	Dihydrophenylalanine	[M+H]+	C9H13NO2	168.10191	1.0E+05	-0.1										
**289**	Lysinamide	[M+Na]+	C6H15N3O	168.11130					3.9E+05	-3.4						
**290**	Sulfopyruvic acid	[M+H]+	C3H4O6S	168.98014	3.7E+05	1.6	3.6E+05	0.5					7.1E+04	-0.6		
**291**	Mevaldic acid	[M+Na]+	C6H10O4	169.04713	1.5E+05	0.5	2.7E+05	2.0			2.3E+05	-4.1			1.0E+05	-0.2
**292**	Vanillic acid	[M+H]+	C8H8O4	169.04928					3.3E+06	1.5						
**293**	Glutamine	[M+Na]+	C5H10N2O3	169.05836	1.3E+05	0.3										
**294**	Hydroxyisoheptanoic acid	[M+Na]+	C7H14O3	169.08312							8.1E+04	2.3	2.4E+05	-2.2		
**295**	Agmatine	[M+K]+	C5H14N4	169.08546											5.3E+04	-2.7
**296**	Trimethylaminoethylphosphonate	[M+H]+	C5H15NO3P	169.08546											5.3E+04	4.6
**297**	Lysine	[M+Na]+	C6H14N2O2	169.09491					2.9E+06	-1.0						
**298**	Geranic acid	[M+H]+	C10H16O2	169.12229							7.7E+04	0.1				
**299**	Cysteic acid	[M+H]+	C3H7NO5S	170.01141	1.2E+05	4.3					7.2E+04	2.1	1.0E+05	-4.1		
**300**	Methylindole	[M+K]+	C9H9N	170.03666	9.9E+04	-3.0										
**301**	Pyridoxine	[M+H]+	C8H11NO3	170.08117	5.5E+05	2.0										
**302**	Oxaluric acid	[M+K]+	C3H4N2O4	170.98108									6.6E+04	-4.8		
**303**	4-Methylthio-2-oxobutanoic acid	[M+Na]+	C5H8O3S	171.00864	7.6E+04	4.2					8.1E+04	2.0				
**304**	Asparagine	[M+K]+	C4H8N2O3	171.01665	9.6E+04	-2.9					9.8E+04	4.3	8.1E+04	0.7		
**305**	Citramalic acid	[M+Na]+	C5H8O5	171.02691			1.5E+05	-3.0								
**306**	Methylenediurea	[M+K]+	C3H8N4O2	171.02788	8.3E+04	-2.4										
**307**	Dicyclopentadiene	[M+K]+	C10H12	171.05690					2.3E+05	0.9						
**308**	Mevalonic acid	[M+Na]+	C6H12O4	171.06283			1.3E+05	-0.3								
**309**	Dihydroxyphenylethyleneglycol	[M+H]+	C8H10O4	171.06532											5.1E+04	-0.8
**310**	Furfural diethyl acetal	[M+H]+	C9H14O3	171.10109											8.0E+04	2.8
**311**	Citronellic acid	[M+H]+	C10H18O2	171.13796	1.0E+05	-3.5			1.9E+05	-1.9						
**312**	2-Undecanone	[M+H]+	C11H22O	171.17405							8.4E+04	1.7				
**313**	Aspartate	[M+K]+	C4H7NO4	172.00165							6.2E+04	-4.7				
**314**	Ureidoglycine	[M+K]+	C3H7N3O3	172.01190	6.5E+04	-3.1										
**315**	Phenylpropionaldoxim	[M+Na]+	C9H11NO	172.07385											2.4E+05	-3.3
**316**	6E-Octene-2,4-diynoic acid	[M+K]+	C8H6O2	173.00003											4.9E+04	-0.5
**317**	S,S-Dimethyl-beta-propiothetin	[M+K]+	C5H10O2S	173.00331	1.2E+05	0.1										
**318**	Benzenamine sulfate	[M+H]+	C6H6NO3S	173.01347									8.8E+04	3.7		
**319**	Cinnamyl alcohol	[M+K]+	C9H10O	173.03571					3.4E+05	3.6						

#	Putative Annotation [a]	Ion	Formula	$m/z_{Theo.}$ [b]	R2020[c]	ppm	W2020[c]	ppm	B2020[c]	ppm	R2021[c]	ppm	W2021[c]	ppm	B2021[c]	ppm
**320**	Ribose	[M+Na]+	C5H10O5	173.04134			2.2E+05	4.1					3.4E+05	-4.1		
**321**	(Hydroxyphosphinyl)pyruvate	[M+Na]+	C3H5O5P	174.97641									1.2E+05	1.6		
**322**	Sulfinoalanine	[M+Na]+	C3H6NO4S	174.99097	1.0E+06	-0.3					2.3E+05	-5.0				
**323**	Threonic acid	[M+K]+	C4H8O5	174.99995									7.9E+04	2.2		
**324**	Mercaptopurine	[M+Na]+	C5H4N4S	175.00478			1.2E+05	0.6								
**325**	N-Methylnicotinamide	[M+K]+	C7H8N2O	175.02732									1.6E+05	-2.8		
**326**	Arabitol	[M+Na]+	C5H12O5	175.05794			2.5E+05	-1.4								
**327**	Shikimic acid	[M+H]+	C7H10O5	175.05933									1.1E+05	4.4		
**328**	Suberic acid	[M+H]+	C8H14O4	175.09727							9.0E+04	-4.5				
**329**	N-Acetylornithine	[M+H]+	C7H14N2O3	175.10703			1.3E+05	3.9								
**330**	Dodecapentaenal	[M+H]+	C12H14O	175.11174	3.4E+06	-1.6										
**331**	Arginine	[M+H]+	C6H14N4O2	175.11895	1.4E+07	0.3	2.1E+05	0.1	1.2E+07	-0.2	1.6E+05	0.0			3.6E+05	-0.2
**332**	N-Acetylhistamine	[M+Na]+	C7H11N3O	176.07868					9.8E+04	4.3						
**333**	Citrulline	[M+H]+	C6H13N3O3	176.10312									5.8E+04	-0.9		
**334**	Debrisoquine	[M+H]+	C10H13N3	176.11902									1.2E+05	-4.5		
**335**	Urocanic acid	[M+K]+	C6H6N2O2	177.00693			5.7E+05	-4.8			6.0E+05	-4.1			1.5E+05	-3.4
**336**	Imidazole pyruvic acid	[M+Na]+	C6H6N2O3	177.02668			1.6E+05	2.2								
**337**	Tyrosol	[M+K]+	C8H10O2	177.03124	1.4E+05	3.3										
**338**	Vanillyl alcohol	[M+Na]+	C8H10O3	177.05296							9.6E+04	-4.2				
**339**	4-Methylumbelliferone	[M+H]+	C10H8O3	177.05462	3.0E+05	-2.0										
**340**	Allantoic acid	[M+H]+	C4H8N4O4	177.06101	1.7E+05	-2.9	3.2E+05	4.6					1.8E+05	4.1		
**341**	Nonadienal	[M+K]+	C9H14O	177.06762	2.5E+05	3.4							1.5E+05	-3.9		
**342**	Propylmalate	[M+H]+	C7H12O5	177.07575	1.3E+05	-3.7	1.1E+05	0.0								
**343**	Boschnialactone	[M+Na]+	C9H14O2	177.08869							4.6E+04	-0.5	1.8E+05	-4.9		
**344**	Cinnamyl acetate	[M+H]+	C11H12O2	177.09096					3.3E+06	0.3						
**345**	3-Aminopropanesulfonate	[M+K]+	C3H9NO3S	177.99347	3.4E+05	1.2										
**346**	Methylaminoethylphosphonate	[M+K]+	C3H10NO3P	178.00300			3.0E+05	-0.1								
**347**	Nitrocatechol	[M+Na]+	C6H5NO4	178.01163							4.4E+04	-3.1				
**348**	S-(1-propenyl)-L-cysteine sulfoxide	[M+H]+	C6H11NO3S	178.05324	6.4E+06	0.0			8.3E+04	1.1	8.5E+05	-0.8			5.9E+04	-1.0
**349**	N-Formyl-L-methionine	[M+H]+	C6H11NO3S	178.05324	6.4E+06	0.0			8.3E+04	1.1	8.5E+05	-0.8			5.9E+04	-1.0
**350**	Alliin	[M+H]+	C6H11NO3S	178.05324	6.4E+06	0.0			8.3E+04	1.1	8.5E+05	-0.8			5.9E+04	-1.0
**351**	Histidine	[M+Na]+	C6H9N3O2	178.05840			1.0E+05	1.7								
**352**	Phosphoglycolic acid	[M+Na]+	C2H5O6P	178.97138							8.1E+04	1.2	7.5E+04	-3.3		
**353**	Uracil 5-carbaldehyde	[M+K]+	C5H4N2O3	178.98535	1.4E+05	3.0										
**354**	Imidazolepropionic acid	[M+K]+	C6H8N2O2	179.02241							1.8E+05	-3.8	1.6E+05	-4.4		
**355**	Imidazolelactic acid	[M+Na]+	C6H8N2O3	179.04271	8.3E+04	1.2										
**356**	Octadienoic acid	[M+K]+	C8H12O2	179.04684			1.5E+05	0.3			9.8E+04	1.8				
**357**	Indoleacetonitrile	[M+Na]+	C10H8N2	179.05797	1.5E+05	-0.8										
**358**	5-oxo-7-octenoic acid	[M+Na]+	C8H12O3	179.06786	1.1E+05	1.5										
**359**	Ferulaldehyde	[M+H]+	C10H10O3	179.06964					4.2E+06	3.5						

#	Putative Annotation [a]	Ion	Formula	$m/z_{Theo.}$ [b]	R2020[c]	ppm	W2020[c]	ppm	B2020[c]	ppm	R2021[c]	ppm	W2021[c]	ppm	B2021[c]	ppm
**360**	2-aminomuconic acid semialdehyde	[M+K]+	C6H7NO3	180.00550			8.0E+04	1.4								
**361**	Isoxanthopterin	[M+H]+	C6H5N5O2	180.05230			1.0E+05	-3.9								
**362**	Glucosamine	[M+H]+	C6H13NO5	180.08688			1.0E+05	-1.3								
**363**	Homophenylalanine	[M+H]+	C10H13NO2	180.10212					3.3E+05	-1.2	1.4E+05	0.7	1.6E+05	-2.3	1.7E+05	3.1
**364**	Hydroxymethyluracil	[M+K]+	C5H6N2O3	181.00100	4.4E+05	-2.7										
**365**	Isopropylmaleate	[M+Na]+	C7H10O4	181.04626							2.0E+06	4.8	2.3E+06	1.3	1.6E+06	4.9
**366**	Caffeic acid	[M+H]+	C9H8O4	181.04954	2.8E+05	0.6			1.4E+06	2.8						
**367**	Coniferyl alcohol	[M+H]+	C10H12O3	181.08538					7.4E+06	3.0						
**368**	3-tert-Butyl-5-methylcatechol	[M+H]+	C11H16O2	181.12231	8.5E+04	2.0										
**369**	4-Methyl-5-(2-hydroxyethyl)-thiazole	[M+K]+	C6H9NOS	182.00361					2.0E+05	0.2						
**370**	Tet-glycine	[M+K]+	C3H5N5O2	182.00807			8.2E+04	-3.2								
**371**	4-Methylene-L-glutamate	[M+Na]+	C6H9NO4	182.04246									3.9E+05	-0.5	2.8E+05	1.2
**372**	Valerylglycine	[M+Na]+	C7H13NO3	182.07902					2.0E+05	-1.4						
**373**	Tyrosine	[M+H]+	C9H11NO3	182.08117	7.6E+05	-0.2										
**374**	Dicyclohexylamine	[M+H]+	C12H23N	182.19053							1.9E+05	-1.1	1.1E+05	0.8		
**375**	Oxoadipic acid	[M+Na]+	C6H8O5	183.02585							6.2E+04	3.0				
**376**	aceto valeric acid	[M+K]+	C7H12O3	183.04239							7.3E+04	-3.2			8.1E+04	-4.0
**377**	Pimelic acid	[M+Na]+	C7H12O4	183.06241			3.0E+05	2.0					4.6E+05	-1.1		
**378**	Dihydrocaffeic acid	[M+H]+	C9H10O4	183.06421					5.5E+06	0.3						
**379**	5-Oxo-1,2-campholide	[M+H]+	C10H14O3	183.10185							1.5E+05	-1.5				
**380**	Butyro-betaine	[M+K]+	C8H18NO	183.10200	8.0E+04	0.1	1.5E+05	-3.3	4.2E+06	-3.4					6.8E+04	-4.3
**381**	Dihydrostilbene	[M+H]+	C14H14	183.11683	8.3E+04	-4.5										
**382**	Keto-glutaramic acid	[M+K]+	C5H7NO4	183.99987											4.6E+04	4.3
**383**	4-Amino-2-hydroxylamino-6-nitrotoluene	[M+H]+	C7H9N3O3	184.07130			8.6E+04	2.0								
**384**	Carnitinamide	[M+Na]+	C7H17N2O2	184.11822	1.2E+05	3.1	1.5E+05	-0.1								
**385**	Carnitinamide	[M+Na]+	C7H17N2O2	184.11824												
**386**	4-Hydroxy-2-oxoglutarate	[M+Na]+	C5H6O6	185.00566	1.8E+06	4.1										
**387**	Allicin	[M+Na]+	C6H10OS2	185.00653	1.8E+06	-0.6			1.6E+06	0.4	1.6E+05	1.5			1.6E+06	0.3
**388**	Thiouric acid	[M+H]+	C5H4N4O2S	185.01259											6.0E+04	1.0
**389**	Methyleneoxindole	[M+K40]+	C9H7NO	185.01546							6.5E+04	4.1				
**390**	Hydroxycoumarin	[M+Na]+	C9H6O3	185.02077			1.1E+05	0.8								
**391**	Hydroxyisourate	[M+H]+	C5H4N4O4	185.03114							2.2E+05	-3.3			1.4E+05	-1.1
**392**	Glutamine	[M+K]+	C5H10N2O3	185.03194									2.8E+05	2.0		
**393**	Hydroxymethyl-glutaric acid	[M+Na]+	C6H10O5	185.04181			9.6E+04	1.3								
**394**	3-O-Methylgallate	[M+H]+	C8H8O5	185.04522									1.4E+05	-4.2		
**395**	Oleandrose	[M+Na]+	C7H14O4	185.07880			1.2E+05	-2.0								
**396**	Oxo-decenoic acid	[M+H]+	C10H16O3	185.11748					4.9E+05	-1.4						
**397**	Citronellyl formate	[M+H]+	C11H20O2	185.15428			9.8E+04	-3.6	2.0E+05	-4.0						
**398**	Laurylaldehyde	[M+H]+	C12H24O	185.19018											7.2E+04	-1.0
**399**	Fucosamine	[M+Na]+	C6H13NO4	186.07398											7.0E+04	-1.6

#	Putative Annotation [a]	Ion	Formula	m/$z_{Theo.}$ [b]	R2020 [c]	ppm	W2020 [c]	ppm	B2020 [c]	ppm	R2021 [c]	ppm	W2021 [c]	ppm	B2021 [c]	ppm
**400**	N-Acetylphenylethylamine	[M+Na]+	C10H13NO	186.08967									1.5E+05	-4.0		
**401**	Sulfobenzaldehyde	[M+H]+	C7H6O4S	187.00683					2.9E+05	-4.7						
**402**	O-Carbamoyl-L-serine	[M+K]+	C4H8N2O4	187.01157	9.9E+04	1.1										
**403**	Cinnamic acid	[M+K]+	C9H8O2	187.01601					9.3E+04	-2.3						
**404**	Angelicin	[M+H]+	C11H6O3	187.03897	1.0E+05	2.9										
**405**	Quercitol	[M+Na]+	C6H12O5	187.05825			1.4E+05	-3.0					2.4E+05	-0.7		
**406**	p-Cumate	[M+Na]+	C10H12O2	187.07292									1.2E+05	0.2		
**407**	Carboxycyclohexyl-acetic acid	[M+H]+	C9H14O4	187.09621									8.2E+04	1.5		
**408**	Oxo-decanoic acid	[M+H]+	C10H18O3	187.13358											4.5E+04	-3.8
**409**	Pyridoxolactone	[M+Na]+	C8H7NO3	188.03181	1.5E+05	0.6										
**410**	Methyladenine	[M+K]+	C6H7N5	188.03384			9.8E+04	-2.8								
**411**	L-Methionine S-oxide	[M+Na]+	C5H11NO3S	188.03440									9.6E+04	4.2		
**412**	Indoleacrylic acid	[M+H]+	C11H9NO2	188.07060	3.7E+05	0.9										
**413**	N-Heptanoylglycine	[M+H]+	C9H17NO3	188.12725			1.8E+05	4.6								
**414**	Tartaric acid	[M+K]+	C4H6O6	188.97889							4.7E+04	3.7				
**415**	Phenylglyoxylic acid	[M+K]+	C8H6O3	188.99514											4.7E+04	-1.5
**416**	Ribose	[M+K]+	C5H10O5	189.01598	3.4E+05	1.5										
**417**	Dipropyl disulfide	[M+K]+	C6H14S2	189.01685	3.4E+05	-3.1										
**418**	Hydrocinnamic acid	[M+K]+	C9H10O2	189.03097					1.3E+05	1.4						
**419**	Caffeyl alcohol	[M+Na]+	C9H10O3	189.05268									3.2E+05	-2.5		
**420**	Ramentaceone	[M+H]+	C11H8O3	189.05531							4.2E+04	-3.7				
**421**	2-tridecene-4,7-diynal	[M+H]+	C13H16O	189.12685									6.1E+04	2.9		
**422**	Homoarginine	[M+H]+	C7H16N4O2	189.13460	1.8E+05	4.5										
**423**	Guanine	[M+K]+	C5H5N5O	190.01257	9.9E+04	0.7										
**424**	2-Dimethylamino-5,6-dimethylpyrimidin-4-ol	[M+Na]+	C8H13N3O	190.09516					8.2E+04	-0.4						
**425**	3-Mercapto-2-mercaptomethylpropanoate	[M+K]+	C4H8O2S2	190.95892							5.9E+04	4.3	1.2E+05	-3.7		
**426**	Phosphoenolpyruvic acid	[M+Na]+	C3H5O6P	190.97160	1.1E+05	-2.0					4.5E+04	-4.7				
**427**	Butanoylphosphate	[M+Na]+	C4H9O5P	191.00815											4.7E+04	-0.9
**428**	N1-Methyl-2-pyridone-5-carboxamide	[M+K]+	C7H8N2O2	191.02261							4.2E+05	-4.6				
**429**	Arabitol	[M+K]+	C5H12O5	191.03256			2.2E+05	-4.9								
**430**	Dehydroquinic acid	[M+H]+	C7H10O6	191.05556			1.2E+05	-2.9								
**431**	Ethoxycoumarin	[M+H]+	C11H10O3	191.06940					4.8E+06	4.6						
**432**	Chrysanthemic acid	[M+Na]+	C10H16O2	191.10389	3.8E+05	-2.0	3.6E+05	1.9			9.9E+04	-0.5	2.4E+05	-0.9		
**433**	Hydroxydebrisoquine	[M+H]+	C10H13N3O	192.11256			9.1E+04	3.0								
**434**	N-Butyl-1H-pyrazolo[3,4-d]pyrimidin-4-amine	[M+H]+	C9H13N5	192.12426									5.0E+04	0.6		
**435**	Diethylphosphoric acid	[M+K]+	C4H11O4P	193.00265	3.4E+05	2.3										
**436**	Thiourocanic acid	[M+Na]+	C6H6N2O2S	193.00456											2.1E+05	-1.8
**437**	Furfural diethyl acetal	[M+Na]+	C9H14O3	193.08352	1.2E+05	4.2									4.7E+04	0.2
**438**	Myristicin	[M+H]+	C11H12O3	193.08494			2.1E+05	0.9	1.3E+07	4.7						
**439**	Citronellic acid	[M+Na]+	C10H18O2	193.11990	1.2E+05	4.7					8.6E+04	4.9	2.5E+05	3.3		

#	Putative Annotation [a]	Ion	Formula	$m/z_{Theo.}$ [b]	R2020[c]	ppm	W2020[c]	ppm	B2020[c]	ppm	R2021[c]	ppm	W2021[c]	ppm	B2021[c]	ppm
**440**	2,4,7-tridecatrienal	[M+H]+	C13H20O	193.15849									9.4E+04	1.0		
**441**	Salsoline	[M+H]+	C11H15NO2	194.11763											2.0E+05	-0.4
**442**	Glucuronic acid	[M+H]+	C6H10O7	195.04993	7.5E+04	1.0										
**443**	Ferulic acid	[M+H]+	C10H10O4	195.06519	1.9E+05	4.3	2.9E+05	3.6	2.7E+06	3.9			4.9E+05	3.5		
**444**	4-n-valeryl butyric acid	[M+Na]+	C9H16O3	195.09954									6.2E+05	-1.9	1.5E+05	0.6
**445**	Zingerone	[M+H]+	C11H14O3	195.10157	6.3E+04	2.1			6.5E+06	1.6						
**446**	Aminomuconic acid	[M+K]+	C6H7NO4	196.00078											3.9E+04	-0.6
**447**	Orthanilic acid	[M+Na]+	C6H7NO3S	196.00388	3.7E+05	-2.1	9.7E+05	4.5								
**448**	Deoxy-5-aminoshikimic acid	[M+Na]+	C7H11NO4	196.05787									7.7E+04	0.8		
**449**	Glucosaminic acid	[M+H]+	C6H13NO6	196.08237											4.0E+04	-4.1
**450**	N-Acetyl-L-leucine	[M+Na]+	C8H15NO3	196.09453					2.1E+05	-0.6					8.1E+04	-0.4
**451**	Tyrosine methyl ester	[M+H]+	C10H13NO3	196.09633							6.1E+04	2.5				
**452**	Phenolic phosphate	[M+Na]+	C6H7O4P	196.99677									8.6E+04	3.3		
**453**	Allantoin	[M+K]+	C4H6N4O3	197.00773			9.2E+04	-2.9								
**454**	1-Naphthalenemethanol	[M+K]+	C11H10O	197.03620							6.3E+04	0.6				
**455**	N-Acetylasparagine	[M+Na]+	C6H10N2O4	197.05272											4.0E+04	2.8
**456**	Suberic acid	[M+Na]+	C8H14O4	197.07843	1.8E+05	0.8	3.6E+05	-0.3			2.2E+05	0.8	4.9E+05	-2.3	1.1E+05	0.6
**457**	Xanthoxylin	[M+H]+	C10H12O4	197.08010					6.2E+06	3.7						
**458**	N-Acetylornithine	[M+Na]+	C7H14N2O3	197.08883											6.9E+04	4.2
**459**	Hydroxy pelargonic acid	[M+Na]+	C9H18O3	197.11482	2.4E+05	3.4	2.6E+05	-0.5					1.6E+06	-1.6	2.5E+05	-0.3
**460**	Coryneine	[M+H]+	C11H18NO2	197.14103	9.3E+04	-3.0										
**461**	Indole-3-acetaldehyde	[M+K]+	C10H9NO	198.03093									5.9E+04	3.3		
**462**	Dihydroxy-L-phenylalanine	[M+H]+	C9H11NO4	198.07608	6.6E+04	0.3										
**463**	Citrulline	[M+Na]+	C6H13N3O3	198.08491	6.6E+04	2.8										
**464**	N-Formimino-L-aspartate	[M+K]+	C5H8N2O4	199.01157	9.0E+04	-0.1	4.2E+05	0.4			1.7E+05	-1.4	1.1E+05	-2.5	8.3E+04	-0.8
**465**	Ascorbic acid	[M+Na]+	C6H8O6	199.02174							6.1E+04	-2.2			4.6E+04	-2.9
**466**	Vanillylmandelic acid	[M+H]+	C9H10O5	199.05932					8.2E+05	3.9						
**467**	Dehydrocarnitine	[M+K]+	C7H14NO3	199.06086							4.8E+04	-1.7				
**468**	5-Acetylamino-6-amino-3-methyluracil	[M+H]+	C7H10N4O3	199.08188											4.8E+04	3.4
**469**	Dihydroxy-octanoic acid	[M+Na]+	C8H16O4	199.09440			2.1E+05	-1.6			9.1E+04	3.9	2.0E+05	0.5		
**470**	Trimethyl-L-histidine	[M+H]+	C9H16N3O2	199.13143					2.5E+05	0.5						
**471**	Linderic acid	[M+H]+	C12H22O2	199.16921							9.9E+04	0.2	9.8E+04	-4.0		
**472**	Alliin	[M+Na]+	C6H11NO3S	200.03518	9.4E+05	0.4										
**473**	S-(1-propenyl)-L-cysteine sulfoxide	[M+Na]+	C6H11NO3S	200.03518	9.4E+05	0.4										
**474**	N-Formyl-L-methionine	[M+Na]+	C6H11NO3S	200.03518	9.4E+05	0.4										
**475**	Methylthiohexanaldoxime	[M+K]+	C7H15NOS	200.05115					8.7E+04	-2.8						
**476**	Dodecanamide	[M+H]+	C12H25NO	200.20084											3.9E+04	0.3
**477**	Umbelliferone	[M+K]+	C9H6O3	200.99554			1.2E+05	-3.4								
**478**	Cysteinylglycine	[M+Na]+	C5H10N2O3S	201.03043	8.7E+04	-4.4										
**479**	Safrole	[M+K]+	C10H10O2	201.03129					2.0E+05	-0.3						

#	Putative Annotation [a]	Ion	Formula	$m/z_{Theo.}$ [b]	R2020[c]	ppm	W2020[c]	ppm	B2020[c]	ppm	R2021[c]	ppm	W2021[c]	ppm	B2021[c]	ppm
**480**	Hydroxy-L-lysine	[M+K]+	C6H14N2O3	201.06406							5.7E+04	-2.3				
**481**	Nicotine	[M+K]+	C10H14N2	201.07851											5.1E+04	1.7
**482**	Eugenol methyl ether	[M+Na]+	C11H14O2	201.08860	3.7E+05	-3.0	1.6E+05	3.9	1.2E+05	3.9						
**483**	Isoxanthopterin	[M+Na]+	C6H5N5O2	202.03355	5.9E+04	-1.7					5.2E+04	3.8				
**484**	Amino-undecanoic acid	[M+H]+	C11H23NO2	202.17940											3.9E+04	3.7
**485**	5-Hydroxy-2-oxo-4-ureido-2,5-dihydro-1H-imidazole-5-carboxylate	[M+H]+	C5H6N4O5	203.04120							9.8E+04	-0.5				
**486**	Glucose	[M+Na]+	C6H12O6	203.05261	1.7E+06	-1.4	4.0E+06	0.2					5.6E+06	0.0		
**487**	alpha-Curcumene	[M+H]+	C15H22	203.17943	1.1E+05	1.8										
**488**	Methionine sulfoxide	[M+K]+	C5H11NO3S	204.00916					7.8E+04	-0.2						
**489**	2-Methyl-3-hydroxy-5-formylpyridine-4-carboxylate	[M+Na]+	C8H7NO4	204.02663											3.8E+04	0.5
**490**	Acetylcarnitine	[M+H]+	C9H17NO4	204.12359					1.5E+05	-2.7	4.4E+04	0.5				
**491**	Succimer	[M+Na]+	C4H6O4S2	204.95927			9.4E+04	3.4								
**492**	Mannitol	[M+Na]+	C6H14O6	205.06814			3.9E+05	0.6					1.8E+05	1.5		
**493**	Dihydroconiferyl alcohol	[M+Na]+	C10H14O3	205.08348											4.0E+04	0.2
**494**	3-Butylidene-7-hydroxyphthalide	[M+H]+	C12H12O3	205.08531					1.1E+06	3.0						
**495**	Tryptophan	[M+H]+	C11H12N2O2	205.09715	5.7E+05	2.9										
**496**	Diethyl-2-methyl-3-hydroxysuccinate	[M+H]+	C9H16O5	205.10637			9.0E+04	3.3								
**497**	Isocaryophyllene	[M+H]+	C15H24	205.19510			8.8E+04	-0.1	4.5E+05	-4.6						
**498**	Homocysteinesulfinic acid	[M+K]+	C4H9NO4S	205.98839	8.9E+04	2.5										
**499**	Nalpha,Nalpha-Dimethyl-histidine	[M+Na]+	C8H13N3O2	206.08927					2.4E+05	3.5						
**500**	2-Acetamido-2,6-dideoxy-D-galactose	[M+H]+	C8H15NO5	206.10230	8.1E+04	-2.1										
**501**	Pantothenol	[M+H]+	C9H19NO4	206.13801			8.2E+04	3.3								
**502**	Diaminosalicylic acid	[M+K]+	C7H8N2O3	207.01665	1.1E+06	4.3					1.8E+06	-4.6				
**503**	N-Cyclopropylammelide	[M+K]+	C6H8N4O2	207.02788	2.5E+05	2.6										
**504**	Homocitric acid	[M+H]+	C7H10O7	207.05035			1.2E+05	-2.0								
**505**	Lipoic acid	[M+H]+	C8H14O2S2	207.05035			1.2E+05	2.2								
**506**	Phosphorylcholine	[M+Na]+	C5H15NO4P	207.06320									1.7E+05	-0.5		
**507**	Geranic acid	[M+K]+	C10H16O2	207.07819	2.3E+05	0.0										
**508**	Withasomnine	[M+Na]+	C12H12N2	207.08864											6.5E+04	3.0
**509**	5-exo-Hydroxy-1,2-campholide	[M+Na]+	C10H16O3	207.09882			1.6E+05	1.7					2.3E+05	2.5	4.7E+04	2.7
**510**	Methoxycinnamic acid ethyl ester	[M+H]+	C12H14O3	207.10150					2.6E+06	0.3						
**511**	Citronellyl formate	[M+Na]+	C11H20O2	207.13555									1.6E+05	0.0		
**512**	11-dodecen-1-ol	[M+Na]+	C12H24O	207.17258			1.2E+05	-3.1								
**513**	Octylphenol	[M+H]+	C14H22O	207.17434	9.8E+04	0.3										
**514**	2-Amino-3-carboxymuconate semialdehyde	[M+Na]+	C7H7NO5	208.02153							9.2E+04	0.5				
**515**	2-Deoxy-2-dimethylamino-alpha-D-Glucose	[M+H]+	C8H17NO5	208.11756							4.5E+04	1.9				
**516**	Phospho-D-glycerate	[M+Na]+	C3H7O7P	208.98136							1.7E+05	3.8				
**517**	Fraxetin	[M+H]+	C10H8O5	209.04500			1.1E+05	-2.6			2.7E+05	1.3	2.4E+05	-0.5	1.7E+05	1.4

#	Putative Annotation [a]	Ion	Formula	$m/z_{Theo.}$ [b]	R2020[c]	ppm	W2020[c]	ppm	B2020[c]	ppm	R2021[c]	ppm	W2021[c]	ppm	B2021[c]	ppm
**518**	cis-2-Carboxycyclohexyl-acetic acid	[M+Na]+	C9H14O4	209.07793									3.1E+05	2.4		
**519**	Benzylsuccinate	[M+H]+	C11H12O4	209.08009					9.7E+06	3.6						
**520**	Oxodecanoate	[M+Na]+	C10H18O3	209.11481									3.8E+05	0.0	1.0E+05	1.4
**521**	Benzyl -2-methyl-3-hydroxybutanoate	[M+H]+	C12H16O3	209.11706			1.3E+05	0.8								
**522**	Methyl-decanoic acid	[M+Na]+	C11H22O2	209.15208			1.7E+05	-4.2								
**523**	Heptyloxyphenol	[M+H]+	C13H20O2	209.15316							1.8E+05	2.1			9.6E+04	4.1
**524**	Ammodendrine	[M+H]+	C12H20N2O	209.16484	9.5E+04	0.0										
**525**	2-(Acetamidomethylene)succinate	[M+Na]+	C7H9NO5	210.03721							7.3E+04	0.4				
**526**	Hydroxyiminostilbene	[M+H]+	C14H11NO	210.09099											4.7E+04	1.7
**527**	8-Amino-7-oxononanoic acid	[M+Na]+	C9H17NO3	210.10935			6.0E+05	3.4								
**528**	Decanamide	[M+K]+	C10H21NO	210.12611									4.8E+04	-3.0		
**529**	Hydantoin-propionate	[M+K]+	C6H8N2O4	211.01157	8.0E+04	1.2					3.5E+05	-0.4	2.5E+05	-2.8	3.5E+05	-0.7
**530**	Glucaric acid	[M+H]+	C6H10O8	211.04484	9.1E+04	2.2										
**531**	Glycylproline	[M+K]+	C7H12N2O3	211.04894							1.6E+05	-4.7				
**532**	Sedoheptulose	[M+H]+	C7H14O7	211.08185									1.5E+05	-2.9		
**533**	1,3,7-Trimethyluric acid	[M+H]+	C8H10N4O3	211.08312							8.2E+04	-2.6				
**534**	Azelaic acid	[M+Na]+	C9H16O4	211.09408	1.5E+05	-1.6	4.1E+05	-0.3			2.4E+05	-1.2	5.8E+05	-0.8	1.6E+05	-1.3
**535**	Acetylagmatine	[M+K]+	C7H16N4O	211.09569					5.2E+06	-0.6						
**536**	Jasmonic acid	[M+H]+	C12H18O3	211.13220									2.0E+05	3.2		
**537**	Undecanol	[M+K]+	C11H24O	211.14578									7.8E+04	0.5		
**538**	Aminobenzenesulfonate	[M+K]+	C6H7NO3S	211.97722											4.9E+04	2.8
**539**	Quinaldinic acid	[M+K]+	C10H7NO2	212.01102											7.2E+04	-0.9
**540**	N-Acetyl-L-glutamate	[M+Na]+	C7H11NO5	212.05294							4.8E+04	0.0				
**541**	Menadiol	[M+K]+	C11H10O2	213.03169									6.4E+04	-2.1		
**542**	N-Carbamyl-L-glutamate	[M+Na]+	C6H10N2O5	213.04914											5.7E+04	-4.4
**543**	N-Acetylornithine	[M+K]+	C7H14N2O3	213.06450							6.2E+04	-4.2			1.7E+05	-1.7
**544**	Arginine	[M+K]+	C6H14N4O2	213.07484	2.0E+05	0.4							1.1E+05	2.1		
**545**	Propyl gallate	[M+H]+	C10H12O5	213.07543					9.3E+05	1.5						
**546**	Arginine	[M+K]+	C6H14N4O2	213.07576											7.8E+04	-4.3
**547**	Pinosylvin	[M+H]+	C14H12O2	213.09101	9.7E+04	-3.6										
**548**	Perseitol	[M+H]+	C7H16O7	213.09696									6.1E+04	-0.4		
**549**	p-Cumylphenol	[M+H]+	C15H16O	213.12697							4.0E+04	2.0				
**550**	Citric acid	[M+Na]+	C6H8O7	215.01622	2.4E+05	-0.4										
**551**	Dinitroguaiacol	[M+H]+	C7H6N2O6	215.03066									8.2E+04	-3.7		
**552**	2-Deoxy-D-ribose 1-phosphate	[M+H]+	C5H11O7P	215.03066									8.2E+04	4.0		
**553**	Amino-3-(3-carboxy-5-methyl-4-isoxazolyl)porionic acid	[M+H]+	C8H10N2O5	215.06656									9.0E+04	-1.5		
**554**	Methyl 2-diazoacetamidohexonate	[M+H]+	C9H16N3O3	215.12644	1.9E+05	-1.3							2.4E+05	3.8	2.3E+05	4.6
**555**	N-Formyl-L-methionine	[M+K]+	C6H11NO3S	216.00912	1.2E+06	2.0										
**556**	Alliin	[M+K]+	C6H11NO3S	216.00912	1.2E+06	2.0										

#	Putative Annotation [a]	Ion	Formula	$m/z_{Theo.}$ [b]	R2020 [c]	ppm	W2020 [c]	ppm	B2020 [c]	ppm	R2021 [c]	ppm	W2021 [c]	ppm	B2021 [c]	ppm
**557**	S-(1-propenyl)-L-cysteine sulfoxide	[M+K]+	C6H11NO3S	216.00912	1.2E+06	2.0										
**558**	Methyl beta-D-galactoside	[M+Na]+	C7H14O6	217.06840			1.3E+05	-0.7								
**559**	Hydroxynicotine	[M+K]+	C10H14N2O	217.07377	1.4E+05	0.5										
**560**	Tetradecadiene-diynoic acid	[M+H]+	C14H16O2	217.12321					8.2E+05	-4.2						
**561**	Undecanedioic acid	[M+H]+	C11H20O4	217.14346							7.3E+04	-0.1				
**562**	Isoxanthopterin	[M+K]+	C6H5N5O2	218.00748	1.4E+05	-1.5										
**563**	Cyclohexylsulfamate	[M+K]+	C6H13NO3S	218.02388					1.2E+05	4.1						
**564**	Pyridarone	[M+Na]+	C13H9NO	218.05783											3.7E+04	-0.9
**565**	L-Homophenylalanine	[M+K]+	C10H13NO2	218.05783											3.7E+04	-0.2
**566**	2-(Hydroxymethyl)-3-(acetamidomethylene)succinate	[M+H]+	C8H11NO6	218.06677									7.7E+04	-3.9		
**567**	Tyrosine methyl ester	[M+Na]+	C10H13NO3	218.07920									1.7E+05	-2.0		
**568**	3-Methylindolepyruvate	[M+H]+	C12H11NO3	218.08101											3.9E+04	0.7
**569**	Sulfosalicylic acid	[M+H]+	C7H6O6S	218.99632					1.5E+05	-2.4						
**570**	Caffeic acid	[M+K]+	C9H8O4	219.00542	3.1E+05	1.7									4.0E+04	4.2
**571**	Isonicotinylglycine	[M+K]+	C8H8N2O3	219.01583							1.0E+05	3.8				
**572**	Glucose	[M+K]+	C6H12O6	219.02655	1.4E+06	4.3	2.2E+06	-0.4					5.2E+05	-0.6		
**573**	Aminophenylalanine	[M+K]+	C9H12N2O2	219.05372									1.4E+05	-3.1		
**574**	1,2,3-triacetyl-glycerol	[M+H]+	C9H14O6	219.08556							5.0E+04	3.4				
**575**	5-L-Glutamyl-L-alanine	[M+H]+	C8H14N2O5	219.09670											7.0E+04	3.9
**576**	Geranyl acetate	[M+Na]+	C12H20O2	219.13470									1.6E+05	3.9		
**577**	Tetradecapentaenoic acid	[M+H]+	C14H18O2	219.13733							8.0E+04	2.9				
**578**	Guanidinoethyl methyl phosphate	[M+Na]+	C4H12N3O4P	220.04626							4.4E+04	-2.3				
**579**	N-Hydroxy-L-tyrosine	[M+Na]+	C9H11NO4	220.05863							3.6E+04	-2.7				
**580**	Amidino-3-keto-scyllo-inosamine	[M+H]+	C7H13N3O5	220.09348											4.0E+04	-3.1
**581**	Succimer	[M+K]+	C4H6O4S2	220.93457									9.2E+05	-3.0		
**582**	Dihydrocaffeic acid	[M+K]+	C9H10O4	221.02107	2.7E+05	1.9	2.2E+05	-3.5					1.7E+05	1.5		
**583**	Imidazole acetol-phosphate	[M+H]+	C6H9N2O5P	221.03218	7.5E+05	3.1	3.2E+05	-2.3					3.7E+05	-4.1		
**584**	Diisopropyl phosphate	[M+K]+	C6H15O4P	221.03340											1.9E+05	2.5
**585**	Purpurogallin	[M+H]+	C11H8O5	221.04445	3.5E+05	2.0										
**586**	N-Nitrosodiphenylamine	[M+Na]+	C12H10N2O	221.06758											7.0E+04	4.3
**587**	cis-2,3-Dihydroxy-2,3-dihydro-p-cumate	[M+Na]+	C10H14O4	221.07891											3.6E+04	-2.2
**588**	N-Acetyl-beta-D-glucosaminylamine	[M+H]+	C8H16N2O5	221.11348									1.3E+05	-1.3		
**589**	Linderic acid	[M+Na]+	C12H22O2	221.15120	1.7E+05	2.7	1.9E+05	-3.4								
**590**	Caryophyllene epoxide	[M+H]+	C15H24O	221.19020					5.2E+05	-0.9						
**591**	p-Hydroxyphenylacetothiohydroximate	[M+K]+	C8H9NO2S	221.99879							4.2E+04	-1.0				
**592**	Pyridoxic acid	[M+K]+	C8H9NO4	222.01680			7.8E+04	-2.2								
**593**	Dictamnine	[M+Na]+	C12H9NO2	222.05323							4.6E+04	-3.1				
**594**	3-(3,4-Dihydroxypyridin-1-yl)-L-alanine	[M+Na]+	C8H11N2O4	222.06087					1.2E+05	1.0						
**595**	Nalpha,Nalpha-Dimethyl-L-histidine	[M+K]+	C8H13N3O2	222.06397							3.6E+04	-0.2				

#	Putative Annotation [a]	Ion	Formula	$m/z_{Theo.}$ [b]	R2020[c]	ppm	W2020[c]	ppm	B2020[c]	ppm	R2021[c]	ppm	W2021[c]	ppm	B2021[c]	ppm
**596**	Erythrose 4-phosphate	[M+Na]+	C4H9O7P	222.99879									6.7E+04	-4.4		
**597**	3-(3'-Methylthio)propylmalic acid	[M+H]+	C8H14O5S	223.06347	1.0E+05	-3.9										
**598**	Decenedioic acid	[M+Na]+	C10H16O4	223.09408	1.4E+05	-2.8							7.4E+05	-0.9	2.4E+05	-2.4
**599**	Apiole	[M+H]+	C12H14O4	223.09572			5.6E+05	3.4	7.4E+06	3.4	3.7E+05	2.0				
**600**	Lauric acid	[M+Na]+	C12H24O2	223.16685	6.1E+04	3.7										
**601**	Capryloylglycine	[M+Na]+	C10H19NO3	224.12669											4.0E+04	-4.4
**602**	Sulfobenzaldehyde	[M+K]+	C7H6O4S	224.96247					2.1E+05	-2.8						
**603**	Bergaptol	[M+Na]+	C11H6O4	225.01539							1.6E+05	2.0				
**604**	Dimethoxy-4-hydroxycinnamic acid	[M+H]+	C11H12O5	225.07671											1.3E+05	-4.3
**605**	3-Hydroxykynurenine	[M+H]+	C10H12N2O4	225.08643							9.6E+04	2.5				
**606**	Sebacic acid	[M+Na]+	C10H18O4	225.10973	1.2E+05	2.4	2.8E+05	-3.7			1.9E+05	-2.6	5.4E+05	-0.1		
**607**	2-Amino-1-methyl-6-phenylimidazo[4,5-b]pyridine	[M+H]+	C13H12N4	225.11253					3.7E+06	4.2						
**608**	4-Amino-4-deoxychorismate;ADC	[M+H]+	C10H11NO5	226.07028							3.9E+04	3.2				
**609**	N-Heptanoylglycine	[M+K]+	C9H17NO3	226.08416			1.0E+05	-0.7								
**610**	L-beta-aspartyl-L-alanine	[M+Na]+	C7H12N2O5	227.06477			1.0E+05	-4.1								
**611**	Dimethylenetriurea	[M+Na]+	C5H12N6O3	227.08681			1.5E+05	-2.2								
**612**	Genipin	[M+H]+	C11H14O5	227.09077					1.8E+06	2.8						
**613**	O-Acetylcarnitine	[M+Na]+	C9H18NO4	227.11221							5.9E+04	2.6				
**614**	Tuberonic acid	[M+H]+	C12H18O4	227.12779	2.5E+05	-2.4										
**615**	1,8-Diazacyclotetradecane-2,9-dione	[M+H]+	C12H22N2O2	227.17588											3.7E+04	-2.1
**616**	S-Prenyl-L-cysteine	[M+K]+	C8H15NO2S	228.04576											5.5E+04	-1.1
**617**	1-Methyl-4-phenyl-1,2,3,6-tetrahydropyridine N-oxide	[M+K]+	C12H15NO	228.07950					1.2E+05	-4.3						
**618**	Methylthiooctanaldoxime	[M+K]+	C9H19NOS	228.08174			7.9E+04	0.7					6.0E+04	-0.3		
**619**	Deoxycytidine	[M+H]+	C9H13N3O4	228.09693											4.5E+04	4.2
**620**	Oxalosuccinic acid	[M+K]+	C6H6O7	228.97532									4.8E+04	-3.5		
**621**	4-Nitroquinoline N-oxide	[M+K]+	C9H6N2O3	229.00041							5.1E+04	2.6				
**622**	Serine phosphoethanolamine	[M+H]+	C5H13N2O6P	229.05879					2.6E+05	-1.7						
**623**	Resveratrol	[M+H]+	C14H12O3	229.08655			1.4E+05	-2.7								
**624**	trans,trans-1,4-Diphenyl-1,3-butadiene	[M+Na]+	C16H14	229.09877	1.0E+05	-0.9										
**625**	Monoethylglycinexylidide	[M+Na]+	C12H18N2O	229.13128					1.7E+05	-0.6						
**626**	Dodecenedioic acid	[M+H]+	C12H20O4	229.14350											5.5E+04	-0.3
**627**	L-isoleucyl-L-proline	[M+H]+	C11H20N2O3	229.15515			8.8E+04	-2.1								
**628**	Octylphenol	[M+Na]+	C14H22O	229.15629	1.2E+05	2.0										
**629**	N-Acetyl-L-methionine	[M+K]+	C7H13NO3S	230.02431											3.7E+04	2.0
**630**	S-(2-Hydroxyethyl)-N-acetyl-L-cysteine	[M+Na]+	C7H13NO4S	230.04546					1.0E+05	1.3						
**631**	Dihydrolipoamide	[M+Na]+	C8H17NOS2	230.06377			1.4E+05	2.6								
**632**	Hydroxydebrisoquine	[M+K]+	C10H13N3O	230.06838									5.7E+04	2.8		
**633**	Anthocyanin	[M+Na]+	C15H11O	230.07021							5.4E+04	0.0				

#	Putative Annotation [a]	Ion	Formula	m/$z_{Theo.}$ [b]	R2020[c]	ppm	W2020[c]	ppm	B2020[c]	ppm	R2021[c]	ppm	W2021[c]	ppm	B2021[c]	ppm
**634**	N-Acetyl-D-phenylalanine	[M+Na]+	C11H13NO3	230.07810											5.6E+04	2.9
**635**	N-Butyl-1H-pyrazolo[3,4-d]pyrimidin-4-amine	[M+K]+	C9H13N5	230.08027									6.1E+04	-0.1		
**636**	Butenylcarnitine	[M+H]+	C11H19NO4	230.13938							1.8E+05	-3.0	1.9E+05	3.2	2.3E+05	-3.9
**637**	Decanoylglycine	[M+H]+	C12H23NO3	230.17507	3.4E+05	0.1									8.8E+04	-1.2
**638**	Xestoaminol C	[M+H]+	C14H31NO	230.24784	1.2E+05	-3.1					3.9E+05	-1.3	4.2E+05	1.3	1.2E+05	-1.1
**639**	Citric acid	[M+K]+	C6H8O7	230.99016	2.9E+05	-1.7										
**640**	Myristicin	[M+K]+	C11H12O3	231.04087					1.9E+05	4.0						
**641**	Chalcone	[M+Na]+	C15H12O	231.07834							9.1E+04	-1.3				
**642**	12-oxo-5E,8E,10Z-dodecatrienoic acid	[M+Na]+	C12H16O3	231.09917	1.6E+05	3.2										
**643**	Diphenylcyclobutane	[M+Na]+	C16H16	231.11442	9.7E+04	-1.1										
**644**	Linderenol	[M+H]+	C15H18O2	231.13724									1.4E+05	3.1		
**645**	Dodecanedioic acid	[M+H]+	C12H22O4	231.15878							4.0E+04	1.3				
**646**	Hydroxy-tridecanoic acid	[M+H]+	C13H26O3	231.19636					1.3E+05	-3.8						
**647**	Hydroxypseudooxynicotine	[M+K]+	C10H14N2O2	233.06801											6.4E+04	2.9
**648**	Dihydrochalcone	[M+Na]+	C15H14O	233.09416							9.2E+04	-2.0				
**649**	Aplotaxene	[M+H]+	C17H28	233.22686					2.5E+05	-2.1						
**650**	Hydroxypropionylcarnitine	[M+H]+	C10H19NO5	234.13401			1.1E+05	-1.8								
**651**	Deoxynupharidine	[M+H]+	C15H23NO	234.18470									9.6E+04	2.3		
**652**	(Homo)3-citrate	[M+H]+	C9H14O7	235.08202									8.7E+05	-3.4		
**653**	Harmine	[M+Na]+	C13H12N2O	235.08483							8.0E+05	-2.8				
**654**	Acetoxychavicol acetate	[M+H]+	C13H14O4	235.09678											3.4E+05	-1.3
**655**	3,7-Dimethyl-8,11-dioxo-2E,6E,9E-dodecatrienal	[M+H]+	C14H18O3	235.13261					3.5E+06	1.1						
**656**	2-methyl-2-dodecenoic acid	[M+Na]+	C13H24O2	235.16668			2.1E+05	0.7								
**657**	2-methyl-2-dodecenoic acid	[M+Na]+	C13H24O2	235.16685	1.5E+05	2.8										
**658**	Myristaldehyde	[M+Na]+	C14H28O	235.20403			2.4E+05	-3.4								
**659**	Hexadecatrienal	[M+H]+	C16H26O	235.20564	2.4E+05	-0.6										
**660**	Benzenedisulfonamide	[M+H]+	C6H8N2O4S2	236.99893					1.1E+06	3.8						
**661**	Capillarin	[M+K]+	C13H10O2	237.03124	1.4E+05	3.2										
**662**	Trimethoxycoumarin	[M+H]+	C12H12O5	237.07498			1.3E+06	3.2								
**663**	Trimethoxycoumarin	[M+H]+	C12H12O5	237.07575	2.3E+05	-0.2										
**664**	N-(Phenylmethyl)-N-methyl-2-pyridinamine	[M+K]+	C13H14N2	237.07828							6.0E+04	2.4			4.5E+04	4.2
**665**	Coryneine	[M+K41]+	C11H18NO2	237.09452											4.1E+04	2.2
**666**	Dimethylallyl-hydroxymandelic acid	[M+H]+	C13H16O4	237.11171					9.6E+06	1.8	1.5E+05	0.3				
**667**	Heptyloxybenzoic acid	[M+H]+	C14H20O3	237.14754											8.1E+04	4.1
**668**	Capsidiol	[M+H]+	C15H24O2	237.18476											4.6E+04	0.6
**669**	Hexadecynal	[M+H]+	C16H28O	237.22129	9.0E+04	-0.1										
**670**	N-Nonanoylglycine	[M+Na]+	C11H21NO3	238.14136	6.6E+04	1.6					6.1E+04	1.9				
**671**	Trimethylallantoin	[M+K]+	C7H12N4O3	239.05410	7.4E+04	3.7										
**672**	Decenedioic acid	[M+K]+	C10H16O4	239.06832			8.4E+04	-1.3								

#	Putative Annotation [a]	Ion	Formula	m/z$_{Theo.}$ [b]	R2020[c]	ppm	W2020[c]	ppm	B2020[c]	ppm	R2021[c]	ppm	W2021[c]	ppm	B2021[c]	ppm
**673**	Flavonol	[M+H]+	C15H10O3	239.07027	9.9E+04	2.9										
**674**	Trimethoxycinnamic acid	[M+H]+	C12H14O5	239.09061					6.1E+06	3.3						
**675**	Tetrahydrozoline	[M+K]+	C13H16N2	239.09451											1.0E+05	0.0
**676**	Methoxychalcone	[M+H]+	C16H14O2	239.10709					1.3E+06	-1.8						
**677**	7-hydroxy-dodecanoic acid	[M+Na]+	C12H24O3	239.16226											1.5E+05	-2.1
**678**	Hydroxy lauric acid	[M+Na]+	C12H24O3	239.16270									4.4E+05	-3.9		
**679**	Sundiversifolide	[M+H]+	C14H22O3	239.16417	1.2E+05	-2.5	1.9E+05	0.6			1.2E+05	-2.6				
**680**	2-(Acetamidomethylene)-3-(hydroxymethyl)succinate	[M+Na]+	C8H11NO6	240.04845							7.4E+04	-2.5				
**681**	Methylindolepyruvate	[M+Na]+	C12H11NO3	240.06313							6.4E+04	-0.1			3.6E+04	-1.9
**682**	beta-Alanyl-L-lysine	[M+Na]+	C9H19N3O3	240.13128					3.2E+05	2.4						
**683**	Amino-undecanoic acid	[M+K]+	C11H23NO2	240.13648											3.6E+04	-1.8
**684**	Bergaptol	[M+K]+	C11H6O4	240.99028											9.6E+04	-2.1
**685**	Dihydroxyanthraquinone	[M+H]+	C14H8O4	241.04923			9.4E+04	1.3								
**686**	Sebacic acid	[M+K]+	C10H18O4	241.08367	1.2E+05	-1.0										
**687**	Hydroxysebacic acid	[M+Na]+	C10H18O5	241.10515			9.9E+04	-2.1					1.6E+05	-2.9		
**688**	Dimethyl-L-arginine	[M+K]+	C8H18N4O2	241.10614	2.4E+05	-1.9			2.1E+06	-3.0						
**689**	O-Succinyl-L-homoserine	[M+Na]+	C8H13NO6	242.06448									5.3E+04	-4.0		
**690**	L-Acetylcarnitine	[M+K]+	C9H17NO4	242.07966									7.4E+04	-3.1		
**691**	N-Phenyl-1-naphthylamine	[M+Na]+	C16H13N	242.09370											4.9E+04	1.3
**692**	Pantothenic acid	[M+Na]+	C9H17NO5	242.10015			1.1E+05	-1.1								
**693**	Pentahomomethionine	[M+Na]+	C10H21NO2S	242.11783									5.9E+04	2.9		
**694**	Diethyl-2-methyl-3-hydroxysuccinate	[M+K]+	C9H16O5	243.06386									5.2E+04	-3.8		
**695**	gamma-Hydroxy-L-homoarginine	[M+K]+	C7H16N4O3	243.08463							4.8E+04	3.2				
**696**	Caulophylline	[M+K]+	C12H16N2O	243.08945					2.7E+05	-0.1						
**697**	Lipoamide	[M+K]+	C8H15NOS2	244.02347											6.6E+04	-3.3
**698**	Pantothenol	[M+K]+	C9H19NO4	244.09399									1.1E+05	2.4		
**699**	N-Undecanoylglycine	[M+H]+	C13H25NO3	244.19072	1.4E+05	-1.3	1.8E+05	-3.1								
**700**	2-amino-tetradecanoic acid	[M+H]+	C14H29NO2	244.22720									5.9E+04	-0.4		
**701**	Sulfolactone	[M+Na]+	C6H6O7S	244.97270							5.3E+04	-0.2				
**702**	6-Acetyl-D-glucose	[M+Na]+	C8H14O7	245.06332									3.3E+05	-0.6	8.8E+04	-1.8
**703**	Apiole	[M+Na]+	C12H14O4	245.07843	5.5E+05	3.4	7.1E+05	-2.0					1.2E+06	0.1	3.2E+05	-2.1
**704**	Piceatannol	[M+H]+	C14H12O4	245.08143					2.2E+05	-2.4	3.9E+05	1.7				
**705**	Osthol	[M+H]+	C15H16O3	245.11740					3.6E+05	-0.7						
**706**	Dodecatrienyl acetate	[M+Na]+	C14H22O2	245.15120	9.5E+04	-3.8										
**707**	6E,8E,12E-Hexadecatrien-10-ynoic acid	[M+H]+	C16H20O2	245.15435											4.6E+04	-3.0
**708**	Kynurenine	[M+K]+	C10H12N2O3	247.04839							7.0E+04	-1.8				
**709**	2-Amino-1-methyl-6-phenylimidazo[4,5-b]pyridine	[M+Na]+	C13H12N4	247.09514					7.1E+05	1.1						
**710**	Methyl jasmonate	[M+Na]+	C13H20O3	247.13047	2.9E+05	-2.5							2.1E+05	-2.2		

#	Putative Annotation [a]	Ion	Formula	m/z$_{Theo.}$ [b]	R2020 [c]	ppm	W2020 [c]	ppm	B2020 [c]	ppm	R2021 [c]	ppm	W2021 [c]	ppm	B2021 [c]	ppm
**711**	alpha-Santonin	[M+H]+	C15H18O3	247.13315											5.3E+04	-1.1
**712**	Tetradecadienal	[M+K]+	C14H24O	247.14587	8.6E+04	-1.7										
**713**	Pentadecenal	[M+Na]+	C15H28O	247.20324	8.2E+04	-3.2										
**714**	Proflavine	[M+K]+	C13H11N3	248.05943							5.0E+04	-3.9				
**715**	Lycopodine	[M+H]+	C16H25NO	248.20063							4.2E+04	1.1				
**716**	Tryptanthrine	[M+H]+	C15H8N2O2	249.06638							1.3E+05	-2.1				
**717**	Dihydrochalcone	[M+K]+	C15H14O	249.06762	8.6E+04	-1.2										
**718**	Beta-aspartyl-aspartic acid	[M+H]+	C8H12N2O7	249.07103					7.6E+05	2.8						
**719**	Prenyl caffeate	[M+H]+	C14H16O4	249.11157					4.8E+06	2.3						
**720**	Hydroxymelatonin	[M+H]+	C13H16N2O3	249.12337	1.1E+05	-1.6										
**721**	Canellal	[M+H]+	C15H20O3	249.14852	1.9E+05	-3.1										
**722**	Myristoleic acid	[M+Na]+	C14H26O2	249.18250	8.3E+04	-0.8	2.7E+05	-1.2								
**723**	Hexadecadiynoic acid	[M+H]+	C16H24O2	249.18411											8.0E+04	3.2
**724**	5-(2'-Formylethyl)-4,6-dihydroxypicolinate	[M+K]+	C9H9NO5	250.01066									7.3E+04	2.3		
**725**	1-(4-Methoxyphenyl)-3-(4-morpholinyl)-1-propanone	[M+H]+	C14H19NO3	250.14316									9.4E+04	2.4		
**726**	Serine-phosphoethanolamine	[M+Na]+	C5H13N2O6P	251.04026									1.2E+05	0.3		
**727**	Deoxyuridine	[M+Na]+	C9H12N2O5	251.06470											5.4E+04	-3.4
**728**	L-prolyl-L-proline	[M+K]+	C10H16N2O3	251.07863							1.2E+05	2.5			7.8E+04	-2.3
**729**	3-(5'-Methylthio)pentylmalic acid	[M+H]+	C10H18O5S	251.09386											6.5E+04	3.6
**730**	Ubiquinone Q1	[M+H]+	C14H18O4	251.12791					6.8E+06	-0.5	1.5E+05	2.4			9.7E+04	3.8
**731**	2-methyl-2-dodecenoic acid	[M+K]+	C13H24O2	251.14108									1.6E+05	-1.2		
**732**	2-keto tridecanoic acid	[M+Na]+	C13H24O3	251.16103							7.6E+04	2.9				
**733**	Arbusculin A	[M+H]+	C15H22O3	251.16372									2.0E+05	1.8	6.2E+04	1.0
**734**	Myristic acid	[M+Na]+	C14H28O2	251.19807			3.2E+05	0.3								
**735**	4-Phospho-L-aspartate	[M+K]+	C4H8NO7P	251.96788							4.3E+04	-3.5				
**736**	N-Decanoylglycine	[M+Na]+	C12H23NO3	252.15701	4.9E+05	-1.8							6.1E+05	-0.2		
**737**	Dodecanedioic acid	[M+Na]+	C12H22O4	253.14126							1.5E+05	-0.9				
**738**	Tetramethyl-undecan-1,10-diol	[M+Na]+	C14H30O2	253.21307			1.8E+05	2.9								
**739**	2,2,9,9-tetramethyl-undecan-1,10-diol	[M+Na]+	C14H30O2	253.21380	1.1E+05	-1.4										
**740**	Robustine	[M+K]+	C12H9NO3	254.02052					1.7E+05	3.5						
**741**	N-Nonanoylglycine	[M+K]+	C11H21NO3	254.11618											6.7E+04	-3.5
**742**	Solenopsin A	[M+H]+	C17H35N	254.28423	1.3E+05	-3.9										
**743**	Bisnorbiotin	[M+K]+	C8H12N2O3S	255.02064											1.2E+05	-2.4
**744**	7,4'-Dihydroxyflavone	[M+H]+	C15H10O4	255.06519	7.0E+04	-0.8										
**745**	Arginine phosphate	[M+H]+	C6H15N4O5P	255.08432					1.6E+06	3.8			1.0E+05	2.4		
**746**	N2-Succinyl-L-ornithine	[M+Na]+	C9H16N2O5	255.09429											5.1E+04	3.3
**747**	Methyl 4-[2-(2-formyl-vinyl)-3-hydroxy-5-oxo-cyclopentyl]-butanoate	[M+H]+	C13H18O5	255.12356					2.4E+06	-3.4						
**748**	Hydroxydodecanoic acid	[M+K]+	C12H24O3	255.13634									7.1E+04	-2.5		

#	Putative Annotation [a]	Ion	Formula	$m/z_{Theo.}$ [b]	R2020[c]	ppm	W2020[c]	ppm	B2020[c]	ppm	R2021[c]	ppm	W2021[c]	ppm	B2021[c]	ppm
**749**	2-(Acetamidomethylene)-3-(hydroxymethyl)succinate	[M+K]+	C8H11NO6	256.02156							5.7E+04	0.9				
**750**	Methylphenidate	[M+Na]+	C14H19NO2	256.13004											7.4E+04	3.0
**751**	Palmitic amide	[M+H]+	C16H33NO	256.26349	5.8E+04	0.4										
**752**	2-Oxo-9-methylthiononanoic acid	[M+K]+	C10H18O3S	257.06082	8.6E+04	3.1										
**753**	N-Acetylserotonin	[M+K]+	C12H14N2O2	257.06868							8.0E+04	0.0				
**754**	Abrine	[M+K]+	C12H14N2O2	257.06877											7.2E+04	-0.3
**755**	Hydroxysebacic acid	[M+K]+	C10H18O5	257.07858	2.7E+05	-2.8										
**756**	Phenylglucoside	[M+H]+	C12H16O6	257.10216					3.6E+05	-0.8			1.5E+05	3.6		
**757**	Benzoylagmatine	[M+Na]+	C12H18N4O	257.13641											4.7E+04	3.4
**758**	Curcumenol	[M+Na]+	C15H22O2	257.15154									1.7E+05	-1.3		
**759**	3,3'-Dimethylbisphenol A	[M+H]+	C17H20O2	257.15361	1.3E+05	1.5					4.4E+04	1.5				
**760**	Hexadecatrienal	[M+Na]+	C16H26O	257.18733			1.8E+05	1.0					2.1E+05	-1.1		
**761**	4-Nitrophenyl phosphate	[M+K]+	C6H6NO6P	257.95623											4.0E+04	0.8
**762**	Pentahomomethionine	[M+K]+	C10H21NO2S	258.09246	6.0E+04	3.7										
**763**	Glycerophosphocholine	[M+H]+	C8H20NO6P	258.11010	1.7E+05	3.1							1.1E+05	3.8		
**764**	Glycosminine	[M+Na]+	C15H12N2O	259.08418	1.1E+05	-0.2										
**765**	Capsidiol	[M+Na]+	C15H24O2	259.16664			1.2E+05	0.8								
**766**	Tetradecanedioic acid	[M+H]+	C14H26O4	259.19097							1.6E+05	-2.3				
**767**	Apiole	[M+K]+	C12H14O4	261.05237	5.5E+05	0.7										
**768**	7E,9E,11-Dodecatrienyl acetate	[M+K]+	C14H22O2	261.12514	1.6E+05	0.0										
**769**	1-(4-Hydroxyphenyl)-1-decene-3,5-dione	[M+H]+	C16H20O3	261.14841											2.3E+05	0.4
**770**	3-Hydroxykynurenine	[M+K]+	C10H12N2O4	263.04208							1.3E+05	3.0				
**771**	Beta-aspartyl-glutamic acid	[M+H]+	C9H14N2O7	263.08720					1.4E+06	0.7						
**772**	L-beta-aspartyl-L-glutamic acid	[M+H]+	C9H14N2O7	263.08798			2.2E+05	-2.3								
**773**	Ornaline	[M+H]+	C10H18N2O6	263.12444									2.8E+05	-2.6		
**774**	Marmasmic acid	[M+H]+	C15H18O4	263.12725					2.9E+06	2.0						
**775**	Acutifolane A	[M+H]+	C16H22O3	263.16484									1.4E+05	-2.5		
**776**	Pentadecenoic acid	[M+Na]+	C15H28O2	263.19859			3.9E+05	-1.7								
**777**	Heptadecadiynoic acid	[M+H]+	C17H26O2	263.20046									1.3E+05	0.4		
**778**	Dihydroxy-N-benzoyl-L-serine	[M+Na]+	C10H11NO6	264.04693							5.5E+04	3.5				
**779**	4-(Dimethylamino)azobenzene	[M+K]+	C14H15N3	264.08919							6.6E+04	2.2				
**780**	3-(Phosphoacetylamido)-L-alanine	[M+Na]+	C5H11N2O7P	265.02047											5.4E+04	-3.3
**781**	Nitrotyrosine	[M+K]+	C9H10N2O5	265.02213	7.4E+05	3.5										
**782**	Thymidine	[M+Na]+	C10H14N2O5	265.07955											7.3E+04	-0.2
**783**	N-Acetylcystathionine	[M+H]+	C9H16N2O5S	265.08527	8.0E+05	0.1										
**784**	2'-Hydroxyfurano[2'',3'':4',3']chalcone	[M+H]+	C17H12O3	265.08605					1.0E+06	-0.5						
**785**	2-(6'-Methylthio)hexylmalate	[M+H]+	C11H20O5S	265.11040							7.5E+04	0.1				
**786**	Abscisic acid	[M+H]+	C15H20O4	265.14264			1.2E+05	3.0								
**787**	Keto myristic acid	[M+Na]+	C14H26O3	265.17759			2.7E+05	-0.7					5.6E+05	-3.6		

#	Putative Annotation [a]	Ion	Formula	$m/z_{Theo.}$ [b]	R2020[c]	ppm	W2020[c]	ppm	B2020[c]	ppm	R2021[c]	ppm	W2021[c]	ppm	B2021[c]	ppm
**788**	Dehydrojuvabione	[M+H]+	C16H24O3	265.17963							1.6E+05	0.7			1.8E+05	-1.0
**789**	Arogenate	[M+K]+	C10H13NO5	266.04253	8.8E+04	-0.5										
**790**	Dehydroadenosine	[M+H]+	C10H11N5O4	266.08875											4.6E+04	-1.4
**791**	Annonaine	[M+H]+	C17H15NO2	266.11702											5.6E+04	2.0
**792**	N-Undecanoylglycine	[M+Na]+	C13H25NO3	266.17266	7.7E+05	0.5										
**793**	Amino-tetradecanoic acid	[M+Na]+	C14H29NO2	266.20916									5.9E+04	-0.4		
**794**	Deoxyuridine	[M+K]+	C9H12N2O5	267.03820											5.8E+04	-1.6
**795**	Resveratrol	[M+K]+	C14H12O3	267.04130							6.2E+04	1.9				
**796**	Methyl-dodecanedioic acid	[M+Na]+	C13H24O4	267.15614			1.8E+05	2.0			1.4E+05	3.3	2.9E+05	1.2		
**797**	Myristic acid	[M+K]+	C14H28O2	267.17237							7.1E+04	-1.1			1.5E+05	3.4
**798**	Pentadecanol	[M+K]+	C15H32O	267.20932					6.4E+05	-3.2						
**799**	2-Amino-4-oxo-6-(1',2'-dioxoprolyl)-7,8-dihydroxypteridine	[M+H]+	C9H9N5O5	268.06794											4.8E+04	-1.1
**800**	S-Ribosyl-L-homocysteine	[M+H]+	C9H17NO6S	268.08432											4.7E+04	2.3
**801**	Acetylcarnosine	[M+H]+	C11H15N4O4	268.11561			1.2E+05	3.7								
**802**	N-Decanoylglycine	[M+K]+	C12H23NO3	268.13095	4.6E+05	0.5										
**803**	Aspartyl-L-proline	[M+K]+	C9H14N2O5	269.05293									8.4E+04	1.9	7.3E+04	-2.3
**804**	Saphenic acid	[M+H]+	C15H12N2O3	269.09207	8.0E+04	-3.0										
**805**	Linderenol	[M+K]+	C15H18O2	269.09383							6.0E+04	0.0				
**806**	3-oxo-2-pentyl-cyclopentanehexanoic acid	[M+H]+	C16H28O3	269.21042											1.3E+05	2.6
**807**	7-methyl-6E-hexadecenoic acid	[M+H]+	C17H32O2	269.24751	1.2E+05	2.0										
**808**	N-Succinyl-L-glutamate 5-semialdehyde	[M+K]+	C9H13NO6	270.03671			1.1E+05	2.7								
**809**	N-Acetyl-L-glutamyl 5-phosphate	[M+H]+	C7H12NO8P	270.03725									7.7E+04	0.3		
**810**	Benzoyldehydro-2,3-dihydroxy-benzone	[M+K]+	C13H13NO3	270.05246									6.5E+04	0.9		
**811**	Isovalerylglutamic acid	[M+K]+	C10H17NO5	270.07427					1.5E+05	-1.6						
**812**	O-Butanoylcarnitine	[M+K]+	C11H21NO4	270.10988					3.1E+05	1.2						
**813**	2,3-Dimethoxy-[2-(4-Pyridinyl)-1-butenyl]phenol	[M+H]+	C17H19NO2	270.14796											8.1E+04	3.3
**814**	3,5-Dinitro-4-hydroxyphenylpyruvate	[M+H]+	C9H6N2O8	271.02038											4.5E+04	-2.5
**815**	Melatonin	[M+K]+	C13H16N2O2	271.08434	8.0E+04	3.5										
**816**	Aphylline	[M+Na]+	C15H24N2O	271.17794					2.4E+05	0.5						
**817**	Sterol	[M+Na]+	C17H28O	271.20369			2.7E+05	-1.7								
**818**	Hypusine	[M+K]+	C10H23N3O3	272.13791							5.1E+04	-3.0				
**819**	2-amino-hexadecanoic acid	[M+H]+	C16H33NO2	272.25939											2.1E+05	-3.6
**820**	2-Hydroxy-6-oxo-6-(2-hydroxyphenyl)-hexa-2,4-dienoate	[M+K]+	C12H10O5	273.01598	6.3E+04	1.2									7.3E+04	3.6
**821**	Gamma-Glutamylcysteine	[M+Na]+	C8H14N2O5S	273.05096							7.6E+04	2.2				
**822**	2,2-Dimethyl-8-prenylchromene 6-carboxylic acid	[M+H]+	C17H20O3	273.14833									2.2E+05	0.7		
**823**	Hexadecatrienoic acid	[M+Na]+	C16H26O2	273.18278			4.4E+05	-1.0								

#	Putative Annotation [a]	Ion	Formula	$m/z_{Theo.}$ [b]	R2020[c]	ppm	W2020[c]	ppm	B2020[c]	ppm	R2021[c]	ppm	W2021[c]	ppm	B2021[c]	ppm
**824**	Retamine	[M+Na]+	C15H26N2O	273.19306					1.7E+05	2.5						
**825**	Methylhexadecane-1,2-diol	[M+H]+	C17H36O2	273.27876									1.0E+05	0.2		
**826**	Hexadecasphinganine	[M+H]+	C16H35NO2	274.27350									2.9E+05	2.0	2.0E+05	1.9
**827**	S-(Phenylacetothiohydroximoyl)-L-cysteine	[M+Na]+	C11H14N2O3S	277.06132			1.7E+05	1.5								
**828**	4'-Hydroxy-2'-methoxychalcone	[M+Na]+	C16H14O3	277.08358											4.4E+05	-0.2
**829**	Biotin sulfone	[M+H]+	C10H16N2O5S	277.08443					5.9E+05	3.0						
**830**	methyl 4-[2-(2-formyl-vinyl)-3-hydroxy-5-oxo-cyclopentyl]-butanoate	[M+Na]+	C13H18O5	277.10464	4.0E+05	0.8										
**831**	Palmitoleic acid	[M+Na]+	C16H30O2	277.21380	4.0E+05	1.7	2.0E+06	0.1								
**832**	1,2-Dihydroxynaphthalene-6-sulfonate	[M+K]+	C10H8O5S	278.97192											1.5E+05	1.7
**833**	Ascorbate 2-sulfate	[M+Na]+	C6H8O9S	278.97825			2.1E+05	-0.5								
**834**	Dihydroflavonol	[M+K]+	C15H12O3	279.04220									2.2E+05	-1.4		
**835**	alhpa-tocopheronolactone	[M+H]+	C16H22O4	279.15921			4.1E+06	-0.4							1.8E+06	-1.7
**836**	4-keto pentadecanoic acid	[M+Na]+	C15H28O3	279.19319							5.6E+05	-0.5				
**837**	Palmitic acid	[M+Na]+	C16H32O2	279.22945	1.7E+05	-0.3										
**838**	alpha-linolenic acid	[M+H]+	C18H30O2	279.23125			8.5E+05	2.2								
**839**	Glycerophosphocholine	[M+Na]+	C8H20NO6P	280.09204	1.5E+05	-2.0										
**840**	Apigeniflavan	[M+Na]+	C15H14O4	281.07828					9.9E+05	0.5						
**841**	Glycerophosphocholine	[M+Na]+	C8H21NO6P	281.10076					4.0E+06	-3.2						
**842**	Falcarinone	[M+K]+	C17H22O	281.13014			9.5E+04	0.3							6.9E+04	-3.3
**843**	Phaseic acid	[M+H]+	C15H20O5	281.13875					4.4E+06	-1.4						
**844**	1-(3,4-Dihydroxyphenyl)-5-hydroxy-3-decanone	[M+H]+	C16H24O4	281.17488							4.2E+05	-0.5			3.1E+05	2.9
**845**	N-Undecanoylglycine	[M+K]+	C13H25NO3	282.14660	8.2E+05	0.3										
**846**	Uridine	[M+K]+	C9H12N2O6	283.03186									9.2E+04	3.0		
**847**	Piceatannol	[M+K]+	C14H12O4	283.03682							6.3E+04	-0.4				
**848**	Biotin	[M+K]+	C10H16N2O3S	283.05159			8.9E+04	-0.9								
**849**	Corylinal	[M+H]+	C16H10O5	283.06091									8.5E+04	-2.9		
**850**	Hemigossypol	[M+Na]+	C15H16O4	283.09455					8.6E+05	-1.7						
**851**	Dihydrophaseic acid	[M+H]+	C15H22O5	283.15497					2.4E+06	-3.4						
**852**	Cadiamine	[M+H]+	C15H26N2O3	283.20162	7.3E+04	-2.4										
**853**	Oleic acid	[M+H]+	C18H34O2	283.26316	1.3E+05	2.4										
**854**	Guanosine	[M+H]+	C10H13N5O5	284.09881					1.8E+05	0.5						
**855**	Homovanillic acid sulfate	[M+Na]+	C9H10O7S	285.00449											7.0E+04	-1.9
**856**	Maclurin	[M+Na]+	C13H10O6	285.03715							1.3E+05	-0.7				
**857**	7,4',5'-Trihydroxy-5,2'-oxido-4-phenylcoumarin	[M+H]+	C15H8O6	285.03936	9.7E+04	-2.7										
**858**	Aromaticin	[M+K]+	C15H18O3	285.08917					4.3E+05	-1.5						
**859**	Caffeic acid phenethyl ester	[M+H]+	C17H16O4	285.11214	1.7E+05	-2.3										
**860**	Cernuine	[M+Na]+	C16H26N2O	285.19373	1.4E+05	-2.5										

#	Putative Annotation [a]	Ion	Formula	$m/z_{Theo.}$ [b]	R2020[c]	ppm	W2020[c]	ppm	B2020[c]	ppm	R2021[c]	ppm	W2021[c]	ppm	B2021[c]	ppm
**861**	L-beta-aspartyl-L-aspartic acid	[M+K]+	C8H12N2O7	287.02761	6.9E+04	-3.3										
**862**	Hydroxyindoleacetylglycine	[M+K]+	C12H12N2O4	287.04287	6.6E+04	3.0										
**863**	5'-Phosphoribosylglycinamide	[M+H]+	C7H15N2O8P	287.06463					3.0E+05	-2.6						
**864**	1'-Acetoxyeugenol acetate	[M+Na]+	C14H16O5	287.08870							5.6E+04	1.0				
**865**	4-(Hydroxymethyl)phenyl-beta-D-glucopyranoside	[M+H]+	C13H18O7	287.11263							5.5E+04	-0.4				
**866**	N-Acetyl-leucyl-leucine	[M+H]+	C14H26N2O4	287.19743			2.9E+05	-3.1								
**867**	3,7,11-Trimethyl-2,6,10-dodecatrienyl acetate	[M+Na]+	C17H28O2	287.19891							5.9E+04	-2.6	1.6E+05	-0.4		
**868**	Pyridoxine 5'-phosphate	[M+K]+	C8H12NO6P	288.00406											7.1E+04	-2.4
**869**	C17 Sphinganine	[M+H]+	C17H37NO2	288.29012									1.0E+06	-1.4		
**870**	Xanthoxic acid	[M+Na]+	C15H22O4	289.14103	2.7E+05	-1.9										
**871**	Karwinaphthol B	[M+H]+	C17H20O4	289.14363					5.5E+05	-0.7						
**872**	Methyl 2-(4-isopropyl-4-methyl-5-oxo-2-imidazolin-2-yl)-p-toluate	[M+H]+	C16H20N2O3	289.15473											1.9E+05	-0.2
**873**	4,7,10-hexadecatrienoic acid	[M+K]+	C16H26O2	289.15548									1.9E+05	3.3		
**874**	Elaeocarpidine	[M+Na]+	C17H21N3	290.16276					4.4E+05	0.0						
**875**	Azaadenosine	[M+Na]+	C9H12N6O4	291.08039											3.4E+05	2.9
**876**	Aurin	[M+H]+	C19H14O3	291.10155											1.4E+06	0.1
**877**	5-O-Methylvisamminol	[M+H]+	C16H18O5	291.12173					1.7E+06	3.3						
**878**	Shikimate 3-phosphate	[M+K]+	C7H11O8P	292.98199							7.2E+04	1.1				
**879**	Gingerdione	[M+H]+	C17H24O4	293.17513							7.5E+04	-1.3			9.0E+04	0.5
**880**	Dihydrobunolol	[M+H]+	C17H27NO3	294.20676									1.2E+05	-1.3		
**881**	N-Glycosyl-L-asparagine	[M+H]+	C10H18N2O8	295.11347									1.1E+05	0.4		
**882**	Aspartame	[M+H]+	C14H18N2O5	295.12885	2.6E+06	-1.0									4.0E+05	-2.7
**883**	Methyl-tetradecanedioic acid	[M+Na]+	C15H28O4	295.18836			2.4E+05	-1.3								
**884**	Dimethyladenosine	[M+H]+	C12H17N5O4	296.13446											6.5E+04	2.9
**885**	Apigeniflavan	[M+K]+	C15H14O4	297.05177							6.1E+04	2.0				
**886**	hydroxy-linoleic acid	[M+H]+	C18H32O3	297.24297							2.9E+05	-1.9			2.3E+05	-1.5
**887**	Thiomethyladenosine	[M+H]+	C11H15N5O3S	298.09690					2.2E+05	-0.2						
**888**	Hexanoylcarnitine	[M+K]+	C13H25NO4	298.14172									8.7E+04	-0.7		
**889**	Demethylbellidifolin	[M+K]+	C13H8O6	298.99525	7.5E+04	0.6										
**890**	Prephenyllactate	[M+H]+	C13H14O8	299.07572									1.7E+05	1.4		
**891**	Falcarindiol	[M+K]+	C17H24O2	299.14154							1.9E+05	-2.5	2.5E+05	0.2	1.4E+05	3.0
**892**	Dihydroxy myristoic acid	[M+K]+	C14H28O4	299.16106							3.9E+05	2.9				
**893**	Stearidonic acid	[M+Na]+	C18H28O2	299.19724									4.1E+05	3.0		
**894**	Pyrimidine 5'-deoxynucleotide	[M+Na]+	C9H14N2O6P	300.04861							6.4E+04	-1.5				
**895**	Palmitoylethanolamide	[M+H]+	C18H37NO2	300.28971	2.1E+05	-1.7	3.2E+05	-1.9								
**896**	Artecanin	[M+Na]+	C15H18O5	301.10431					1.8E+05	1.1						
**897**	alhpa-tocopheronolactone	[M+Na]+	C16H22O4	301.14103	1.7E+06	0.8	5.6E+06	-0.6					6.3E+06	-2.3		
**898**	Trimethoxyflavan	[M+H]+	C18H20O4	301.14386							2.4E+06	-1.4				

#	Putative Annotation [a]	Ion	Formula	$m/z_{Theo.}$ [b]	R2020[c]	ppm	W2020[c]	ppm	B2020[c]	ppm	R2021[c]	ppm	W2021[c]	ppm	B2021[c]	ppm
**899**	Pinolenic acid	[M+Na]+	C18H30O2	301.21385					2.3E+05	-0.2						
**900**	Hydroxy stearic acid	[M+H]+	C18H36O3	301.27465			4.2E+05	-3.1								
**901**	Sphinganine	[M+H]+	C18H39NO2	302.30536	9.2E+04	2.1									1.8E+05	-2.6
**902**	3-Methoxy-4-Hydroxyphenylglycol sulfate	[M+K]+	C9H12O7S	302.99329									7.3E+04	0.8		
**903**	Ellagic acid	[M+H]+	C14H6O8	303.01354	7.8E+04	-0.9										
**904**	2-(6'-Methylthio)hexylmalic acid	[M+K]+	C11H20O5S	303.06716									1.2E+05	-2.8		
**905**	2-(4-Methoxyphenethyl)chromone	[M+Na]+	C18H16O3	303.09917	7.5E+04	0.6										
**906**	Abscisic acid	[M+K]+	C15H20O4	303.09957									1.4E+05	-0.8		
**907**	Subaphyllin	[M+K]+	C14H20N2O3	303.11066											1.0E+05	-0.4
**908**	Phaseic acid	[M+Na]+	C15H20O5	303.12029	8.9E+04	-2.2			4.0E+05	-0.4			1.3E+05	1.3		
**909**	Eicosapentaenoic acid	[M+H]+	C20H30O2	303.23186	4.4E+05	-1.9										
**910**	Methyladenosine	[M+Na]+	C11H15N5O4	304.10074			1.6E+05	2.9								
**911**	Arachidonoyl amine	[M+H]+	C20H33NO	304.26439							3.2E+05	-3.0				
**912**	Methylinosine	[M+Na]+	C11H14N4O5	305.08565											1.0E+05	0.0
**913**	Catechin 4'-methyl ether	[M+H]+	C16H16O6	305.10196	1.2E+05	-3.1										
**914**	Oleic acid	[M+Na]+	C18H34O2	305.24525			1.6E+06	-0.5								
**915**	Matricin	[M+H]+	C17H22O5	307.15445					3.3E+06	-1.5						
**916**	3-oxo-2-pentyl-cyclopentanehexanoic acid	[M+K]+	C16H28O3	307.16700	7.8E+04	2.1										
**917**	Retinal	[M+Na]+	C20H28O	307.20327			1.5E+05	-0.1								
**918**	Eicosatrienoic acid	[M+H]+	C20H34O2	307.26320			4.6E+05	-0.1								
**919**	Leu-Gly-Pro	[M+Na]+	C13H23N3O4	308.15785					4.8E+05	0.7						
**920**	4-Hydrocinnamoyl-2,2,5-trimethyl-4-cyclopentene-1,3-dione	[M+K]+	C17H18O3	309.08899					1.2E+06	-0.8						
**921**	Fructoselysine	[M+H]+	C12H24N2O7	309.16591							9.7E+04	-0.9				
**922**	O-Methylembelin	[M+H]+	C18H28O4	309.20604	2.3E+05	-2.5									1.2E+05	-2.2
**923**	Tridecanoylglycine	[M+K]+	C15H29NO3	310.17700					4.3E+05	2.9						
**924**	Gamma-Glutamyltyrosine	[M+H]+	C14H18N2O6	311.12376	1.8E+05	0.7							8.2E+04	-1.6		
**925**	Cupreine	[M+H]+	C19H22N2O2	311.17540	1.0E+05	2.1										
**926**	9-hydroperoxy-10E,12,15Z-octadecatrienoic acid	[M+H]+	C18H30O4	311.22229							6.3E+04	-1.9				
**927**	Thyronine	[M+K]+	C15H15NO4	312.06260											1.1E+05	2.1
**928**	Taxiphyllin	[M+H]+	C14H17NO7	312.10849									1.0E+05	-2.3		
**929**	Hydroxycinnamyl alcohol 4-D-glucoside	[M+H]+	C15H20O7	313.12852									1.4E+05	-1.1		
**930**	Sauroxine	[M+K]+	C17H26N2O	313.16700					1.2E+06	2.1					1.5E+05	-2.4
**931**	Hydroperoxylinoleic acid	[M+H]+	C18H32O4	313.23814									4.9E+05	-2.6		
**932**	Beta-D-Glucopyranuronic acid	[M+H]+	C13H14O9	315.07106	6.9E+04	0.5										
**933**	Coumatetralyl	[M+Na]+	C19H16O3	315.09951					3.1E+05	-1.1						
**934**	Grevillol	[M+Na]+	C19H32O2	315.23037											7.8E+04	-2.9
**935**	Octadecanedioic acid	[M+H]+	C18H34O4	315.25299	7.0E+05	-0.7	1.3E+06	-1.4								
**936**	Rosinidin	[M+H]+	C17H15O6	316.09414	6.8E+04	2.9										

#	Putative Annotation [a]	Ion	Formula	$m/z_{Theo.}$ [b]	R2020[c]	ppm	W2020[c]	ppm	B2020[c]	ppm	R2021[c]	ppm	W2021[c]	ppm	B2021[c]	ppm
**937**	Melosatin B	[M+Na]+	C19H19NO2	316.13098							9.5E+04	-0.6				
**938**	Dehydrophytosphingosine	[M+H]+	C18H37NO3	316.28462	9.1E+05	-0.3							3.0E+05	-0.9		
**939**	L-phenylalanyl-L-hydroxyproline	[M+K]+	C14H18N2O4	317.08985							1.1E+05	-0.1			1.6E+05	2.9
**940**	alhpa-tocopheronolactone	[M+K]+	C16H22O4	317.11479			1.5E+06	0.6					2.9E+05	1.8		
**941**	2-Butyl-3-(4-hydroxybenzoyl)benzofuran	[M+Na]+	C19H18O3	317.11482	2.7E+06	-0.7										
**942**	alhpa-tocopheronolactone	[M+K]+	C16H22O4	317.11500							1.2E+05	-0.1				
**943**	4-Hydroxy-5,7,4'-trimethoxyflavan	[M+H]+	C18H20O5	317.13903					2.4E+05	-2.1			2.8E+05	2.4		
**944**	Gibberellin A9	[M+H]+	C19H24O4	317.17486									3.5E+05	-0.4		
**945**	2-hydroxy-9Z,12Z,15Z-octadecatrienoic acid	[M+Na]+	C18H30O3	317.20794											1.2E+05	2.4
**946**	Eicosenal	[M+Na]+	C20H38O	317.28081			1.1E+05	2.1								
**947**	Dimethyladenosine	[M+Na]+	C12H17N5O4	318.11762			2.9E+05	-1.1								
**948**	Phytosphingosine	[M+H]+	C18H39NO3	318.30027	1.0E+06	0.6										
**949**	L-beta-aspartyl-L-phenylalanine	[M+K]+	C13H16N2O5	319.06830					2.7E+05	2.4						
**950**	Methylaminoadenosine	[M+Na]+	C11H16N6O4	319.11168			1.7E+05	2.6								
**951**	Linoleic acid	[M+K]+	C18H32O2	319.20334			2.7E+05	0.2								
**952**	Oleamide	[M+K]+	C18H35NO	320.23580									8.6E+04	-2.4		
**953**	2,3-Diphenyl-1-indanone	[M+K]+	C21H14O	321.06736							8.2E+04	0.8				
**954**	Methylguanosine	[M+Na]+	C11H16N5O5	321.10436	1.3E+05	2.9										
**955**	Mugineic acid	[M+H]+	C12H20N2O8	321.12968									3.0E+05	-1.4		
**956**	Dehydroretinal	[M+K]+	C20H26O	321.16073			1.2E+05	2.5								
**957**	Quebrachamine	[M+K]+	C19H26N2	321.17220											2.7E+05	1.7
**958**	Hydroxy-arachidonic acid	[M+H]+	C20H32O3	321.24320			2.3E+05	-2.4								
**959**	8-Anilino-1-naphthalene sulfonic acid	[M+Na]+	C16H13NO3S	322.05083	8.4E+04	-1.1										
**960**	Digallic acid	[M+H]+	C14H10O9	323.03997							7.4E+04	-0.7				
**961**	2'-Hydroxy-4',6'-dimethoxy-3'-methyldihydrochalcone	[M+Na]+	C18H20O4	323.12538	1.7E+05	0.7										
**962**	Tetranor-PGF1alpha	[M+Na]+	C16H28O5	323.18336							7.4E+04	-1.4				
**963**	Retinoic acid	[M+Na]+	C20H28O2	323.19815	1.1E+05	-1.5	9.9E+04	0.8								
**964**	Gingerol	[M+H]+	C19H30O4	323.22086											1.7E+05	2.6
**965**	Stearic acid	[M+K]+	C18H36O2	323.23451					9.5E+05	0.6						
**966**	Hydroxystearate	[M+Na]+	C18H36O3	323.25616					5.3E+05	-1.5						
**967**	Myristoylglycine	[M+K]+	C16H31NO3	324.19355	7.7E+04	0.0										
**968**	Galactosylhydroxylysine	[M+H]+	C12H24N2O8	325.16132									2.2E+05	-2.4		
**969**	12-oxo-14,18-dihydroxy-9Z,13E,15Z-octadecatrienoic acid	[M+H]+	C18H28O5	325.20095	1.1E+05	-1.0										
**970**	Alchornoic acid	[M+H]+	C20H36O3	325.27453									4.4E+05	-2.5		
**971**	Farnesylcysteine	[M+H]+	C18H31NO2S	326.21406			1.3E+05	2.4								
**972**	Dihydrokaempferol	[M+K]+	C15H12O6	327.02709			1.1E+05	-1.7								
**973**	Hydroxyflemichapparin C	[M+H]+	C17H10O7	327.05057					3.3E+05	-2.0						
**974**	Hinokitiol glucoside	[M+H]+	C16H22O7	327.14355					1.1E+06	0.9						

#	Putative Annotation [a]	Ion	Formula	$m/z_{Theo.}$ [b]	R2020[c]	ppm	W2020[c]	ppm	B2020[c]	ppm	R2021[c]	ppm	W2021[c]	ppm	B2021[c]	ppm
**975**	Acutifolin A	[M+H]+	C20H22O4	327.15980							1.6E+05	-2.2				
**976**	Capsaicin	[M+Na]+	C18H27NO3	328.18836							7.6E+04	-0.1				
**977**	Lonchocarpin	[M+Na]+	C20H18O3	329.11480					3.0E+05	0.0						
**978**	Eicosatrienoic acid	[M+Na]+	C20H34O2	329.24577			9.6E+04	-2.0								
**979**	Adenosine 2',3'-cyclic phosphate	[M+H]+	C10H12N5O6P	330.05980	1.2E+05	0.7										
**980**	Stealthin C	[M+Na]+	C18H13NO4	330.07322							1.2E+05	1.4				
**981**	Glutathione	[M+Na]+	C10H17N3O6S	330.07400									1.3E+05	-3.0		
**982**	Gingerdione	[M+K]+	C17H24O4	331.13139							1.2E+05	-2.3				
**983**	Cinnamodial	[M+Na]+	C17H24O5	331.15159	1.9E+05	-1.6										
**984**	5'-O-beta-D-Glucosylpyridoxine	[M+H]+	C14H21NO8	332.13399	2.4E+05	-1.9										
**985**	Megastachine	[M+H]+	C20H29NO3	332.22188							8.5E+04	0.4				
**986**	Ovalitenin B	[M+Na]+	C19H18O4	333.10918									2.1E+05	1.7		
**987**	Gibberellin A20	[M+H]+	C19H24O5	333.17003					6.5E+05	-1.1						
**988**	Hydroxylinolenic acid	[M+K]+	C18H30O3	333.18265	1.2E+05	-1.7										
**989**	9-hydroperoxy-10E,12,15Z-octadecatrienoic acid	[M+Na]+	C18H30O4	333.20300							8.3E+04	1.9				
**990**	Tintinnadiol	[M+H]+	C21H32O3	333.24282									1.5E+05	-1.2		
**991**	18-hydroxy-9S,10R-dihydroxy-stearic acid	[M+H]+	C18H36O5	333.26324											8.0E+04	0.9
**992**	Spiradine A	[M+Na]+	C20H25NO2	334.17688					1.7E+05	2.6						
**993**	Arabino-galactose	[M+Na]+	C11H20O10	335.09487	1.2E+05	-0.8										
**994**	alhpa-tocopheronic acid	[M+K]+	C16H24O5	335.12613							1.2E+05	-1.8	3.9E+05	-2.3		
**995**	Oxo-nonadecanoic acid	[M+Na]+	C19H36O3	335.25641			1.7E+05	-2.2								
**996**	Palmitoleoyl Ethanolamide	[M+K]+	C18H35NO2	336.22935					2.6E+05	1.8						
**997**	2-amino-14,16-dimethyloctadecan-3-ol	[M+Na]+	C20H43NO	336.32272											7.2E+04	2.9
**998**	Epihydroxymugineic acid	[M+H]+	C12H20N2O9	337.12339					1.5E+06	2.3						
**999**	Astrophylline	[M+K]+	C19H26N2O	337.16767	9.8E+04	1.7										
**1000**	Apovincamine	[M+H]+	C21H24N2O2	337.19025									3.8E+05	2.4		
**1001**	Octadecanedioic acid	[M+Na]+	C18H34O4	337.23532			2.7E+06	-1.2								
**1002**	Belladine	[M+Na]+	C19H25NO3	338.17266	1.4E+05	-2.1										
**1003**	Pentadecanoylglycine	[M+K]+	C17H33NO3	338.20919					2.8E+05	0.0						
**1004**	Peroxyferolide	[M+H]+	C17H22O7	339.14283					2.9E+06	2.9						
**1005**	2,3-dihydroxy stearic acid	[M+Na]+	C18H36O4	339.25077									1.5E+05	-0.6		
**1006**	Ormosanine	[M+Na]+	C20H35N3	340.27158							1.8E+05	2.2				
**1007**	Cellobiono-1,5-lactone	[M+H]+	C12H20O11	341.10784	8.6E+04	-1.0										
**1008**	2-Phenethylsulfanyl-5,6,7,8-tetrahydro-benzo[4,5]thieno[2,3-d]pyrimidin-4-ylamine	[M+H]+	C18H19N3S2	342.10901					1.9E+05	0.9						
**1009**	Caffeic acid 3-glucoside	[M+H]+	C15H18O9	343.10230									1.0E+05	0.2		
**1010**	Coniferin	[M+H]+	C16H22O8	343.13874	8.2E+04	0.2										
**1011**	Phellodendrine	[M+H]+	C20H24NO4	343.17749					9.3E+05	0.9						
**1012**	Arachidonic acid	[M+K]+	C20H32O2	343.20344			1.3E+05	-0.1								

#	Putative Annotation [a]	Ion	Formula	$m/z_{Theo.}$ [b]	R2020[c]	ppm	W2020[c]	ppm	B2020[c]	ppm	R2021[c]	ppm	W2021[c]	ppm	B2021[c]	ppm
**1013**	4-Carboxy-4'-sulfoazobenzene	[M+K]+	C13H10N2O5S	344.99420	8.9E+04	1.7										
**1014**	Ovalitenin C	[M+Na]+	C19H14O5	345.07259									8.6E+04	2.2		
**1015**	6-O-alpha-D-Galactosyl-D-glucitol	[M+H]+	C12H24O11	345.13883					1.9E+05	0.9						
**1016**	Hydroxyeicosatrienoic acid	[M+Na]+	C20H34O3	345.24069							2.5E+05	-2.0				
**1017**	1alpha-hydroxy-22-oxo-23,24,25,26,27-pentanorvitamin D3	[M+H]+	C22H32O3	345.24154									2.1E+05	2.6		
**1018**	Fructose 1,6-bisphosphate	[M+Na]+	C6H14O11P2	346.99036	8.8E+04	-2.8										
**1019**	Dimethoxy-furanoflavanone	[M+Na]+	C19H16O5	347.08933			1.1E+05	-1.0								
**1020**	Cinnamodial	[M+K]+	C17H24O5	347.12558			1.2E+05	-0.1								
**1021**	Tetraneurin E	[M+Na]+	C17H24O6	347.14668							8.4E+04	-0.5				
**1022**	Gibberellin A24	[M+H]+	C20H26O5	347.18530	1.8E+05	-1.7										
**1023**	5-(Heptadec-12-enyl)resorcinol	[M+H]+	C23H38O2	347.29444							7.1E+04	0.1				
**1024**	Hydroxysanguinarine	[M+H]+	C20H13NO5	348.08665	6.8E+04	1.4										
**1025**	N-(3-Hydroxy-7-cis-tetradecenoyl)homoserine lactone	[M+Na]+	C18H31NO4	348.21437									1.0E+05	0.5		
**1026**	Carboxy-alpha-chromanol	[M+H]+	C21H31O4	348.22874			1.5E+05	2.2								
**1027**	O-Arachidonoyl Ethanolamine	[M+H]+	C22H37NO2	348.28918			1.5E+05	1.5								
**1028**	Inosinic acid	[M+H]+	C10H13N4O8P	349.05438	6.8E+04	2.5										
**1029**	Calopogoniumisoflavone B	[M+H]+	C21H16O5	349.10664			1.3E+05	1.2								
**1030**	Apigenin 7-sulfate	[M+H]+	C15H10O8S	351.01621							8.0E+04	2.0				
**1031**	Desmosdumotin C	[M+K]+	C19H20O4	351.09932	3.1E+05	-1.7										
**1032**	Jatrophone	[M+K]+	C20H24O3	351.13637									1.3E+05	-1.9		
**1033**	Rhododendrin	[M+Na]+	C16H24O7	351.14120					1.5E+06	0.6						
**1034**	Gibberellin A2	[M+H]+	C19H26O6	351.18064					2.5E+06	-1.2						
**1035**	9,12,13-trihydroxy-10,15-octadecadienoic acid	[M+Na]+	C18H32O5	351.21389							2.0E+05	0.9				
**1036**	Coumestrol diacetate	[M+H]+	C19H12O7	353.06612			1.6E+05	-1.5								
**1037**	Carnosol	[M+Na]+	C20H26O4	353.17290							9.4E+04	-1.6				
**1038**	Jasmolin I	[M+Na]+	C21H30O3	353.20872	5.9E+05	-1.2										
**1039**	Geranylgeranylacetone	[M+Na]+	C23H38O	353.28192							1.4E+05	-1.2				
**1040**	N,N-dimethyl arachidonoyl amine	[M+Na]+	C22H37NO	354.27633			9.8E+04	1.1								
**1041**	8,12,16,19-docosatetraenoic acid	[M+Na]+	C22H36O2	355.26063							3.2E+05	0.3				
**1042**	Spirasine I	[M+H]+	C22H29NO3	356.22237							1.1E+05	-1.0				
**1043**	Feruloyl-D-glucose	[M+H]+	C16H20O9	357.11858									2.2E+05	-1.6		
**1044**	Ubiquinone Q2	[M+K]+	C19H26O4	357.14568											1.2E+05	1.6
**1045**	Vinorine	[M+Na]+	C21H22N2O2	357.15691					4.8E+05	1.2						
**1046**	Estradiol valerate	[M+H]+	C23H32O3	357.24242	2.7E+05	-0.8										
**1047**	1-Carbazol-9-yl-3-(3,5-dimethylpyrazol-1-yl)-propan-2-ol	[M+K]+	C20H21N3O	358.13162	6.8E+04	2.4										
**1048**	Bakankoside	[M+H]+	C16H23NO8	358.15011									1.7E+05	-1.3		
**1049**	Leucyl-leucyl-norleucine	[M+H]+	C18H35N3O4	358.27086											1.1E+05	-2.3

#	Putative Annotation [a]	Ion	Formula	$m/z_{Theo.}$ [b]	R2020[c]	ppm	W2020[c]	ppm	B2020[c]	ppm	R2021[c]	ppm	W2021[c]	ppm	B2021[c]	ppm
**1050**	Coumeroic acid	[M+H]+	C17H14N2O7	359.08714											8.3E+04	0.7
**1051**	Docosapentaynoic acid	[M+K]+	C22H24O2	359.14079	7.0E+04	1.6										
**1052**	Ficine	[M+Na]+	C20H19NO4	360.12063	8.3E+04	-1.2										
**1053**	Thymidylic acid	[M+K]+	C10H15N2O8P	361.01981			1.3E+05	-0.1								
**1054**	Peroxyferolide	[M+Na]+	C17H22O7	361.12507					2.3E+05	1.9						
**1055**	N-Arachidonoyl glycine	[M+H]+	C22H35NO3	362.26982											8.1E+04	-2.3
**1056**	5,7,3'-Trihydroxy-6,4',5'-trimethoxyflavanone	[M+H]+	C18H18O8	363.10744	2.1E+05	2.2										
**1057**	Gibberellin A19	[M+H]+	C20H26O6	363.18012					1.5E+06	0.3						
**1058**	Docosanoic acid	[M+Na]+	C22H44O2	363.32371							1.1E+05	-1.0				
**1059**	Lactosamine	[M+Na]+	C12H23NO10	364.12053					2.5E+05	2.4						
**1060**	PC(0:0/5:0)	[M+Na]+	C13H28NO7P	364.14985					2.5E+05	-0.8						
**1061**	N-Oleoylethanolamine	[M+K]+	C20H39NO2	364.26168							1.7E+05	-1.2				
**1062**	Xanthylic acid	[M+H]+	C10H13N4O9P	365.04974											7.6E+04	-1.2
**1063**	Lactose	[M+Na]+	C12H22O11	365.10543	3.0E+06	-1.3							4.2E+05	2.0		
**1064**	Brosimacutin C	[M+Na]+	C20H22O5	365.13632			9.8E+05	-1.0								
**1065**	Gibberellin A8	[M+H]+	C19H24O7	365.15901											2.6E+05	1.3
**1066**	Gibberellin A37	[M+H]+	C20H28O6	365.19606					2.1E+06	-0.5						
**1067**	Homophytanic acid	[M+K]+	C21H42O2	365.28191							1.1E+05	-0.7				
**1068**	PC(O-2:0/2:0)	[M+K]+	C12H26NO7P	366.10785	4.4E+05	-1.7	2.1E+05	0.3								
**1069**	2-(acetylamino)-1,5-anhydro-2-deoxy-3-O-b-D-galactopyranosyl-D-arabino-Hex-1-enitol	[M+H]+	C14H23NO10	366.13961			3.0E+05	-0.4								
**1070**	Dolichyl diphosphate	[M+Na]+	C12H26O7P2	367.10460	8.9E+04	-0.2										
**1071**	Deoxyelephantopin	[M+Na]+	C19H20O6	367.11550									8.9E+04	-0.8		
**1072**	Rhododendrin	[M+K]+	C16H24O7	367.11550									8.9E+04	-0.4		
**1073**	Boschnaloside	[M+Na]+	C16H24O8	367.13570					1.2E+06	1.7						
**1074**	12,13-dihydroxy-11-methoxy-9-octadecenoic acid	[M+Na]+	C19H36O5	367.24605											1.1E+05	-1.5
**1075**	Prostaglandin G2	[M+H]+	C20H32O6	369.22747									1.1E+05	-0.8		
**1076**	Mahanimbine	[M+K]+	C23H25NO	370.15776					2.8E+05	-2.7						
**1077**	Prostaglandin G1	[M+H]+	C20H33O6	370.23421					2.1E+05	2.1						
**1078**	Fraxin	[M+H]+	C16H18O10	371.09736									1.6E+05	-0.2		
**1079**	Leonuridine	[M+Na]+	C15H24O9	371.13116											1.2E+05	0.3
**1080**	Carnosic acid	[M+K]+	C20H28O4	371.16219							2.1E+05	-0.7				
**1081**	Naphthylacetylspermine	[M+H]+	C22H34N4O	371.28066											6.6E+05	-0.3
**1082**	N-Acetylphenylalanine beta-naphthyl ester	[M+K]+	C21H19NO3	372.09965	8.3E+04	-0.5					8.9E+04	1.4				
**1083**	Eicosatrienoylethanolamide	[M+Na]+	C22H39NO2	372.28808							1.4E+05	-2.1				
**1084**	Calopogoniumisoflavone A	[M+K]+	C21H18O4	373.08367	1.0E+05	1.9										
**1085**	Cathenamine	[M+Na]+	C21H22N2O3	373.15241									9.2E+04	-0.4		
**1086**	Gingerol	[M+Na]+	C21H34O4	373.23484							4.2E+05	0.2	2.0E+05	-1.9		
**1087**	5,7,4'-Trihydroxyflavanone 7-sulfate	[M+Na]+	C15H12O8S	375.01451	8.6E+04	0.5	9.5E+04	0.2								

#	Putative Annotation [a]	Ion	Formula	$m/z_{Theo.}$ [b]	R2020[c]	ppm	W2020[c]	ppm	B2020[c]	ppm	R2021[c]	ppm	W2021[c]	ppm	B2021[c]	ppm
**1088**	Umbellatine	[M+K]+	C20H18NO4	375.08708									1.3E+05	-0.9		
**1089**	Myricetin 3,7,3',4'-tetramethyl ether	[M+H]+	C19H18O8	375.10701					1.8E+05	1.2						
**1090**	Phantomolin	[M+H]+	C21H26O6	375.17948					3.1E+05	2.0						
**1091**	Picrasin B	[M+H]+	C21H28O6	377.19516					7.6E+05	1.9						
**1092**	Methyl-docosanoic acid	[M+Na]+	C23H46O2	377.33887			1.2E+05	0.3								
**1093**	Feruloyl-D-glucose	[M+Na]+	C16H20O9	379.09981			1.1E+05	0.4								
**1094**	gibberellin A17	[M+H]+	C20H26O7	379.17455					2.0E+06	1.5						
**1095**	Arachidonoylglycerol	[M+H]+	C23H38O4	379.28423			2.3E+05	0.1								
**1096**	Lactosamine	[M+K]+	C12H23NO10	380.09536	1.8E+05	1.4										
**1097**	Lactose	[M+K]+	C12H22O11	381.07937	3.9E+06	-0.4										
**1098**	Brosimacutin C	[M+K]+	C20H22O5	381.11071			2.4E+05	-2.2								
**1099**	PA(14:1(9Z)/0:0)	[M+H]+	C17H33O7P	381.20344							3.3E+05	0.6				
**1100**	Nogalonic acid	[M+H]+	C20H14O8	383.07614	3.3E+05	-0.5			4.2E+05	0.9						
**1101**	Brosimacutin H	[M+Na]+	C20H24O6	383.14651	2.1E+05	-1.9										
**1102**	1alpha,23-dihydroxy-24,25,26,27-tetranorvitamin D3	[M+Na]+	C23H36O3	383.25547			1.2E+05	0.5								
**1103**	10,11-dihydroxy-4Z,7Z,13Z,16Z,19Z-docosapentaenoic acid	[M+Na]+	C22H34O4	385.23416											9.9E+04	2.0
**1104**	MG(0:0/20:1(11Z)/0:0)	[M+H]+	C23H44O4	385.33099			1.6E+05	0.6								
**1105**	8-Cinnamoyl-3,4-dihydro-5,7-dihydroxy-4-phenylcoumarin	[M+H]+	C24H18O5	387.12215									8.4E+04	1.4		
**1106**	4-O-beta-D-Glucosyl-sinapate	[M+H]+	C17H22O10	387.12857	7.0E+04	-2.3										
**1107**	DG(18:0e/2:0/0:0)	[M+H]+	C23H46O4	387.34742									9.0E+04	-1.4		
**1108**	Quassin	[M+H]+	C22H28O6	389.19597					3.3E+05	-0.3	1.4E+05	-1.5				
**1109**	5-Hydroxy-4'-methoxy-6'',6''-dimethylpyrano	[M+K]+	C21H20O5	391.09343									1.3E+05	2.1		
**1110**	Apocholic acid	[M+H]+	C24H38O4	391.28520			1.8E+06	-2.3								
**1111**	Deoxyvitamin D3	[M+Na]+	C27H44	391.33349					2.5E+05	0.1						
**1112**	Prostaglandin G1	[M+Na]+	C20H33O6	392.21693	7.7E+04	2.4										
**1113**	Heteratisine	[M+H]+	C22H33NO5	392.24329											1.2E+05	-0.4
**1114**	Chlorogenic acid	[M+K]+	C16H18O9	393.05894					4.8E+05	-1.8						
**1115**	Fraxin	[M+Na]+	C16H18O10	393.07922	1.6E+05	-0.8							7.2E+05	2.1		
**1116**	Picrasin G	[M+H]+	C21H28O7	393.19065					1.5E+06	0.3						
**1117**	Docosanedioic acid	[M+Na]+	C22H42O4	393.29832			5.2E+05	-2.0								
**1118**	Murocholic acid	[M+H]+	C24H40O4	393.29994	4.9E+05	-2.4										
**1119**	Spirasine I	[M+K]+	C22H29NO3	394.17752									1.8E+05	1.0		
**1120**	Syringin	[M+Na]+	C17H24O9	395.13100					7.4E+05	0.6						
**1121**	3-Geranyl-4,2',4'-trihydroxydihydrochalcone	[M+H]+	C25H30O4	395.22239									1.5E+05	-1.8		
**1122**	MG(0:0/18:1(11Z)/0:0)	[M+K]+	C21H40O4	395.25582	1.5E+05	0.7										
**1123**	Conessine	[M+K]+	C24H40N2	395.28194					4.6E+05	0.9						
**1124**	Phenylaminoadenosine	[M+K]+	C16H18N6O4	397.10211	3.4E+05	0.3										

#	Putative Annotation [a]	Ion	Formula	$m/z_{Theo.}$ [b]	R2020[c]	ppm	W2020[c]	ppm	B2020[c]	ppm	R2021[c]	ppm	W2021[c]	ppm	B2021[c]	ppm
**1125**	Icaceine	[M+Na]+	C22H33NO4	398.22933					2.5E+05	2.1						
**1126**	Myricetin 7,3',4'-trimethyl ether	[M+K]+	C18H16O8	399.04768	1.6E+05	-0.6										
**1127**	Calicoferol E	[M+H]+	C27H42O2	399.32513			1.1E+05	1.6								
**1128**	5,7,3',4',5'-Pentahydroxy-3,6-dimethoxyflavone	[M+K]+	C17H14O9	401.02607					1.7E+05	2.2						
**1129**	MG(0:0/20:4(8Z,11Z,14Z,17Z)/0:0)	[M+Na]+	C23H38O4	401.26642							3.9E+05	-0.5				
**1130**	Xanthylic acid	[M+K]+	C10H13N4O9P	403.00503							9.8E+04	0.4				
**1131**	Cinncassiol C1	[M+Na]+	C20H28O7	403.17243					2.8E+05	0.7						
**1132**	delta-Tocopherol	[M+H]+	C27H46O2	403.35706	1.4E+05	1.7										
**1133**	Cinncassiol C3	[M+Na]+	C20H30O7	405.18837	1.2E+05	1.6										
**1134**	Alpha-Linoleoylcholine	[M+K]+	C23H44NO2	405.30058											1.4E+05	-0.5
**1135**	Pentacosanal	[M+K]+	C25H50O	405.35023					3.3E+05	-2.2						
**1136**	Rhyncophylline	[M+Na]+	C22H28N2O4	407.19388									6.8E+05	0.6		
**1137**	Picrasin E	[M+H]+	C22H30O7	407.20626					1.5E+06	0.4						
**1138**	MG(0:0/20:1(11Z)/0:0)	[M+Na]+	C23H44O4	407.31303											8.0E+04	0.4
**1139**	Ovatine	[M+Na]+	C24H35NO3	408.25012									9.7E+04	2.0		
**1140**	Geranylgeranylcysteine	[M+H]+	C23H37NO3S	408.25600											7.8E+04	1.7
**1141**	Isorobustin 4-methyl ether	[M+H]+	C23H20O7	409.12837											1.6E+05	-0.5
**1142**	Docosanedioic acid	[M+K]+	C22H42O4	409.27147	6.3E+05	-0.7										
**1143**	Hydroxy-tricosanoic acid	[M+K]+	C23H46O3	409.30883			1.6E+05	-2.4								
**1144**	Carboxy-gama-tocotrienol	[M+K]+	C23H31O4	410.18546					3.0E+05	-0.2						
**1145**	N-palmitoyl methionine	[M+Na]+	C21H41NO3S	410.27008					2.1E+05	-0.4						
**1146**	Bensulfuron methyl	[M+H]+	C16H18N4O7S	411.09690	9.5E+04	1.1										
**1147**	Spinoflavanone A	[M+Na]+	C25H24O4	411.15745							8.1E+04	-1.9				
**1148**	5-Methoxy-7,8-diprenylflavone	[M+Na]+	C26H28O3	411.19273											8.2E+04	0.8
**1149**	Rehmaionoside C	[M+Na]+	C19H32O8	411.19966							1.4E+05	-1.8				
**1150**	Vitamin D6	[M+H]+	C29H46O	411.36300											7.2E+04	-2.1
**1151**	Curacin A	[M+K]+	C23H35NOS	412.20611					3.6E+05	2.4						
**1152**	N-oleoyl taurine	[M+Na]+	C20H39NO4S	412.24919					2.0E+05	0.0						
**1153**	11-hydroxy-9,10-dihydrojasmonic acid 11-beta-D-glucoside	[M+Na]+	C18H30O9	413.17767					7.9E+05	1.3						
**1154**	Apocholic acid	[M+Na]+	C24H38O4	413.26690			3.2E+06	-1.6							3.3E+05	-2.2
**1155**	N-stearoyl glutamine	[M+H]+	C23H44N2O4	413.33825									6.6E+05	-2.1		
**1156**	Hexacosatrienoic acid	[M+Na]+	C26H46O2	413.33981							1.0E+06	-2.0				
**1157**	Hydroxy-hexacosanoic acid	[M+H]+	C26H52O3	413.39892	2.6E+05	2.0										
**1158**	Anguvetin	[M+Na]+	C24H24O5	415.15103					3.2E+05	1.4						
**1159**	Phytyl phosphate	[M+K]+	C20H41O4P	415.23813					3.5E+05	-1.7						
**1160**	Lupulone	[M+H]+	C26H38O4	415.28429	1.8E+05	1.1										
**1161**	MG(0:0/22:0/0:0)	[M+H]+	C25H50O4	415.37792			1.7E+05	0.6								
**1162**	Spironolactone	[M+H]+	C24H32O4S	417.20941	2.2E+05	-1.6										

#	Putative Annotation [a]	Ion	Formula	$m/z_{Theo.}$ [b]	R2020[c]	ppm	W2020[c]	ppm	B2020[c]	ppm	R2021[c]	ppm	W2021[c]	ppm	B2021[c]	ppm
**1163**	Calcitriol	[M+H]+	C27H44O3	417.33556											5.8E+05	1.8
**1164**	PGF2alpha isopropyl ester	[M+Na]+	C23H40O5	419.27699									3.0E+05	-0.5		
**1165**	1alpha,25-dihydroxy-22-oxavitamin D3	[M+H]+	C26H42O4	419.31566			1.3E+05	-0.2							1.3E+05	-2.0
**1166**	8'-apo-beta-Carotenol	[M+H]+	C30H42O	419.33163							1.3E+05	-1.9				
**1167**	Hexacosanal	[M+K]+	C26H52O	419.36417					2.5E+05	1.9						
**1168**	Lisetin	[M+K]+	C21H18O7	421.06808					2.8E+05	0.8						
**1169**	N-Benzoyl-D-arginine-4-nitroanilide	[M+Na]+	C19H22N6O4	421.15998									1.0E+05	-1.2		
**1170**	C17 Sphingosine-1-phosphate	[M+K]+	C17H39N2O5P	421.22284					1.1E+06	-0.1						
**1171**	8-(2-Carboxy-1-phenylethyl)-3,5,7-trihydroxyflavone delta-lactone	[M+Na]+	C24H16O6	423.08292					3.7E+05	2.3						
**1172**	Carboxy-alpha-tocotrienol	[M+K]+	C24H33O4	424.20155					3.3E+05	-1.2			1.0E+05	-2.4		
**1173**	Myxalamid B	[M+Na]+	C25H39NO3	424.28221	2.3E+05	0.7										
**1174**	N-(2-phenoxy-ethyl) arachidonoyl amine	[M+H]+	C28H41NO2	424.32164			1.3E+05	-1.5							8.3E+04	0.6
**1175**	Diferulic acid	[M+K]+	C20H18O8	425.06246					3.9E+05	2.0						
**1176**	delta-Tocopherol	[M+Na]+	C27H46O2	425.33807							7.6E+04	2.2				
**1177**	Chitobiose	[M+H]+	C15H26N2O12	427.15601			2.0E+05	-0.4								
**1178**	MG(0:0/22:5(4Z,7Z,10Z,13Z,16Z)/0:0)	[M+Na]+	C25H40O4	427.28247					4.5E+05	-1.4						
**1179**	Hydroxy-gama-tocotrienol	[M+H]+	C28H42O3	427.32021					2.6E+05	1.1						
**1180**	3-Deoxy-3-azido-25-hydroxyvitamin D3	[M+H]+	C27H44N3O	427.35528							5.1E+05	1.0				
**1181**	Rubraflavone A	[M+Na]+	C25H26O5	429.16814					3.4E+05	-2.1						
**1182**	Aliarin 4'-methyl ether	[M+H]+	C23H26O8	431.16945					2.2E+05	1.4						
**1183**	Dimethamine	[M+Na]+	C24H32N4O2	431.24175	5.1E+05	1.3										
**1184**	Cholic acid	[M+Na]+	C24H40O5	431.27657											1.8E+05	0.5
**1185**	Hexacosadienoic acid	[M+K]+	C26H48O2	431.32840					1.5E+05	0.4						
**1186**	Dihydroxycampesterol	[M+H]+	C28H46O3	431.35277									9.0E+05	-1.9	5.1E+05	-2.2
**1187**	Undecylprodigiosin	[M+K]+	C25H35N3O	432.24049									2.0E+05	1.6		
**1188**	Veratramine	[M+Na]+	C27H39NO2	432.28730	9.8E+04	-1.3										
**1189**	Sphingofungin C	[M+H]+	C22H41NO7	432.29496							6.3E+05	1.4				
**1190**	Quercetin 3,7-dimethyl ether 4'-sulfate	[M+Na]+	C17H14O10S	433.01929											7.7E+04	1.6
**1191**	Geranyl-4,2',4'-trihydroxydihydrochalcone	[M+K]+	C25H30O4	433.17852					3.9E+05	-2.2						
**1192**	25-hydroxy-16,17,23,23,24,24-hexadehydrovitamin D3	[M+K]+	C27H38O2	433.25129									1.3E+05	-2.2	8.7E+04	-0.4
**1193**	1alpha,25-dihydroxy-24-oxo-22-oxavitamin D3	[M+H]+	C26H40O5	433.29421									2.5E+05	1.5		
**1194**	Vitamin D6	[M+Na]+	C29H46O	433.34309					2.2E+05	2.3						
**1195**	Dalpanol	[M+Na]+	C23H24O7	435.14130			1.8E+05	0.3								
**1196**	alpha,4,2'-Trihydroxy-4-O-geranyldihydrochalcor	[M+K]+	C24H28O5	435.15683	1.0E+05	-0.4										
**1197**	(9S,10S)-10-Hydroxy-9-(phosphonooxy)octadecanoate	[M+K]+	C18H37O7P	435.19131											1.2E+05	-1.1
**1198**	Deoxoteasterone	[M+H]+	C28H50O3	435.38327	8.3E+04	0.2										
**1199**	PC(4:0/4:0)	[M+K]+	C16H32NO8P	436.15005									1.1E+05	-0.8		

#	Putative Annotation [a]	Ion	Formula	$m/z_{Theo.}$ [b]	R2020[c]	ppm	W2020[c]	ppm	B2020[c]	ppm	R2021[c]	ppm	W2021[c]	ppm	B2021[c]	ppm
**1200**	(22E,24E)-1alpha,26-dihydroxy-22,23,24,25-tetradehydro-27-norvitamin D3	[M+K]+	C26H38O3	437.24483									3.4E+06	1.0		
**1201**	Tetracosanedioic acid	[M+K]+	C24H46O4	437.30324					5.2E+05	-1.1						
**1202**	Palmitoylcarnitine	[M+K]+	C23H45NO4	438.29860									2.2E+05	-1.3		
**1203**	Lupinisoflavone J	[M+K]+	C25H26O7	439.17513	1.8E+05	1.2										
**1204**	PA(18:0/0:0)	[M+H]+	C21H43O7P	439.28126							1.2E+05	1.5				
**1205**	LysoPE(0:0/15:0)	[M+H]+	C20H42NO7P	440.27652									1.9E+05	1.5		
**1206**	Carboxy-alpha-chromanol	[M+Na]+	C26H41O4	440.28960					2.2E+05	0.2						
**1207**	Kaempferol 3-arabinopyranoside	[M+Na]+	C20H18O10	441.07922	2.6E+05	0.5										
**1208**	Chalconaringenin 2'-xyloside	[M+K]+	C20H20O9	443.07389	1.7E+05	0.9										
**1209**	4,4'-Diapo-zeta-carotene	[M+K]+	C30H44	443.30731											1.1E+05	0.3
**1210**	Lepidissipyrone	[M+Na]+	C24H22O7	445.12677					2.2E+05	-2.2						
**1211**	1-Octen-3-ol-3-o-beta-D-xylopyranosyl(1->6)-beta-D-glucopyranoside	[M+Na]+	C19H34O10	445.20455									2.4E+05	-0.3		
**1212**	Quassin	[M+Na]+	C23H34O7	445.21955							1.2E+05	0.3				
**1213**	11alpha-ethyl-1alpha,25-dihydroxyvitamin D3	[M+H]+	C29H48O3	445.36841											6.5E+05	-1.8
**1214**	N-oleoyl tyrosine	[M+H]+	C27H43NO4	446.32564									2.7E+05	1.9		
**1215**	PA(16:1(9Z)/0:0)	[M+K]+	C19H37O7P	447.19178					3.1E+05	-2.1			1.0E+05	1.5		
**1216**	PA(8:0/8:0)	[M+Na]+	C19H37O8P	447.21137											1.3E+05	1.0
**1217**	2-Geranyl-3,4,2',4'-tetrahydroxydihydrochalcone	[M+K]+	C25H30O5	449.17164									1.2E+05	1.9		
**1218**	1alpha-hydroxy-24-methylvitamin D2	[M+Na]+	C29H46O2	449.33935					3.5E+05	-0.8						
**1219**	Catechin 3-O-(1-hydroxy-6-oxo-2-cyclohexene-1-carboxylate)	[M+Na]+	C22H20O9	451.09999					3.7E+05	-0.1						
**1220**	Tephrodin	[M+H]+	C25H22O8	451.13790											8.7E+04	1.9
**1221**	Cinchonain 1a	[M+H]+	C24H20O9	453.11835					5.1E+05	-0.8						
**1222**	2,2-Dimethyl-3,4-bis(4-methoxyphenyl)-2H-1-benzopyran-7-ol acetate	[M+Na]+	C27H26O5	453.16818									2.1E+05	-2.1		
**1223**	Oxo-leukotriene E4	[M+H]+	C23H34NO6S	453.21761									4.6E+05	0.8		
**1224**	MG(0:0/24:6(6Z,9Z,12Z,15Z,18Z,21Z)/0:0)	[M+Na]+	C27H42O4	453.29746											1.2E+05	0.2
**1225**	Phyllanthin	[M+K]+	C24H34O6	457.19957					5.7E+05	-1.9						
**1226**	Deoxotyphasterol	[M+Na]+	C28H50O3	457.36522	1.5E+05	-2.0							8.0E+05	1.5		
**1227**	N-(4-benzenesulfonamide) arachidonoyl amine	[M+H]+	C26H38N2O3S	459.26759	1.4E+05	0.7										
**1228**	Salvisyriacolide	[M+Na]+	C25H40O6	459.27091							1.8E+05	1.7				
**1229**	1-(3-methylpentadecyl)-3-nonylcyclopentane	[M+K]+	C30H60	459.43216											2.3E+05	1.1
**1230**	Militarinone A	[M+H]+	C26H37NO6	460.27030							9.6E+04	-2.0				
**1231**	N-stearoyl histidine	[M+K]+	C24H43N3O3	460.29344					1.9E+05	0.4						
**1232**	Apigenin 5,7-dimethyl ether 4'-galactoside	[M+H]+	C23H24O10	461.14461							9.5E+04	-0.8				
**1233**	PA(P-18:0/0:0)	[M+K]+	C21H43O6P	461.24344							2.1E+05	-1.2				
**1234**	Ophirasterol	[M+Na]+	C31H50O	461.37521					2.7E+05	0.4						

#	Putative Annotation [a]	Ion	Formula	$m/z_{Theo.}$ [b]	R2020[c]	ppm	W2020[c]	ppm	B2020[c]	ppm	R2021[c]	ppm	W2021[c]	ppm	B2021[c]	ppm
**1235**	Linoelaidyl carnitine	[M+K]+	C25H45NO4	462.29773									1.2E+05	0.6		
**1236**	Flemiflavanone D	[M+K]+	C25H28O6	463.15075			1.4E+05	2.2								
**1237**	Retinyl beta-glucuronide	[M+H]+	C26H38O7	463.26933											1.3E+05	-0.6
**1238**	PA(20:2(11Z,14Z)/0:0)	[M+H]+	C23H43O7P	463.28226							1.1E+05	-0.7				
**1239**	Pfaffic acid	[M+Na]+	C29H44O3	463.31861					3.8E+05	-0.7			2.4E+05	1.2		
**1240**	1-O-alpha-D-glucopyranosyl-1,2-nonadecandiol	[M+H]+	C25H50O7	463.36241					3.2E+05	1.1						
**1241**	N-arachidonoyl histidine	[M+Na]+	C26H39N3O3	464.28830			1.2E+05	0.1								
**1242**	PA(20:1(11Z)/0:0)	[M+H]+	C23H45O7P	465.29770					6.1E+05	-0.3						
**1243**	PGF2alpha-dihydroxypropanylamine	[M+K]+	C23H41NO6	466.25654											8.0E+04	0.0
**1244**	N-Adenylylanthranilate	[M+H]+	C17H19N6O8P	467.10673									2.5E+05	1.6		
**1245**	dihydrophaseic acid 4-O-beta-D-glucoside	[M+Na]+	C21H32O10	467.18870											1.0E+05	0.1
**1246**	Ercalcitriol	[M+K]+	C28H44O3	467.29219			1.5E+05	0.0								
**1247**	LysoSM(d18:0)	[M+H]+	C23H51N2O5P	467.36084	1.1E+05	0.6										
**1248**	Phthioceranic acid	[M+H]+	C31H62O2	467.48226	1.1E+05	-1.0										
**1249**	Apigenin 7-glucuronide	[M+Na]+	C21H18O11	469.07413	9.7E+04	-0.2										
**1250**	1alpha,25-dihydroxy-11-(3-hydroxy-1-propynyl)-9,11-didehydrovitamin D3	[M+H]+	C30H44O4	469.33062			8.2E+05	1.3								
**1251**	Vitamin E	[M+K]+	C29H50O2	469.34405					3.8E+05	0.4						
**1252**	Glutathionylspermidine	[M+K]+	C17H34N6O5S	473.19430	8.6E+04	1.9										
**1253**	Euchrenone a14	[M+H]+	C30H32O5	473.23267							1.2E+05	-0.9				
**1254**	PA(O-20:0/0:0)	[M+Na]+	C23H49O6P	475.31590	1.4E+05	-1.2										
**1255**	(20S)-1alpha,25-dihydroxy-20-methoxy-26,27-dimethylvitamin D3	[M+H]+	C30H50O4	475.37906											1.4E+05	-1.8
**1256**	N-docosahexaenoyl phenylalanine	[M+H]+	C31H41NO3	476.31676											4.3E+05	-1.8
**1257**	Rhodojaponin IV	[M+Na]+	C24H38O8	477.24574					4.3E+05	0.3						
**1258**	Moronic acid	[M+Na]+	C30H46O3	477.33379							1.7E+05	0.3				
**1259**	triacontan-1,14-diol	[M+Na]+	C30H62O2	477.46344											8.5E+04	1.6
**1260**	PS(13:0/0:0)	[M+Na]+	C19H38NO9P	478.21863									1.4E+05	-2.1		
**1261**	PA(20:5(5Z,8Z,11Z,14Z,17Z)/0:0)	[M+Na]+	C23H37O7P	479.21679					6.8E+05	0.3						
**1262**	Soyasapogenol C	[M+K]+	C30H48O2	479.32838									3.1E+05	0.4		
**1263**	Lythramine	[M+H]+	C29H37NO5	480.27445	1.0E+05	1.1										
**1264**	Exiguaflavanone M	[M+K]+	C25H30O7	481.16245			1.3E+05	-0.3								
**1265**	N-palmitoyl tryptophan	[M+K]+	C27H42N2O3	481.28207							4.4E+05	1.3				
**1266**	sn-3-O-(Geranylgeranyl)glycerol 1-phosphate	[M+K]+	C23H41O6P	483.22682									1.2E+05	0.9		
**1267**	1alpha,25-dihydroxy-24a,24b-didihomo-22-thia-20-epivitamin D3	[M+Na]+	C28H46O3S	485.30599	3.1E+05	1.9										
**1268**	S-Decyl GSH	[M+K]+	C20H37N3O6S	486.20347	9.1E+04	-1.1										
**1269**	Quercitrin	[M+K]+	C21H20O11	487.06372	1.3E+05	0.4										
**1270**	Glucosylgalactosyl hydroxylysine	[M+H]+	C18H34N2O13	487.21365							1.2E+05	-0.6				

#	Putative Annotation [a]	Ion	Formula	$m/z_{Theo.}$ [b]	R2020[c]	ppm	W2020[c]	ppm	B2020[c]	ppm	R2021[c]	ppm	W2021[c]	ppm	B2021[c]	ppm
**1271**	N-(2'-(4-benzenesulfonamide)-ethyl) arachidonoyl amine	[M+H]+	C28H42N2O3S	487.29889	4.0E+05	0.3										
**1272**	Fucosyllactose	[M+H]+	C18H32O15	489.18087			1.2E+05	1.1								
**1273**	Vitamin K1 epoxide	[M+Na]+	C31H46O3	489.33309					2.3E+05	1.7	1.6E+05	0.7				
**1274**	4'-Apo-3,4-didehydrolycopene	[M+Na]+	C35H46	489.34972											2.0E+05	-1.1
**1275**	alpha-Hydroxy-castasterone	[M+Na]+	C28H50O5	489.35470									8.9E+05	0.7		
**1276**	(17E)-1alpha,25-dihydroxy-26,27-dimethyl-17,20,22,22,23,23-hexadehydro-24a-homovitamin D3	[M+K]+	C30H44O3	491.29281											1.2E+05	-1.2
**1277**	PA(22:2(13Z,16Z)/0:0)	[M+H]+	C25H47O7P	491.31370									1.3E+05	-1.0		
**1278**	Palmatoside G	[M+H]+	C25H32O10	493.20683									1.6E+05	0.0		
**1279**	TG(8:0/8:0/8:0)	[M+Na]+	C27H50O6	493.35001					3.6E+05	-0.1						
**1280**	Eburicoic acid	[M+Na]+	C31H50O3	493.36514			1.8E+05	0.2								
**1281**	24R-Ethyl-26,26,27-trimethyl-3beta-hydroxycholesta-7,26-(30)-diene	[M+K]+	C32H54O	493.38043							1.3E+05	0.4				
**1282**	3,5,8,3',4'-Pentamethoxy-7-prenyloxyflavone	[M+K]+	C25H28O8	495.14252											8.2E+04	-1.9
**1283**	Atocalcitol	[M+H]+	C32H46O4	495.34665							1.1E+05	0.5				
**1284**	Deoxypumiloside	[M+H]+	C26H28N2O8	497.19234									1.7E+05	-1.0		
**1285**	Palmitoylglycerophosphocholine	[M+H]+	C24H51NO7P	497.34759	1.7E+05	0.6										
**1286**	Soyasapogenol A	[M+Na]+	C30H50O4	497.35975			1.6E+05	0.8								
**1287**	1alpha,25-dihydroxy-24a-homo-26,27-dimethyl-22-thiavitamin D3	[M+Na]+	C29H48O3S	499.32189							1.2E+05	-0.5				
**1288**	6-methylvitamin D3 6,19-sulfur dioxide adduct	[M+K]+	C28H46O3S	501.27967									2.8E+05	0.5		
**1289**	LysoPE(0:0/18:1(11Z))	[M+Na]+	C23H46NO7P	502.29130									1.4E+05	-1.8		
**1290**	Cycloheterophyllin	[M+H]+	C30H30O7	503.20574					2.8E+05	1.4						
**1291**	Castasterone	[M+K]+	C28H48O5	503.31323									2.6E+05	0.2		
**1292**	Hexaprenylphenol	[M+H]+	C36H54O	503.42541											2.5E+05	-1.3
**1293**	3,4,2',3',4',6',alpha-Heptahydroxychalcone 2'-glucoside	[M+Na]+	C21H22O13	505.09526	1.1E+05	0.2										
**1294**	11-beta-hydroxyandrosterone-3-glucuronide	[M+Na]+	C25H38O9	505.24041									1.2E+05	0.8		
**1295**	Eudesobovatol A	[M+H]+	C33H44O4	505.33212									2.2E+05	-1.8	1.2E+05	-1.7
**1296**	Methyl-triacontanoic acid	[M+K]+	C31H62O2	505.43757			1.5E+05	1.1								
**1297**	Artoindonesianin B	[M+K]+	C26H28O8	507.14152									1.3E+05	0.1		
**1298**	Stigmatellin X	[M+K]+	C28H38O6	509.23029					7.2E+05	-0.6						
**1299**	PA(20:4(5Z,8Z,11Z,14Z)e/2:0)	[M+Na]+	C25H43O7P	509.26303							1.4E+05	1.6				
**1300**	N-(2'-(4-benzenesulfonamide)-ethyl) arachidonoyl amine	[M+Na]+	C28H42N2O3S	509.27994			8.6E+05	1.8								
**1301**	PS(17:1(9Z)/0:0)	[M+H]+	C23H44NO9P	510.28265	2.7E+05	-0.6										
**1302**	PS(8:0/8:0)	[M+H]+	C22H42NO10P	512.26285			1.4E+05	-1.8								
**1303**	DG(14:0/14:0/0:0)	[M+H]+	C31H60O5	513.45084											1.6E+05	1.0

#	Putative Annotation [a]	Ion	Formula	m/z$_{Theo.}$ [b]	R2020[c]	ppm	W2020[c]	ppm	B2020[c]	ppm	R2021[c]	ppm	W2021[c]	ppm	B2021[c]	ppm
**1304**	N-docosahexaenoyl phenylalanine	[M+K]+	C31H41NO3	514.27230					2.7E+05	-1.0						
**1305**	Taurocholic acid	[M+H]+	C26H45NO7S	516.29925					1.9E+05	-0.6						
**1306**	Leukotriene D4	[M+Na]+	C25H40N2O6S	519.24952					3.0E+05	0.8						
**1307**	Linoleoylglycerophosphocholine	[M+H]+	C26H51NO7P	521.34825											1.5E+05	-1.3
**1308**	1alpha,22,25-trihydroxy-26,27-dimethyl-23,23,24,24-tetradehydro-24a,24b,24c-trihomovitamin D3	[M+Na]+	C32H50O4	521.35994									1.4E+05	0.4		
**1309**	Taurodeoxycholic acid	[M+Na]+	C26H45NO6S	522.28631					2.2E+05	-0.6						
**1310**	Frangulanine	[M+Na]+	C28H44N4O4	523.32530											8.7E+04	0.3
**1311**	Docosanediol-1,14-disulfate	[M+Na]+	C22H46O8S2	525.25263	1.9E+06	-1.7										
**1312**	N-(2'-(4-benzenesulfonamide)-ethyl) arachidonoyl amine	[M+K]+	C28H42N2O3S	525.25563							1.9E+05	-1.6				
**1313**	PG(19:1(9Z)/0:0)	[M+H]+	C25H49O9P	525.31929							3.4E+05	-1.1				
**1314**	Retinyl palmitate	[M+H]+	C36H60O2	525.46611											1.3E+05	0.9
**1315**	6-Hydroxykaempferol 3,5,7,4'-tetramethyl ether 6-rhamnoside	[M+Na]+	C25H28O11	527.15296											8.9E+04	-1.1
**1316**	PG(18:3(6Z,9Z,12Z)/0:0)	[M+Na]+	C24H43O9P	529.25369	1.1E+05	-1.2										
**1317**	Curcumin monoglucoside	[M+H]+	C27H30O11	531.18596			1.4E+05	0.2								
**1318**	Caryoptin	[M+K]+	C26H36O9	531.19922											1.2E+05	-0.2
**1319**	PI(13:0/0:0)	[M+H]+	C22H43O12P	531.25632											1.4E+05	0.3
**1320**	Uscharidin	[M+H]+	C29H38O9	531.25956			3.7E+05	-1.3								
**1321**	PG(18:2(9Z,12Z)/0:0)	[M+Na]+	C24H45O9P	531.26936									4.9E+05	0.0		
**1322**	LysoPC(18:4(6Z,9Z,12Z,15Z))	[M+Na]+	C26H46NO7P	538.28948											9.7E+04	1.7
**1323**	zeta-Carotene	[M+H]+	C40H60	541.47656											1.6E+05	0.4
**1324**	PC(O-16:1(11Z)/2:0)	[M+Na]+	C26H52NO7P	544.33736	3.9E+05	-1.4										
**1325**	Quercetin 3-(3''-sulfatoglucoside)	[M+H]+	C21H20O15S	545.05992			1.4E+05	-0.6								
**1326**	Phytoene	[M+H]+	C40H64	545.50808	2.4E+05	0.2										
**1327**	Guanosine triphosphate	[M+Na]+	C10H16N5O14P3	545.98084											2.5E+05	-1.8
**1328**	Hirsutidin 3-glucoside	[M+K]+	C24H27O12	546.11317									4.3E+05	0.4		
**1329**	Deacetylipecoside	[M+Na]+	C25H33NO11	546.19426							2.0E+05	0.6				
**1330**	PG(P-20:0/0:0)	[M+Na]+	C26H53O8P	547.33686									2.9E+05	0.3		
**1331**	N3'-Acetyltobramycin	[M+K]+	C20H39N5O10	548.23285	1.6E+05	-1.5										
**1332**	PS(20:3(8Z,11Z,14Z)/0:0)	[M+H]+	C26H46NO9P	548.29731					1.9E+05	1.8						
**1333**	Cholesteryl-beta-D-glucoside	[M+H]+	C33H56O6	549.41497	2.1E+05	-1.1										
**1334**	PC(16:0/3:1(2E))	[M+H]+	C27H52NO8P	550.35105									1.4E+05	-1.3		
**1335**	L-Oleandrosyl-oleandolide	[M+Na]+	C27H46O10	553.29762					6.6E+05	1.3						
**1336**	6,8a-Seco-6,8a-deoxy-5-oxoavermectin ''1b'' aglycone	[M+H]+	C33H46O7	555.33163	5.2E+05	0.7										
**1337**	Cucurbitacin F	[M+K]+	C30H46O7	557.28838					4.5E+05	-1.6						
**1338**	PC(O-16:0/2:0)	[M+K]+	C26H54NO7P	562.32709					1.8E+05	-0.3						

#	Putative Annotation [a]	Ion	Formula	$m/z_{Theo.}$ [b]	R2020[c]	ppm	W2020[c]	ppm	B2020[c]	ppm	R2021[c]	ppm	W2021[c]	ppm	B2021[c]	ppm
**1339**	Cortolone-3-glucuronide	[M+Na]+	C27H42O11	565.26267					4.1E+05	-1.3						
**1340**	dTDP-L-oleandrose	[M+Na]+	C17H28N2O14P2	569.09121			1.2E+05	-0.7								
**1341**	Sarmentoloside	[M+H]+	C29H44O11	569.29595					6.7E+05	-0.5						
**1342**	12alpha-Hydroxyamoorstatin	[M+K]+	C28H36O10	571.19407			1.3E+05	-0.1								
**1343**	2-Hexaprenyl-6-methoxyphenol	[M+K]+	C37H56O2	571.39211			1.4E+05	-1.6								
**1344**	Adonitoxigenin 3-O-alpha-L-rhamnoside	[M+Na]+	C29H42O10	573.26772											1.5E+05	-1.2
**1345**	PI(16:0/0:0)	[M+H]+	C25H49O12P	573.30344	1.5E+05	-0.2										
**1346**	beta-Carotene	[M+K]+	C40H56	575.40210									5.2E+05	-1.3		
**1347**	PA(P-16:0/12:0)	[M+H]+	C31H61O7P	577.42196									7.1E+05	1.4		
**1348**	Cucurbitacin E	[M+Na]+	C32H44O8	579.29225											1.1E+05	1.0
**1349**	Prorepensin	[M+K]+	C35H44O5	583.28185					5.0E+05	0.3						
**1350**	Ouabain	[M+H]+	C29H44O12	585.29104					4.8E+05	-0.8						
**1351**	Apigenin 7-alpha-L-arabinofuranosyl-(1->6)-glucoside	[M+Na]+	C26H28O14	587.13740											1.3E+05	-0.5
**1352**	PC(10:0/10:0)	[M+Na]+	C28H56NO8P	588.36358	3.7E+05	0.9										
**1353**	PC(O-16:0/4:0)	[M+K]+	C28H58NO7P	590.35908					1.8E+05	-1.4						
**1354**	Lithocholate 3-O-glucuronide	[M+K]+	C30H48O9	591.29398									2.8E+05	-1.7		
**1355**	Spheroidene	[M+Na]+	C41H60O	591.45291							2.7E+05	1.2				
**1356**	Agavoside A	[M+H]+	C33H52O9	593.36912											1.5E+05	-1.2
**1357**	PI(P-16:0/0:0);	[M+K]+	C25H49O11P	595.26456											9.9E+04	-0.3
**1358**	7,8-Didehydroastaxanthin	[M+H]+	C40H50O4	595.37800											9.7E+04	0.3
**1359**	PG(22:4(7Z,10Z,13Z,16Z)/0:0)	[M+K]+	C28H49O9P	599.27363											1.3E+05	1.6
**1360**	PI(18:0/0:0)	[M+H]+	C27H53O12P	601.33412					2.7E+05	1.0						
**1361**	Quercetin 3-rhamnosyl-(1->2)-alpha-L-arabinopyranoside	[M+Na]+	C26H28O15	603.13180					1.5E+05	0.4						
**1362**	alpha,alpha'-Trehalose 6-palmitate	[M+Na]+	C28H52O12	603.33569			1.4E+05	-1.0					4.7E+05	-0.7		
**1363**	1-(O-alpha-D-glucopyranosyl)-3-keto-(1,27R)-octacosanediol	[M+H]+	C34H66O8	603.48262									4.3E+05	0.7		
**1364**	5-Oxoavermectin ''1a'' aglycone	[M+Na]+	C34H46O8	605.30813									3.2E+05	0.6		
**1365**	Avermectin B2b aglycone	[M+Na]+	C33H48O9	611.31880					5.4E+05	0.4						
**1366**	PC(18:1(9Z)/4:0)	[M+Na]+	C30H58NO8P	614.37959									2.6E+05	-0.6		
**1367**	Cytidine monophosphate N-acetylneuraminic acid	[M+H]+	C20H31N4O16P	615.15453			2.7E+05	0.0								
**1368**	PA(P-16:0/12:0)	[M+K]+	C31H61O7P	615.37823											1.1E+05	0.7
**1369**	CE(14:1(9Z))	[M+Na]+	C41H70O2	617.52603									1.7E+05	1.3		
**1370**	Catechin 3-O-rutinoside	[M+Na]+	C27H34O15	621.17899	1.8E+05	1.5										
**1371**	Dihydrospirilloxanthin	[M+Na]+	C42H62O2	621.46388							3.5E+05	0.5				
**1372**	Rodiasine	[M+H]+	C38H42N2O6	623.31090					5.5E+05	1.1						
**1373**	Leukotriene C4	[M+H]+	C30H47N3O9S	626.30959					2.1E+05	1.6						
**1374**	Agavoside A	[M+K]+	C33H52O9	631.32479					2.7E+05	-0.8						

#	Putative Annotation [a]	Ion	Formula	$m/z_{Theo.}$ [b]	R2020[c]	ppm	W2020[c]	ppm	B2020[c]	ppm	R2021[c]	ppm	W2021[c]	ppm	B2021[c]	ppm
**1375**	DG(14:0/20:2(11Z,14Z)/0:0)	[M+K]+	C37H68O5	631.46994			1.5E+05	-0.2								
**1376**	PA(12:0/19:0)	[M+H]+	C34H67O8P	635.46528											2.3E+05	-1.0
**1377**	Ansatrienin A	[M+H]+	C36H48N2O8	637.34786					4.0E+05	0.8						
**1378**	PG(12:0/14:1(9Z))	[M+H]+	C32H61O10P	637.40751	1.5E+05	0.7										
**1379**	dolichyl beta-D-glucosyl phosphate	[M+K]+	C31H55O9P	641.32115					4.5E+05	0.6						
**1380**	Pentacosanyl oleate	[M+K]+	C41H80O2	643.57905							5.9E+05	-0.1				
**1381**	Rhizochalinin D	[M+H]+	C35H70N2O8	647.52100									5.1E+05	-0.8		
**1382**	5-O-Methyleriodictyol 7-glucosyl-(1->4)-galactoside	[M+Na]+	C28H34O16	649.17469			1.4E+05	-1.2								
**1383**	PI(20:1(11Z)/0:0)	[M+Na]+	C29H55O12P	649.33220					3.3E+05	0.2						
**1384**	PE(14:0/15:0)	[M+H]+	C34H68NO8P	650.47592							2.0E+05	-0.6				
**1385**	DG(17:2(9Z,12Z)/22:6(4Z,7Z,10Z,13Z,16Z,19Z)/0:0)	[M+H]+	C42H66O5	651.49923			1.8E+05	-1.4								
**1386**	Hydroxymytiloxanthin	[M+K]+	C40H54O5	653.36084									2.6E+05	-0.9		
**1387**	Hirsutidin 3-O-(6-O-p-coumaroyl)glucoside	[M+H]+	C33H33O14	654.19431	1.3E+05	0.3										
**1388**	Cer(d18:1/22:1(13Z))	[M+K]+	C40H77NO3	658.55417											2.9E+05	-1.0
**1389**	Peonidin 3-(6''-caffeylgucoside)	[M+K]+	C31H29O14	664.11810									1.3E+05	1.2		
**1390**	PI(21:0/0:0)	[M+Na]+	C30H59O12P	665.36434									2.0E+05	-1.1		
**1391**	PA(12:0/20:2(11Z,14Z))	[M+Na]+	C35H65O8P	667.42993							1.4E+05	1.5				
**1392**	DG(15:0/22:4(7Z,10Z,13Z,16Z)/0:0)	[M+K]+	C40H70O5	669.48461			1.3E+05	1.3								
**1393**	SM(d18:0/12:0)	[M+Na]+	C35H73N2O6P	671.50988			1.5E+05	-0.1								
**1394**	Decaprenoxanthin	[M+H]+	C50H72	673.57059											1.6E+05	0.1
**1395**	CerP(d18:1/20:0)	[M+H]+	C38H76NO6P	674.54820									3.1E+05	0.2		
**1396**	Octaprenylcatechol	[M+Na]+	C46H70O2	677.52696									8.0E+05	-0.2		
**1397**	PS(O-16:0/13:0)	[M+H]+	C35H70NO9P	680.48553			1.2E+06	0.8								
**1398**	DG(16:0/22:5(4Z,7Z,10Z,13Z,16Z)/0:0)	[M+K]+	C41H70O5	681.48561			6.0E+05	-0.2								
**1399**	PA(O-20:0/14:1(9Z))	[M+Na]+	C37H73O7P	683.49791									1.7E+05	1.0		
**1400**	Cyanidin 3-(6''-malonylsambubioside)	[M+Na]+	C29H31O18	690.14065			1.4E+05	-0.6								
**1401**	Diphytanyl-sn-glycerol	[M+K]+	C43H88O3	691.63600			1.7E+05	0.7								
**1402**	Octaprenylcatechol	[M+K]+	C46H70O2	693.50086					2.1E+05	-0.2						
**1403**	PC(10:0/18:2(9Z,12Z))	[M+Na]+	C36H68NO8P	696.45798											1.3E+05	-0.7
**1404**	PS(14:1(9Z)/14:1(9Z))	[M+Na]+	C34H62NO10P	698.40080			1.8E+05	-0.6								
**1405**	PE(14:0/16:1(9Z))	[M+K]+	C35H68NO8P	700.43122					1.3E+05	0.3						
**1406**	PA(13:0/22:6(4Z,7Z,10Z,13Z,16Z,19Z))	[M+Na]+	C38H63O8P	701.41607											1.2E+05	-1.1
**1407**	1-Palmitoyl-2-(5-keto-6-octendioyl)-sn-glycero-3-phosphatidylcholine	[M+K]+	C32H58NO11P	702.33696					1.1E+05	1.3						
**1408**	PS(P-16:0/12:0)	[M+K]+	C34H66NO9P	702.41006			1.8E+05	0.9								
**1409**	Anthenoside A	[M+Na]+	C38H65NO9	702.45446									1.6E+05	1.0		
**1410**	Bruceoside A	[M+Na]+	C32H42O16	705.23585									1.3E+05	0.9		
**1411**	PA(15:0/20:3(8Z,11Z,14Z))	[M+Na]+	C38H69O8P	707.46170					1.6E+05	0.7						

#	Putative Annotation [a]	Ion	Formula	$m/z_{Theo.}$ [b]	R2020[c]	ppm	W2020[c]	ppm	B2020[c]	ppm	R2021[c]	ppm	W2021[c]	ppm	B2021[c]	ppm
**1412**	Kaempferol 3-(6''-sulfatogetiobioside)	[M+Na]+	C27H30O19S	713.09959					1.2E+05	-0.2						
**1413**	DG(21:0/22:5(7Z,10Z,13Z,16Z,19Z)/0:0)	[M+H]+	C46H80O5	713.60740											1.0E+05	0.6
**1414**	PE(14:0/20:2(11Z,14Z))	[M+H]+	C39H74NO8P	716.52248	1.5E+05	-1.2										
**1415**	CE(20:1(11Z))	[M+K]+	C47H82O2	717.59518											2.2E+05	-0.8
**1416**	2',4'-Dihydroxy-6'-methoxy-3'-(2-hydroxybenzyl)-5'-(3 x 2-hydroxybenzyl)dihydrochalcone	[M+Na]+	C44H40O8	719.26239					1.4E+05	-1.2						
**1417**	PA(O-18:0/18:4(6Z,9Z,12Z,15Z))	[M+K]+	C39H71O7P	721.45623							2.3E+05	0.9				
**1418**	PE(18:2(9Z,12Z)/P-18:1(11Z))	[M+H]+	C41H76NO7P	726.54277			2.1E+05	0.6								
**1419**	Chivosazole F	[M+K]+	C41H57NO8	730.37070									1.3E+05	1.2		
**1420**	Diguanosine diphosphate	[M+Na]+	C20H26N10O15P2	731.09465	1.3E+05	1.2										
**1421**	Ubiquinol 8	[M+H]+	C49H78O4	731.59798					1.0E+05	-0.9						
**1422**	PE(15:0/18:4(6Z,9Z,12Z,15Z))	[M+K]+	C38H68NO8P	736.43215					1.2E+05	-1.0						
**1423**	PG(O-20:0/14:0)	[M+H]+	C40H81O9P	737.56969			2.0E+05	-0.8								
**1424**	PA(15:0/22:1(11Z))	[M+Na]+	C40H77O8P	739.52510											1.5E+05	-0.4
**1425**	PA(17:1(9Z))	[M+H]+	C42H77O8P	741.54296									1.3E+05	-0.1		
**1426**	PI(12:0/14:1(9Z))	[M+Na]+	C35H65O13P	747.40624									1.6E+05	-1.0		
**1427**	PA(O-20:0/20:0)	[M+H]+	C43H87O7P	747.62622	9.1E+04	0.0										
**1428**	PC(o-16:0/18:0)	[M+H]+	C42H86NO7P	748.62215									1.3E+05	-0.9		
**1429**	Quercetin 3-(2'''-galloylglucosyl)-(1->2)-alpha-L-arabinofuranoside	[M+H]+	C33H32O20	749.15558					1.5E+05	0.5						
**1430**	PG(O-18:0/17:0)	[M+H]+	C41H83O9P	751.58485							3.0E+05	-0.1				
**1431**	PE(20:2(11Z,14Z))	[M+H]+	C43H80NO7P	754.57533									1.2E+05	-1.1		
**1432**	DG(22:4(7Z,10Z,13Z,16Z)/24:1(15Z)/0:0)	[M+H]+	C49H86O5	755.65443							1.5E+05	0.5				
**1433**	PC(10:0/22:0)	[M+Na]+	C40H80NO8P	756.55091											1.1E+05	0.6
**1434**	PC(14:0/20:1(11Z))	[M+H]+	C42H82NO8P	760.58508	1.7E+05	-0.8										
**1435**	Ophiopogonin B	[M+K]+	C39H62O12	761.38729	1.7E+05	0.7										
**1436**	Malvidin 3-O-(6-O-(4-O-malonyl-alpha-rhamnopyranosyl)-beta-glucopyranoside	[M+K]+	C32H37O19	764.15606	8.4E+05	-1.0										
**1437**	Lampranthin II	[M+K]+	C34H34N2O16	765.15436											1.5E+05	-0.5
**1438**	PG(P-16:0/18:4(6Z,9Z,12Z,15Z))	[M+K]+	C40H71O9P	765.44669					1.2E+05	0.1						
**1439**	Quercetin 3-(2'''-galloylglucosyl)-(1->2)-alpha-L-arabinofuranoside	[M+Na]+	C33H32O20	771.13789					8.3E+04	0.0						
**1440**	Myxochromide S3	[M+Na]+	C40H56N6O8	771.40430					1.2E+05	1.1						
**1441**	PA(17:2(9Z,12Z))	[M+K]+	C42H71O8P	773.45132									1.6E+05	0.6		
**1442**	Loropetalin D	[M+Na]+	C35H28O19	775.11186					6.2E+04	-0.2						
**1443**	PI(12:0/18:4(6Z,9Z,12Z,15Z)	[M+H]+	C39H67O13P	775.43844									1.3E+05	1.0		
**1444**	2-Methyl-6-solanyl-1,4-benzoquinol	[M+K]+	C52H80O2	775.57968					5.8E+04	-0.9						
**1445**	PG(14:0/20:5(5Z,8Z,11Z,14Z,17Z))	[M+K]+	C40H69O10P	779.42646											1.5E+05	-0.6
**1446**	PC(14:0/20:1(11Z))	[M+Na]+	C42H82NO8P	782.56703	6.1E+05	-0.1										

#	Putative Annotation [a]	Ion	Formula	m/z$_{Theo.}$ [b]	R2020[c]	ppm	W2020[c]	ppm	B2020[c]	ppm	R2021[c]	ppm	W2021[c]	ppm	B2021[c]	ppm
**1447**	PA(O-18:0/22:1(11Z))	[M+K]+	C43H85O7P	783.56652											1.1E+05	-0.1
**1448**	PC(O-16:0/19:0)	[M+Na]+	C43H88NO7P	784.61933											1.2E+05	-0.3
**1449**	PA(20:0/22:1(11Z))	[M+H]+	C45H87O8P	787.62147									1.4E+05	-0.4		
**1450**	PA(20:4)/(22:6))	[M+Na]+	C45H69O8P	791.46314											9.6E+04	-1.2
**1451**	Quercetin 7-methyl ether 3-[3-hydroxy-3-methylglutaryl-(1->6)]-[apiosyl-(1->2)-galactoside]	[M+K]+	C33H38O20	793.15866									1.3E+05	0.2		
**1452**	PG(15:0/20:3(8Z,11Z,14Z))	[M+K]+	C41H75O10P	797.47294	1.5E+05	0.8										
**1453**	MGDG(18:2(9Z,12Z)/18:3(9Z,12Z,15Z))	[M+Na]+	C45H76O10	799.53266											8.8E+04	0.5
**1454**	PS(15:0/20:3(8Z,11Z,14Z))	[M+K]+	C41H74NO10P	810.46737							1.4E+05	1.0				
**1455**	GalCer(d18:2/23:0)	[M+Na]+	C47H89NO8	818.64804	1.2E+05	1.2										
**1456**	Thalicoside A	[M+Na]+	C42H70O14	821.46641									1.1E+05	-0.8		
**1457**	GalCer(d18:0/22:0)	[M+K]+	C46H91NO8	824.63808											1.7E+05	-0.5
**1458**	Yiamoloside B	[M+H]+	C43H68O15	825.46292									1.8E+05	0.2		
**1459**	PA(21:0/22:0)	[M+Na]+	C46H91O8P	825.63437							1.9E+05	0.0				
**1460**	TG(16:0/16:1(9Z)/16:1(9Z))	[M+Na]+	C51H94O6	825.69420			6.6E+05	0.1								
**1461**	TG(16:1(9Z)/16:1(9Z)/16:1(9Z))	[M+K]+	C51H92O6	839.65286									1.5E+05	-0.4		
**1462**	PG(17:2(9Z,12Z)/22:6(4Z,7Z,10Z,13Z,16Z,19Z)	[M+K]+	C45H73O10P	843.45784									1.7E+05	-0.7		
**1463**	Acetyl coenzyme A	[M+K]+	C23H38N7O17P3S	848.08874									1.0E+05	0.2		
**1464**	TG(16:1(9Z)/16:1(9Z)/20:5(5Z,8Z,11Z,14Z,17Z))	[M+H]+	C55H92O6	849.69590											1.6E+05	0.9
**1465**	Chrysoeriol 7-(2'''-feruloylglucuronosyl)-(1->2)-glucuronide	[M+Na]+	C38H36O21	851.16422									1.1E+05	-0.1		
**1466**	PG(O-18:0/22:1(11Z))	[M+K]+	C46H91O9P	857.60291					5.2E+04	0.4						
**1467**	PI(O-16:0/19:1(9Z))	[M+Na]+	C44H85O12P	859.56732							1.1E+05	-0.3				
**1468**	TG(17:2(9Z,12Z))	[M+Na]+	C54H92O6	859.67769									9.5E+04	1.1		
**1469**	PI(16:0/20:2(11Z,14Z))	[M+H]+	C45H83O13P	863.56377							1.2E+05	0.7				
**1470**	PG(20:4(5Z,8Z,11Z,14Z))	[M+Na]+	C48H75O10P	865.49899									1.1E+05	0.0		
**1471**	PG(22:4(7Z,10Z,13Z,16Z))	[M+Na]+	C48H77O10P	867.51495									1.4E+05	-0.3		
**1472**	TG(17:1(9Z)/18:1(9Z)/18:1(9Z))	[M+H]+	C56H102O6	871.77588							1.5E+05	-1.1				
**1473**	PG(19:1(9Z)/22:2(13Z,16Z))	[M+K]+	C47H87O10P	881.56685	1.1E+05	-0.7										
**1474**	TG(16:0/18:1(9Z)/20:4(5Z,8Z,11Z,14Z))	[M+H]+	C57H100O6	881.75927	1.8E+05	0.5	1.9E+06	-0.7								
**1475**	PI(16:0/20:2(11Z,14Z))	[M+Na]+	C45H83O13P	885.54568									9.7E+04	0.8		
**1476**	PG(22:6(4Z,7Z,10Z,13Z,16Z,19Z))	[M+Na]+	C50H79O10P	893.53108									1.3E+05	-0.9		
**1477**	TG(17:2(9Z,12Z)/18:0/18:0)	[M+Na]+	C56H104O6	895.77239									8.1E+04	0.1		
**1478**	TG(18:3(9Z,12Z,15Z))	[M+H]+	C59H94O6	899.71292									8.5E+04	-0.7		
**1479**	TG(17:1/20:5/20:5)	[M+H]+	C60H94O6	911.71274											1.5E+05	-0.5
**1480**	3-O-Sulfogalactosylceramide	[M+Na]+	C48H91NO11S	912.62065					5.5E+04	-0.2						
**1481**	TG(16:0/18:1/20:4)	[M+K]+	C57H100O6	919.71482			3.1E+06	0.4								
**1482**	PC(22:4/24:0)	[M+H]+	C54H100NO8P	922.72690			1.4E+06	-1.0								
**1483**	Cassiaside B2	[M+Na]+	C39H52O25	943.26946					7.1E+04	-0.5						

#	Putative Annotation [a]	Ion	Formula	$m/z_{Theo.}$ [b]	R2020[c]	ppm	W2020[c]	ppm	B2020[c]	ppm	R2021[c]	ppm	W2021[c]	ppm	B2021[c]	ppm
**1484**	Delphinidin 3,3'-di-glucoside-5-(6-caffeoylglucoside)	[M+H]+	C42H47O25	952.24853					5.7E+04	-0.6						
**1485**	PI(19:0/22:1(11Z))	[M+Na]+	C50H95O13P	957.64122					5.7E+04	-1.0						
**1486**	TG(17:2/20:5/22:4)	[M+Na]+	C62H98O6	961.72556	1.4E+05	0.3										
**1487**	TG(18:2(9Z,12Z)/20:1(11Z)/20:4(5Z,8Z,11Z,14Z))	[M+K]+	C61H104O6	971.74713			2.1E+05	-0.7								
**1488**	1-O-[(6'-O-hexadecanoyl)-a-D-glucopyranosyl]-(2-hexadecanoyloxy)-eicosan-1-ol	[M+Na]+	C58H112O9	975.81998			1.5E+05	-0.1								
**1489**	Malvidin 3-O-(6-O-(4-O-feruloyl-alpha-rhamnopyranosyl)-beta-glucopyranoside)-5-beta-glucopyranoside	[M+H]+	C45H53O24	978.30085							8.4E+04	-0.9				
**1490**	D-Mannosylphosphodecaprenol	[M+K]+	C56H93O9P	979.61813					2.0E+05	0.8						
**1491**	Magnoloside C	[M+K]+	C41H56O25	987.27493							9.3E+04	-0.7				

[a] CE Cholesteryl ester; Cer Ceramide; Galcer Galactosylceramide; DG Diacylglycerol; MG Monoacylglycerol; MGDG Monogalactosyldiacylglycerol; PA Phosphatidic acid; PC Phosphatidylcholine; PE Phosphatidylethanolamine; PG Glycerophospholipids; PGF2 Prostaglandin F2; PI Phosphatidylinositol; PS Phosphatidylserine; SM Sphingomyelin; TG Triacylglycerol.

[b] Theoretical mass

[c] R stands for red tunica, W stands for white tunica and B stands for bulb.

Table S7: Untargeted metabolic profiling of *Sulmona red garlic* hydroalcoholic extracts by ESI(-) FT-ICR MS

#	Putative Annotation [a]	Ion	Formula	m/z $_{Theo.}$ [b]	R2020 [c]	ppm	W2020 [c]	ppm	B2020 [c]	ppm	R2021 [c]	ppm	W2021 [c]	ppm	B2021 [c]	ppm
1	Pyruvic acid	[M-H]-	C3H4O3	87.00812					3.4E+04	4.7						
2	Butyric acid	[M-H]-	C4H8O2	87.04546											6.9E+04	-3.5
3	Pentanol	[M-H]-	C5H12O	87.08154	6.3E+04	3.7										
4	2-Propyn-1-al	[M+Cl]-	C3H2O	88.98055					3.7E+04	-2.1						
5	Propanethial S-oxide	[M-H]-	C3H6OS	89.00663					2.9E+04	0.3						
6	1,3-Butadiene	[M+Cl]-	C4H6	89.01609					2.9E+04	2.9						
7	Diethyl sulfide	[M-H]-	C4H10S	89.04343											8.1E+04	-4.3
8	3-Aminopropane-1,2-diol	[M-H]-	C3H9NO2	90.05639							5.0E+04	-3.8				
9	Thioglycolate	[M-H]-	C2H4O2S	90.98537					2.9E+04	1.6						
10	Dimethyl sulfone	[M-H]-	C2H6O2S	93.00157	6.2E+04	1.7									8.1E+04	0.8
11	Thiocyanate	[M+Cl]-	CHNS	93.95237			8.2E+04	0.9								
12	Hexadienal	[M-H]-	C6H8O	95.05029					4.1E+04	-0.5						
13	Carbamic acid	[M+Cl]-	CH3NO2	95.98567									5.3E+04	1.2		
14	Maleimide	[M-H]-	C4H3NO2	96.00910			8.2E+04	2.9								
15	Ethanolamine	[M+Cl]-	C2H7NO	96.02217			4.6E+04	-2.8								
16	Beta-vinyl acrylic acid	[M-H]-	C5H6O2	97.02883					3.0E+04	4.8						
17	Succinimide	[M-H]-	C4H5NO2	98.02475			9.3E+04	2.8								
18	Piperidinone	[M-H]-	C5H9NO	98.06144					3.2E+04	-3.1						
19	Tiglic acid	[M-H]-	C5H8O2	99.04515	7.0E+04	2.6									8.3E+04	4.0
20	Aminocyclopropane-carboxylate	[M-H]-	C4H7NO2	100.04040	5.4E+04	-2.2										
21	Succinate semialdehyde	[M-H]-	C4H6O3	101.02442	1.2E+05	-0.5			6.8E+04	-4.8					1.2E+05	4.6
22	Betaine aldehyde	[M-H]-	C5H12NO	101.08511									5.6E+04	-4.9		
23	Imidazole	[M+Cl]-	C3H4N2	103.00694											6.6E+04	-0.9
24	Choline	[M-H]-	C5H14NO	103.10052									3.4E+04	-2.5		
25	S-Methylthioglycolate	[M-H]-	C3H6O2S	105.00119							4.7E+04	3.7				
26	Glyceric acid	[M-H]-	C3H6O4	105.01976							4.1E+04	-4.1				
27	N-Methylaniline	[M-H]-	C7H9N	106.06652					3.2E+04	-2.8						
28	2-Methylpropanamine	[M+Cl]-	C4H11N	108.05855			3.5E+04	0.5								
29	Furoic acid	[M-H]-	C5H4O3	111.00877	7.4E+04	4.3										
30	Allyl sulfide	[M-H]-	C6H10S	113.04358							4.5E+04	-4.7				
31	Octane	[M-H]-	C8H18	113.13405					2.9E+04	-4.2						
32	Pyridine	[M+Cl]-	C5H5N	114.01204					2.4E+04	-3.9						
33	Methylthiopropionic acid	[M-H]-	C4H8O2S	119.01745											5.5E+04	-1.9
34	3-Methyl-2-butenal	[M+Cl]-	C5H8O	119.02746							3.0E+04	-4.6				
35	D-Erythrose	[M-H]-	C4H8O4	119.03498			4.0E+04	2.1								
36	Purine	[M-H]-	C5H4N4	119.03599									6.6E+04	2.8		
37	Cysteine	[M-H]-	C3H7NO2S	120.01247	4.9E+04	-4.8										
38	Piperidine	[M+Cl]-	C5H11N	120.05881											3.9E+04	-2.2
39	1,4-Lactone	[M+Cl]-	C4H6O2	121.00618	5.0E+04	-4.3										

#	Putative Annotation [a]	Ion	Formula	$m/z_{Theo.}$ [b]	R2020 [c]	ppm	W2020 [c]	ppm	B2020 [c]	ppm	R2021 [c]	ppm	W2021 [c]	ppm	B2021 [c]	ppm
**40**	Nicotinamide	[M-H]-	C6H6N2O	121.04046					3.8E+04	2.3						
**41**	Sulfoacetaldehyde	[M-H]-	C2H4O4S	122.97575	5.9E+04	0.6										
**42**	Selenocysteine	[M+Cl]-	C3H6NO2Se	123.00894											9.0E+05	2.6
**43**	2,4-octadienal	[M-H]-	C8H12O	123.08154	7.5E+04	-1.6										
**44**	Taurine	[M-H]-	C2H7NO3S	124.00739			5.3E+04	2.0								
**45**	Maltol	[M-H]-	C6H6O3	125.02375							4.2E+04	3.7				
**46**	Dihydrophloroglucinol	[M-H]-	C6H8O3	127.03966							2.7E+04	3.2				
**47**	L-Lysine 1,6-lactam	[M-H]-	C6H12N2O	127.08775											4.8E+04	-0.5
**48**	Aniline	[M+Cl]-	C6H7N	128.02733							3.3E+04	-0.6				
**49**	Guanidinobutanal	[M-H]-	C5H11N3O	128.08251					2.9E+04	3.3						
**50**	Octylamine	[M-H]-	C8H19N	128.14381					4.1E+04	4.8						
**51**	Dimethyl valeric acid	[M-H]-	C7H14O2	129.09293									4.2E+04	-3.1		
**52**	2-Hydroxypyridine	[M+Cl]-	C5H5NO	130.00652			5.9E+04	-1.5								
**53**	Hydroxy-L-proline	[M-H]-	C5H9NO3	130.05097	6.7E+04	0.3										
**54**	4-Guanidino-1-butanol	[M-H]-	C5H13N3O	130.09763					4.9E+04	4.8						
**55**	Furfural	[M+Cl]-	C5H4O2	130.99053	3.8E+04	-1.5										
**56**	Oxalacetic acid	[M-H]-	C4H4O5	130.99856											3.7E+04	0.3
**57**	Pteridine	[M-H]-	C6H4N4	131.03613							2.4E+04	1.5	5.2E+04	-3.7		
**58**	Asparagine	[M-H]-	C4H8N2O3	131.04622			8.0E+04	2.4								
**59**	Aspartic acid	[M-H]-	C4H7NO4	132.03023	3.4E+04	-3.0										
**60**	Ureidoglycine	[M-H]-	C3H7N3O3	132.04232											4.9E+04	-2.7
**61**	Hydroxyindole	[M-H]-	C8H7NO	132.04580					2.7E+04	-2.4						
**62**	Malic acid	[M-H]-	C4H6O5	133.01389							3.5E+04	2.7				
**63**	Imidazole-4-methanol	[M+Cl]-	C4H6N2O	133.01672									1.3E+05	5.2		
**64**	Cinnamyl alcohol	[M-H]-	C9H10O	133.06589			9.7E+04	0.3								
**65**	2-Phenylacetamide	[M-H]-	C8H9NO	134.06114			5.8E+04	-1.0								
**66**	Caproaldehyde	[M+Cl]-	C6H12O	135.05822	5.0E+04	2.5										
**67**	Hydroxypurine	[M-H]-	C5H6N4O	137.04627					3.7E+04	4.5						
**68**	Hexan-1-ol	[M+Cl]-	C6H14O	137.07387	5.0E+04	0.3										
**69**	Cadaverine	[M+Cl]-	C5H14N2	137.08433									3.8E+04	2.7		
**70**	Malonic acid	[M+Cl]-	C3H4O4	138.98036	5.2E+04	4.5										
**71**	5-Phenyl-1,3-pentadiyne	[M-H]-	C11H8	139.05589					4.9E+04	-4.1						
**72**	Methylthiopropanamine	[M+Cl]-	C4H11NS	140.03090									3.2E+04	-2.0		
**73**	Muconic acid	[M-H]-	C6H6O4	141.01869									3.3E+04	4.6		
**74**	4-Oxocyclohexanecarboxylate	[M-H]-	C7H10O3	141.05572			6.1E+04	1.3								
**75**	Octenoic acid	[M-H]-	C8H14O2	141.09210	4.1E+04	3.0										
**76**	Pyridine-3-aldehyde	[M+Cl]-	C6H5NO	142.00618											7.0E+04	2.4
**77**	Quinone	[M+Cl]-	C6H4O2	142.98986							3.2E+04	4.7				
**78**	n-valeryl acetic acid	[M-H]-	C7H12O3	143.07108							2.5E+04	2.0				
**79**	Dehydroxycarnitine	[M-H]-	C7H15NO2	144.10336							2.8E+04	-2.5				

#	Putative Annotation [a]	Ion	Formula	m/z Theo. [b]	R2020 [c]	ppm	W2020 [c]	ppm	B2020 [c]	ppm	R2021 [c]	ppm	W2021 [c]	ppm	B2021 [c]	ppm
**80**	Resorcinol	[M+Cl]-	C6H6O2	145.00701											3.8E+04	-2.5
**81**	Oxoglutarate	[M-H]-	C5H6O5	145.01425	9.3E+04	-4.7										
**82**	Coumarin	[M-H]-	C9H6O2	145.03000					1.7E+05	-3.4						
**83**	Glutamine	[M-H]-	C5H10N2O3	145.06193									4.4E+04	-0.4		
**84**	Cytosine	[M+Cl]-	C4H5N3O	146.01266	8.3E+04	-4.9			2.7E+04	-4.8						
**85**	Dihydroxyfumarate	[M-H]-	C4H4O6	146.99351			5.7E+04	-1.8								
**86**	Heptenal	[M+Cl]-	C7H12O	147.05904					2.4E+04	-4.6						
**87**	1-Pyrroline-2-carboxylate	[M+Cl]-	C5H7NO2	148.01644					1.9E+05	4.3						
**88**	threo-3-Hydroxy-L-aspartate	[M-H]-	C4H7NO5	148.02450							2.8E+04	4.4				
**89**	epsilon-Caprolactam	[M+Cl]-	C6H11NO	148.05336					4.8E+04	0.7						
**90**	Phenylglyoxylic acid	[M-H]-	C8H6O3	149.02427									4.3E+04	1.0	3.0E+04	2.4
**91**	Dipropyl disulfide	[M-H]-	C6H14S2	149.04642	6.2E+04	-4.9										
**92**	3-Amino-2-piperidone	[M+Cl]-	C5H10N2O	149.04815									1.4E+05	3.8		
**93**	Sulfinylpyruvic acid	[M-H]-	C3H3O5S	149.96284	3.4E+04	-0.9										
**94**	Sulfinylpyruvic acid	[M-H]-	C3H3O5S	149.96357					2.9E+04	-4.8						
**95**	6-Imino-5-oxocyclohexa-1,3-dienecarboxylate	[M-H]-	C7H5NO3	150.01967	4.4E+04	-2.8										
**96**	N3-Metyladenine	[M-H]-	C6H9N5	150.07852			7.4E+04	1.0	4.5E+04	1.1						
**97**	Fumaric acid	[M+Cl]-	C4H4O4	150.98052					4.7E+04	-1.1						
**98**	Sulfinoalanine	[M-H]-	C3H6NO4S	150.99448	4.0E+04	-4.0										
**99**	Arabitol	[M-H]-	C5H12O5	151.06120	7.1E+04	4.5										
**100**	5-Aminopentanamide	[M+Cl]-	C5H12N2O	151.06436			4.5E+04	1.0								
**101**	Myrtenol	[M-H]-	C10H16O	151.11381					3.5E+04	-4.0						
**102**	N-(4,6-diaminopyrimidin-5-yl)formamide	[M-H]-	C5H7N5O	152.05812					4.0E+04	-2.2						
**103**	Oxomalonate	[M+Cl]-	C3H2O5	152.95962			6.3E+04	0.7								
**104**	Imidazole pyruvic acid	[M-H]-	C6H6N2O3	153.03057	3.2E+04	-0.8			1.7E+04	-3.3						
**105**	Diaminobutyric acid	[M+Cl]-	C4H10N2O2	153.04462					3.1E+04	-4.6						
**106**	S-Acetylthioethanolamine	[M+Cl]-	C4H9NOS	154.00989			1.0E+05	1.3								
**107**	Nitrocatechol	[M-H]-	C6H5NO4	154.01458	3.7E+04	1.2										
**108**	Histidine	[M-H]-	C6H9N3O2	154.06220	8.0E+04	-4.9										
**109**	Dimethylsulfonioacetate	[M+Cl]-	C4H9O2S	156.00125									1.1E+05	3.1		
**110**	Selenocysteine seleninic acid	[M+Cl]-	C3H7NO4Se	156.00662					4.0E+04	1.9						
**111**	Phenylethylamine	[M+Cl]-	C8H11N	156.05885											2.8E+04	-1.9
**112**	Mercaptolactic acid	[M+Cl]-	C3H6O3S	156.97303							3.4E+04	0.9				
**113**	Nicotinamide	[M+Cl]-	C6H6N2O	157.01713					2.7E+04	1.8						
**114**	Allantoin	[M-H]-	C4H6N4O3	157.03721							3.4E+04	-3.2				
**115**	(+)-6-methyl caprylic acid	[M-H]-	C9H18O2	157.12340			4.2E+04	-2.2								
**116**	Guaiacol	[M+Cl]-	C7H8O2	159.02183	7.3E+04	1.0										
**117**	Pimelic acid	[M-H]-	C7H12O4	159.06628	8.4E+04	-0.3									4.2E+04	-4.4
**118**	D-Alanyl-D-alanine	[M-H]-	C6H12N2O3	159.07848									1.4E+05	-4.8		
**119**	Hydroxy-L-glutamic acid	[M-H]-	C5H7NO5	160.02542	3.1E+04	-1.7										

#	Putative Annotation [a]	Ion	Formula	$m/z_{Theo.}$ [b]	R2020 [c]	ppm	W2020 [c]	ppm	B2020 [c]	ppm	R2021 [c]	ppm	W2021 [c]	ppm	B2021 [c]	ppm
**120**	gamma-Coniceine	[M+Cl]-	C8H15N	160.08986							3.6E+04	-0.1				
**121**	Safrole	[M-H]-	C10H10O2	161.05984					7.0E+04	4.9						
**122**	Oleandrose	[M-H]-	C7H14O4	161.08232											2.9E+04	-2.4
**123**	3-Methyldioxyindole	[M-H]-	C9H9NO2	162.05663									1.7E+04	-3.6		
**124**	Octanal	[M+Cl]-	C8H16O	163.08873									2.7E+04	4.8		
**125**	Goitrin	[M+Cl]-	C5H7NOS	163.99351									2.1E+04	4.4		
**126**	Methionine S-oxide	[M-H]-	C5H11NO3S	164.03943							3.4E+04	-4.5				
**127**	D-Pipecolic acid	[M+Cl]-	C6H11NO2	164.04838			5.2E+04	-0.5								
**128**	Cucurbitine	[M+Cl]-	C5H10N2O2	165.04338									4.2E+04	1.5		
**129**	Agmatine	[M+Cl]-	C5H14N4	165.09131											9.8E+04	-0.4
**130**	4-Trimethylammoniobutanal	[M+Cl]-	C7H16NO	165.09323									3.5E+04	-3.9		
**131**	Iminoaspartate	[M+Cl]-	C4H5NO4	165.99126			7.2E+04	2.4								
**132**	Dihydroxyadenine	[M-H]-	C5H5N5O2	166.03668											3.0E+04	2.2
**133**	Guanidinopropanoate	[M+Cl]-	C4H9N3O2	166.03888	5.2E+04	-4.9			7.4E+04	5.0						
**134**	N-Carbamoylputrescine	[M+Cl]-	C5H13N3O	166.07554							2.2E+04	-1.7				
**135**	2-Dimethylamino-5,6-dimethylpyrimidin-4-ol	[M-H]-	C8H13N3O	166.09917											7.4E+04	-3.5
**136**	Oxaluric acid	[M+Cl]-	C3H4N2O4	166.98570									1.7E+04	4.8		
**137**	Glutaric acid	[M+Cl]-	C5H8O4	167.01156									1.7E+04	0.6		
**138**	Aspartic acid	[M+Cl]-	C4H7NO4	168.00723									1.5E+04	-1.9		
**139**	Piperidione	[M-H]-	C9H15NO2	168.10376											2.2E+04	-4.5
**140**	Dodecane	[M-H]-	C12H26	169.19617			3.1E+04	-1.0								
**141**	Decanamide	[M-H]-	C10H21NO	170.15471									1.8E+04	1.9		
**142**	Methylerythritol	[M+Cl]-	C5H12O4	171.04364									2.6E+04	-4.0		
**143**	Capric acid	[M-H]-	C10H20O2	171.13905	2.5E+05	1.6					1.0E+05	2.1				
**144**	Trigonelline	[M+Cl]-	C7H7NO2	172.01722											4.8E+04	-0.8
**145**	Quinaldic acid	[M-H]-	C10H7NO2	172.03969							2.0E+04	4.1				
**146**	N-Acetyl-L-leucine	[M-H]-	C8H15NO3	172.09799											2.3E+04	-0.4
**147**	2,4-nonadienal	[M+Cl]-	C9H14O	173.07387			3.8E+04	2.0								
**148**	Echinorine	[M-H]-	C11H12NO	173.08518							2.9E+04	-3.3				
**149**	Hydroxy pelargonic acid	[M-H]-	C9H18O3	173.11785									1.5E+04	2.7		
**150**	Histidinal	[M+Cl]-	C6H9N3O	174.04410											2.7E+04	-0.8
**151**	Acetyl phosphate	[M+Cl]-	C2H5O5P	174.95686	2.5E+04	-1.4										
**152**	Hydroxymethylcatechol	[M+Cl]-	C7H8O3	175.01759							1.8E+04	-4.8				
**153**	2-Oxo-6-methylthiohexanoic acid	[M-H]-	C7H12O3S	175.04286					2.8E+04	3.3						
**154**	Nonenal	[M+Cl]-	C9H16O	175.08909							2.0E+04	2.4				
**155**	O-Phosphoethanolamine	[M+Cl]-	C2H8NO4P	175.98846							2.9E+04	0.2				
**156**	Guvacoline	[M+Cl]-	C7H11NO2	176.04765					3.0E+04	4.1						
**157**	Valerianine	[M-H]-	C11H15NO	176.10747					2.0E+04	3.5						
**158**	Thiopheneacetic acid	[M+Cl]-	C6H6O2S	176.97885											4.9E+04	-3.4
**159**	Cysteinylglycine	[M-H]-	C5H10N2O3S	177.03328							2.1E+04	3.7				

#	Putative Annotation [a]	Ion	Formula	$m/z_{Theo.}$ [b]	R2020[c]	ppm	W2020[c]	ppm	B2020[c]	ppm	R2021[c]	ppm	W2021[c]	ppm	B2021[c]	ppm
**160**	Vinylacetylglycine	[M+Cl]-	C6H9NO3	178.02764	3.2E+04	0.5										
**161**	Glucose	[M-H]-	C6H12O6	179.05599									3.4E+05	0.7		
**162**	Dicyclohexylamine	[M-H]-	C12H23N	180.17521					1.8E+04	3.1						
**163**	Dodecenal	[M-H]-	C12H22O	181.15966											1.9E+04	0.7
**164**	Glutamic acid	[M+Cl]-	C5H9NO4	182.02344							2.3E+04	-4.8				
**165**	Thiouric acid	[M-H]-	C5H4N4O2S	182.99888											2.7E+04	-3.6
**166**	Chamazulene	[M-H]-	C14H16	183.11784									5.5E+04	0.5		
**167**	L-Serine O-sulfate	[M-H]-	C3H7NO6S	183.99213	3.2E+04	-2.2										
**168**	2-Formylaminobenzaldehyde	[M+Cl]-	C8H7NO2	184.01708			2.7E+05	-0.2								
**169**	D-Methionine	[M+Cl]-	C5H11NO2S	184.02045			2.7E+05	0.1								
**170**	Methyladenine	[M+Cl]-	C6H7N5	184.04000									1.4E+04	-2.5		
**171**	cis-2-Carboxycyclohexyl-acetic acid	[M-H]-	C9H14O4	185.08273							2.7E+04	-4.3				
**172**	2-Phenylglycine	[M+Cl]-	C8H9NO2	186.03273			4.3E+04	-0.6								
**173**	2-(Acetamidomethylene)succinate	[M-H]-	C7H9NO5	186.04143									2.1E+04	-3.4		
**174**	p-Cresol sulfate	[M-H]-	C7H8O4S	187.00632							2.3E+04	3.9				
**175**	Ngamma-Monomethyl-L-arginine	[M-H]-	C7H16N4O2	187.12005			5.3E+04	-1.0								
**176**	Thiocysteine	[M+Cl]-	C3H7NO2S2	187.96062							1.5E+04	3.2				
**177**	N-Amidino-L-glutamate	[M-H]-	C6H11N3O4	188.06768	2.8E+04	-3.0										
**178**	Sulfocatechol	[M-H]-	C6H6O5S	188.98635									2.2E+04	-0.2		
**179**	Ethoxycoumarin	[M-H]-	C11H10O3	189.05531							2.0E+04	2.2				
**180**	2-Oxo-7-methylthioheptanoic acid	[M-H]-	C8H14O3S	189.05909	3.1E+04	-0.5										
**181**	N-Methylpelletierine	[M+Cl]-	C9H17NO	190.10042			4.0E+04	-1.3							2.2E+05	4.3
**182**	Orotic acid	[M+Cl]-	C5H4N2O4	190.98568					4.2E+04	4.3						
**183**	Methylmuconolactone	[M+Cl]-	C7H8O4	191.01166	2.7E+04	0.6										
**184**	Quinic acid	[M-H]-	C7H12O6	191.05611	3.0E+04	3.3										
**185**	(-)-Citronellol	[M+Cl]-	C10H20O	191.12082			5.5E+04	-1.3								
**186**	Maleylacetate	[M+Cl]-	C6H6O5	192.99131									1.4E+04	-2.0		
**187**	Methyl beta-D-galactoside	[M-H]-	C7H14O6	193.07088									2.6E+04	4.6		
**188**	Glucosaminic acid	[M-H]-	C6H13NO6	194.06614									1.4E+04	4.5		
**189**	Aminopropylcadaverine	[M+Cl]-	C8H21N3	194.14363							2.3E+04	-3.5				
**190**	Dihydroxyphenylpyruvate	[M-H]-	C9H8O5	195.02968											1.8E+04	1.1
**191**	Benzocoumarin	[M-H]-	C13H8O2	195.04444									2.2E+04	3.7		
**192**	Guanidinoethyl methyl phosphate	[M-H]-	C4H12N3O4P	196.04975					2.3E+04	-2.5						
**193**	Hydroxymethyl-glutaric acid	[M+Cl]-	C6H10O5	197.02180							1.3E+04	2.2				
**194**	Hydroxylysine	[M+Cl]-	C6H14N2O3	197.06961							1.7E+04	1.2				
**195**	Threonine phosphate	[M-H]-	C4H10NO6P	198.01730	2.4E+04	-2.3										
**196**	3-Methyldioxyindole	[M+Cl]-	C9H9NO2	198.03273			3.7E+04	1.0								
**197**	4-(3-Pyridyl)-3-butenoic acid	[M+Cl]-	C9H9NO2	198.03283											1.3E+04	-0.5
**198**	Homomethionine	[M+Cl]-	C6H13NO2S	198.03610			3.7E+04	-2.0								
**199**	N-Acetylphenylethylamine	[M+Cl]-	C10H13NO	198.06884											2.1E+04	1.4

#	Putative Annotation [a]	Ion	Formula	$m/z_{Theo.}$ [b]	R2020[c]	ppm	W2020[c]	ppm	B2020[c]	ppm	R2021[c]	ppm	W2021[c]	ppm	B2021[c]	ppm
**200**	Mephentermine	[M+Cl]-	C11H17N	198.10531											1.7E+04	1.0
**201**	Hydroxycinnamate	[M+Cl]-	C9H8O3	199.01723							2.4E+04	-2.4				
**202**	Phenylheptatriyne	[M+Cl]-	C13H8	199.03228											2.1E+04	-1.4
**203**	Propionyl caprylic acid	[M-H]-	C11H20O3	199.13467					2.6E+04	-3.5						
**204**	Lauric acid	[M-H]-	C12H24O2	199.17035	4.4E+05	2.4			2.6E+04	-0.7			2.0E+05	-2.4		
**205**	Hydroxy hendecanoic acid	[M-H]-	C11H22O3	201.14957									1.9E+04	0.2		
**206**	Butanoylphosphate	[M+Cl]-	C4H9O5P	202.98900					2.2E+04	-4.1						
**207**	Pyridoxamine	[M+Cl]-	C8H12N2O2	203.05928	2.4E+04	4.8										
**208**	N6-Acetyl-N6-hydroxy-L-lysine	[M-H]-	C8H16N2O4	203.10286					3.0E+04	4.3						
**209**	Bellendine	[M-H]-	C12H15NO2	204.10387							1.6E+04	-4.3				
**210**	Methoxycinnamic acid ethyl ester	[M-H]-	C12H14O3	205.08702	1.9E+04	4.5										
**211**	Actinamine	[M-H]-	C8H18N2O4	205.11897									2.7E+04	2.0		
**212**	Octylphenol	[M-H]-	C14H22O	205.15979	5.6E+04	-4.5										
**213**	4-(1,2-Epoxyethyl)-8,9-epoxy-enediyne	[M-H]-	C14H8O2	207.04515			1.0E+05	-2.9								
**214**	Dihydrolipoic acid	[M-H]-	C8H16O2S2	207.05171									8.3E+04	0.9		
**215**	Glycylproline	[M+Cl]-	C7H12N2O3	207.05462							2.9E+04	-2.1				
**216**	Undecan-1-ol	[M+Cl]-	C11H24O	207.15174					4.0E+04	1.8						
**217**	5-Deoxy-5-aminoshikimic acid	[M+Cl]-	C7H11NO4	208.03821	1.8E+04	-2.4									1.4E+04	-4.4
**218**	1,3-Dimethyl-8-isoquinolinol	[M+Cl]-	C11H11NO	208.05284							2.4E+04	3.0				
**219**	Benzimidazolylguanidine	[M+Cl]-	C8H9N5	210.05515					2.9E+04	0.2						
**220**	Perseitol	[M-H]-	C7H16O7	211.08145									1.4E+04	4.2		
**221**	Myristaldehyde	[M-H]-	C14H28O	211.20607							1.2E+04	3.2				
**222**	2-Hydroxy-6-oxonona-2,4-diene-1,9-dioate	[M-H]-	C9H10O6	213.03988							2.4E+04	2.7				
**223**	Methyl lauric acid	[M-H]-	C13H26O2	213.18619							1.7E+05	-0.9				
**224**	5-Methyl-3-isoxazolyl sulfate	[M+Cl]-	C4H5NO5S	213.95786					4.4E+04	1.8						
**225**	Kinetin	[M-H]-	C10H9N5O	214.07419											3.0E+04	-3.5
**226**	3-Phospho-D-erythronate	[M-H]-	C4H9O8P	214.99623			2.1E+05	-1.6								
**227**	Bergapten	[M-H]-	C12H8O4	215.03587							5.7E+04	-4.1	3.1E+05	0.6		
**228**	2-Methyl-3-hydroxy-5-formylpyridine-4-carboxylate	[M+Cl]-	C8H7NO4	216.00666											7.8E+04	1.2
**229**	2-(Hydroxymethyl)-3-(acetamidomethylene)succinate	[M-H]-	C8H11NO6	216.05057					2.0E+04	3.7						
**230**	O-Propanoylcarnitine	[M-H]-	C10H19NO4	216.12413			3.8E+04	-1.6								
**231**	cis-1,2-Dihydroxy-1,2-dihydrodibenzothiophene	[M-H]-	C12H10O2S	217.03205							3.0E+04	3.8				
**232**	Diisopropyl phosphate	[M+Cl]-	C6H15O4P	217.04020	3.3E+04	4.0										
**233**	Xanthan	[M+Cl]-	C13H10O	217.04257			1.1E+05	2.3								
**234**	5-Methoxydimethyltryptamine	[M-H]-	C13H18N2O	217.13464			4.1E+04	0.3								
**235**	L-Homocysteic acid	[M+Cl]-	C4H9NO5S	217.98967											1.4E+04	-0.6
**236**	4-Amino-5-phosphomethyl-2-methylpyrimidine	[M-H]-	C6H10N3O4P	218.03385									1.2E+04	-1.1		
**237**	Ngamma-Nitro-L-arginine	[M-H]-	C6H13N5O4	218.08906					1.8E+04	1.9						

#	Putative Annotation [a]	Ion	Formula	m/z $_{Theo.}$ [b]	R2020 [c]	ppm	W2020 [c]	ppm	B2020 [c]	ppm	R2021 [c]	ppm	W2021 [c]	ppm	B2021 [c]	ppm
**238**	N-Phenyl-1-naphthylamine	[M-H]-	C16H13N	218.09776									1.5E+04	-1.1		
**239**	Tecostanine	[M+Cl]-	C11H21NO	218.13207					1.7E+04	-1.6					1.4E+04	-3.5
**240**	Polygonolide	[M-H]-	C12H12O4	219.06628	1.8E+04	2.8										
**241**	Chamazulene	[M+Cl]-	C14H16	219.09499									1.6E+04	-1.8	2.5E+04	0.0
**242**	Dihydroxykynurenate	[M-H]-	C10H7NO5	220.02507							1.3E+04	0.3				
**243**	Sulfomuconate	[M-H]-	C6H6O7S	220.97563									1.6E+04	2.3		
**244**	Undecanoic acid	[M+Cl]-	C11H22O2	221.13076					2.0E+04	2.8						
**245**	n-Decanohydroxamic acid	[M+Cl]-	C10H21NO2	222.12604					2.0E+04	2.7						
**246**	Methylaconitate	[M+Cl]-	C7H8O6	223.00149	2.3E+04	-2.7										
**247**	Dihydroflavone	[M-H]-	C15H12O2	223.07702									1.4E+04	-2.5		
**248**	Prenyl-L-cysteine	[M+Cl]-	C8H15NO2S	224.05259							1.3E+04	-3.7	1.3E+04	-4.4		
**249**	Homocitrulline	[M+Cl]-	C7H15N3O3	224.08074			3.3E+04	-2.9								
**250**	Methylthiooctanaldoxime	[M+Cl]-	C9H19NOS	224.08801							1.3E+04	0.6				
**251**	Myristoleic acid	[M-H]-	C14H26O2	225.18600	3.2E+04	1.7										
**252**	Myristic acid	[M-H]-	C14H28O2	227.20176									1.3E+06	-0.5		
**253**	N-Decanoylglycine	[M-H]-	C12H23NO3	228.16100									3.4E+05	-2.1		
**254**	Ferulic acid	[M+Cl]-	C10H10O4	229.02731			1.9E+05	2.1								
**255**	Zingerone	[M+Cl]-	C11H14O3	229.06384									2.7E+04	-0.6		
**256**	Hydroxypseudooxynicotine	[M+Cl]-	C10H14N2O2	229.07493	2.9E+04	3.8			5.9E+04	4.5						
**257**	Xanthine-8-carboxylate	[M+Cl]-	C6H4N4O4	230.99266	2.4E+04	-1.8										
**258**	Gluconic acid	[M+Cl]-	C6H12O7	231.02840											2.8E+04	-3.0
**259**	Hydroxyatrazine	[M+Cl]-	C8H15N5O	232.09700							2.5E+04	0.3				
**260**	Curcumenol	[M-H]-	C15H22O2	233.15472											1.9E+04	-0.1
**261**	Benzoxazolinate	[M-H]-	C11H9NO5	234.04018					1.8E+04	2.6						
**262**	D-Erythrose 4-phosphate	[M+Cl]-	C4H9O7P	234.97857											1.3E+04	-2.5
**263**	Indolepyruvic acid	[M+Cl]-	C11H9NO3	238.02764	2.0E+04	3.9										
**264**	Dehydrofalcarinone	[M-H]-	C17H20O	239.14451							4.2E+04	-1.6	4.3E+04	1.4		
**265**	Tridecenyl acetate	[M-H]-	C15H28O2	239.20131							1.3E+05	1.4				
**266**	N-(2,3-Dihydroxybenzoyl)-L-serine	[M-H]-	C10H11NO6	240.05131							2.1E+04	0.2				
**267**	4-(Dimethylamino)phenylazoxybenzene	[M-H]-	C14H15N3O	240.11424	1.8E+04	1.0										
**268**	Methyl myristic acid	[M-H]-	C15H30O2	241.21730	3.4E+05	1.0	1.6E+06	0.5					3.0E+05	-2.4		
**269**	Methyl 5-hydroxy-2-benzimidazole carbamate	[M+Cl]-	C9H9N3O3	242.03363											2.1E+04	0.7
**270**	N-Undecanoylglycine	[M-H]-	C13H25NO3	242.17628							1.8E+05	-0.5			8.4E+04	-2.2
**271**	Jasmonic acid	[M+Cl]-	C12H18O3	245.09446											1.3E+04	2.2
**272**	3-(2-Carboxyethenyl)-cis,cis-muconate	[M+Cl]-	C9H8O6	247.00120							8.2E+04	1.2				
**273**	Confertin	[M-H]-	C15H20O3	247.13400					1.6E+04	-0.1						
**274**	N-Caffeoylputrescine	[M-H]-	C13H18N2O3	249.12477					2.2E+04	-1.2						
**275**	Abscisic alcohol	[M-H]-	C15H22O3	249.15051									1.4E+04	-3.6		
**276**	Glycerylphosphorylethanolamine	[M+Cl]-	C5H14NO6P	250.02528	2.7E+04	2.8										
**277**	cis-Hinokiresinol	[M-H]-	C17H16O2	251.10854					3.0E+04	-3.1						

#	Putative Annotation [a]	Ion	Formula	m/z $_{Theo.}$ [b]	R2020[c]	ppm	W2020[c]	ppm	B2020[c]	ppm	R2021[c]	ppm	W2021[c]	ppm	B2021[c]	ppm
**278**	Vulgraon B	[M+Cl]-	C16H24	251.15795							7.1E+04	-3.0				
**279**	S-(Phenylacetothiohydroximoyl)-L-cysteine	[M-H]-	C11H14N2O3S	253.06524	3.2E+04	-2.3										
**280**	Palmitoleic acid	[M-H]-	C16H30O2	253.21719									3.6E+05	0.5		
**281**	Nicotinate D-ribonucleoside	[M-H]-	C11H14NO6	255.07484	2.0E+05	-0.6										
**282**	N-Acetyl-beta-D-glucosaminylamine	[M+Cl]-	C8H16N2O5	255.07532	2.0E+05	-2.5										
**283**	Palmitic acid	[M-H]-	C16H32O2	255.23295	1.3E+06	-0.4	4.5E+06	-0.2	5.3E+05	-1.1	7.4E+05	2.8			3.2E+05	1.0
**284**	Benzythioguanine	[M-H]-	C12H11N5S	256.06568					2.6E+04	2.2						
**285**	Hydroxykynurenine	[M+Cl]-	C10H12N2O4	259.04857											1.9E+04	2.1
**286**	Hemigossypol	[M-H]-	C15H16O4	259.09758			3.2E+04	1.1								
**287**	1-Hydroxy-2-methyl-2-butenyl 4-diphosphate	[M-H]-	C5H12O8P2	260.99314					1.6E+04	1.2						
**288**	Chorismic acid	[M+Cl]-	C10H10O6	261.01660											7.5E+04	2.1
**289**	N-acetyl-S-(3-oxo-3-carboxy-n-propyl)cysteine	[M-H]-	C9H13NO6S	262.03969									2.8E+04	-2.3		
**290**	Serine phosphoethanolamine	[M+Cl]-	C5H13N2O6P	263.02052					2.1E+04	0.0						
**291**	Myristic acid	[M+Cl]-	C14H28O2	263.17833	2.7E+04	2.1	2.6E+06	0.4			1.4E+06	-2.1	1.3E+04	1.9		
**292**	Xanthoxic acid	[M-H]-	C15H22O4	265.14385											7.4E+04	2.6
**293**	Pentadecadienyl acetate	[M-H]-	C17H30O2	265.21730	6.7E+04	3.7										
**294**	N-Succinyl-L-glutamate 5-semialdehyde	[M+Cl]-	C9H13NO6	266.04416							1.6E+04	-1.8				
**295**	Hexadecatrienal	[M+Cl]-	C16H26O	269.16777	6.1E+04	-2.8			2.1E+04	4.2						
**296**	2,4'-Dihydroxy-4-methoxydihydrochalcone	[M-H]-	C16H16O4	271.09697									3.1E+04	2.3		
**297**	Glutaconylcarnitine	[M-H]-	C12H19NO6	272.11442							2.2E+04	-1.7				
**298**	Methoxychalcone	[M+Cl]-	C16H14O2	273.06888									3.3E+04	-0.4		
**299**	Ranunculin	[M-H]-	C11H16O8	275.07640					4.5E+04	3.1						
**300**	Cyperaquinone	[M+Cl]-	C14H10O4	277.02652									3.4E+04	2.9		
**301**	Thymidine	[M+Cl]-	C10H14N2O5	277.06028					2.3E+04	-2.2						
**302**	Pantetheine	[M-H]-	C11H22N2O4S	277.12275	2.3E+04	2.4										
**303**	Linoleic acid	[M-H]-	C18H32O2	279.23357											4.7E+04	-2.2
**304**	7-N,N-Dimethylamino-1,2,3,4,5-pentathiocyclooctane	[M+Cl]-	C5H11NS5	279.91979											1.5E+04	-3.2
**305**	Oleamide	[M-H]-	C18H35NO	280.26471											2.1E+04	-0.4
**306**	Oleic acid	[M-H]-	C18H34O2	281.24860	1.8E+05	1.1			1.9E+05	1.2	1.3E+05	-0.3	2.0E+05	-0.1	6.0E+04	-2.0
**307**	Stearic acid	[M-H]-	C18H36O2	283.26425	7.9E+05	-1.2	1.6E+06	-0.4	1.9E+05	-0.5	3.4E+05	0.9				
**308**	C17 Sphingosine	[M-H]-	C17H35NO2	284.25933					3.7E+04	0.6						
**309**	4'-O-Methylisoflavone	[M+Cl]-	C16H12O3	287.04708											4.6E+04	3.4
**310**	Dihydroxy palmitic acid	[M-H]-	C16H32O4	287.22210					2.3E+05	2.4	2.3E+05	1.1				
**311**	All-trans-13,14-dihydroretinol	[M-H]-	C20H32O	287.23804			1.1E+05	1.2								
**312**	5-(3'-Carboxy-3'-oxopropenyl)-4,6-dihydroxypicolinate	[M+Cl]-	C10H7NO7	287.99126									2.1E+04	1.4		
**313**	methyl 4-[2-(2-formyl-vinyl)-3-hydroxy-5-oxo-cyclopentyl]-butanoate	[M+Cl]-	C13H18O5	289.08580					2.3E+05	-3.4						
**314**	N-(4-Aminobutyryl)-L-histidine	[M+Cl]-	C11H18N4O3	289.10760											1.2E+05	-1.1

#	Putative Annotation [a]	Ion	Formula	m/z Theo. [b]	R2020 [c]	ppm	W2020 [c]	ppm	B2020 [c]	ppm	R2021 [c]	ppm	W2021 [c]	ppm	B2021 [c]	ppm
**315**	5-O-Methylvisamminol	[M-H]-	C16H18O5	289.10760											1.2E+05	1.9
**316**	2-Deoxy-2,3-dehydro-N-acetylneuraminic acid	[M-H]-	C11H17NO8	290.08789									2.0E+04	0.9		
**317**	Phenylbutyrylglutamine	[M-H]-	C15H20N2O4	291.13500											2.0E+04	0.1
**318**	Tutin	[M-H]-	C15H18O6	293.10396							7.0E+04	-3.1				
**319**	Gingerol	[M-H]-	C17H26O4	293.17675											3.3E+04	-3.1
**320**	Khellin	[M+Cl]-	C14H12O5	295.03788	2.9E+04	0.5										
**321**	L-gamma-glutamyl-L-isoleucine	[M+Cl]-	C11H20N2O5	295.10724											1.7E+04	-2.1
**322**	Methylaminoadenosine	[M-H]-	C11H16N6O4	295.11639									1.8E+04	-1.2		
**323**	Beta-Acetoxy-4-oplopanone	[M-H]-	C17H28O4	295.19148	5.2E+04	-3.1										
**324**	Phosphotyrosine	[M+Cl]-	C9H12NO6P	296.00963	3.0E+04	-2.5										
**325**	Longifolonine	[M-H]-	C17H15NO4	296.09379									2.1E+04	-3.2		
**326**	3-Methoxy-4-Hydroxyphenylglycol sulfate	[M+Cl]-	C9H12O7S	299.00001											1.8E+04	-0.8
**327**	Kaempferol 4'-methyl ether	[M-H]-	C16H12O6	299.05611	3.8E+04	1.3										
**328**	MG(0:0/14:1(9Z)/0:0)	[M-H]-	C17H32O4	299.22214							1.8E+05	2.2				
**329**	Methoxybrassinin	[M+Cl]-	C12H14N2OS2	301.02389									2.2E+04	0.9		
**330**	MG(0:0/14:0/0:0)	[M-H]-	C17H34O4	301.23867									1.2E+05	-0.8		
**331**	2-Amino-4-oxo-6-(1',2'-dioxoprolyl)-7,8-dihydroxypteridine	[M+Cl]-	C9H9N5O5	302.03041									1.7E+04	-2.1		
**332**	Coumestrol	[M+Cl]-	C15H8O5	303.00657	8.6E+04	-1.1										
**333**	3',4'-Methylenedioxyfurano[2'',3'':6,7]aurone	[M-H]-	C18H10O5	305.04555			1.7E+05	0.1								
**334**	Gallocatechin	[M-H]-	C15H14O7	305.06669					2.7E+04	0.0						
**335**	Isolonchocarpin	[M-H]-	C20H18O3	305.11798							4.7E+04	1.1				
**336**	Eicosatrienoic acid	[M-H]-	C20H34O2	305.24929					3.7E+04	-2.2						
**337**	Octadecatriynoic acid	[M+Cl]-	C18H24O2	307.14613							2.1E+04	2.9				
**338**	Cinmethylin	[M+Cl]-	C18H26O2	309.16357											3.2E+04	-2.9
**339**	Botrydial	[M-H]-	C17H26O5	309.17075	1.8E+05	-1.4										
**340**	Sauroxine	[M+Cl]-	C17H26N2O	309.17392					4.2E+05	0.0						
**341**	PC(0:0/3:1(2E))	[M-H]-	C11H22NO7P	310.10524					3.5E+04	2.8						
**342**	Epsilon-(gamma-Glutamyl)-lysine	[M+Cl]-	C11H21N3O5	310.11752	1.0E+05	-0.2										
**343**	Jatrophone	[M-H]-	C20H24O3	311.16444											4.7E+04	2.7
**344**	10,12-octadecadiynoic acid	[M+Cl]-	C18H28O2	311.17833			9.5E+05	1.7								
**345**	Phosphoribosylformylglycineamidine	[M-H]-	C8H16N3O8P	312.06018											4.4E+04	0.1
**346**	4-Oxo-13-cis-retinoate	[M-H]-	C20H25O3	312.17309			1.6E+05	0.8								
**347**	Octadecanedioic acid	[M-H]-	C18H34O4	313.23799					8.2E+04	1.4						
**348**	MG(15:0/0:0/0:0)	[M-H]-	C18H36O4	315.25477							5.2E+04	-2.2				
**349**	Caffeic acid phenethyl ester	[M+Cl]-	C17H16O4	319.07426	4.9E+04	0.8										
**350**	gamma-L-Glutamyl-L-cysteinyl-beta-alanine	[M-H]-	C11H19N3O6S	320.09218	3.5E+04	2.1										
**351**	Cytidine-5'-monophosphate	[M-H]-	C9H14N3O8P	322.04457	4.4E+04	2.3										
**352**	Fructose 1,6-bisphosphate	[M-H]-	C6H14O11P2	322.99386	9.7E+04	0.3										
**353**	Portulacaxanthin I	[M-H]-	C14H16N2O7	323.08920											1.5E+04	-2.2

#	Putative Annotation [a]	Ion	Formula	m/z Theo. [b]	R2020 [c]	ppm	W2020 [c]	ppm	B2020 [c]	ppm	R2021 [c]	ppm	W2021 [c]	ppm	B2021 [c]	ppm
**354**	Karwinaphthol B	[M+Cl]-	C17H20O4	323.10463							1.6E+04	2.9				
**355**	Isoacitretin	[M-H]-	C21H26O3	325.18178					2.9E+05	-2.7						
**356**	Colnelenic acid	[M+Cl]-	C18H28O3	327.17325	5.8E+05	0.0										
**357**	3,7,11,15-Tetramethyl-6,10,14-hexadecatrien-1-ol	[M+Cl]-	C20H36O	327.24670									3.2E+04	-2.1		
**358**	MG(0:0/16:0/0:0)	[M-H]-	C19H38O4	329.27043												
**359**	Gibberellin A12	[M-H]-	C20H28O4	331.19140							7.0E+04	0.3				
**360**	Longifolonine	[M+Cl]-	C17H15NO4	332.06971									2.6E+04	-0.6		
**361**	Heptahydroxyflavone	[M-H]-	C15H10O9	333.02521	5.2E+04	-2.7										
**362**	Kaempferol 4'-methyl ether	[M+Cl]-	C16H12O6	335.03279			7.9E+04	-0.4								
**363**	Umbellatine	[M-H]-	C20H18NO4	335.11574											3.0E+04	1.7
**364**	Maximaisoflavone J	[M-H]-	C21H20O4	335.12986									2.3E+04	-2.9		
**365**	6-hydroperoxy-4E,8Z,11Z,14Z-eicosatetraenoic acid	[M-H]-	C20H32O4	335.22247											2.3E+04	0.9
**366**	N-Acetyl-D-glucosamine 6-phosphate	[M+Cl]-	C8H16NO9P	336.02567	6.2E+04	-1.4										
**367**	S-(Hydroxymethyl)glutathione	[M-H]-	C11H19N3O7S	336.08700									3.5E+04	0.3		
**368**	(Ac)2-L-lysyl-D-alanine	[M+Cl]-	C13H23N3O5	336.13295					2.7E+04	0.7						
**369**	Neotenone	[M-H]-	C19H14O6	337.07117									2.9E+04	1.8		
**370**	5,7-Dimethoxy-8-prenylflavan	[M-H]-	C22H26O3	337.18092	1.8E+05	2.3										
**371**	Eicosapentaenoic acid	[M+Cl]-	C20H30O2	337.19389							4.0E+04	0.3				
**372**	1beta,3beta,5alpha,6beta-tetrahydroxyandrostan-17-one	[M-H]-	C19H30O5	337.20205			3.9E+05	-1.1								
**373**	Disopyramide	[M-H]-	C21H29N3O	338.22294											2.2E+04	2.5
**374**	Oleoyl glycine	[M-H]-	C20H37NO3	338.27007	1.4E+05	1.6										
**375**	Aristolochic acid	[M-H]-	C17H11NO7	340.04591					3.2E+04	1.1						
**376**	Lactosamine	[M-H]-	C12H23NO10	340.12500									1.7E+04	-0.2		
**377**	Lupinate	[M+Cl]-	C13H18N6O3	341.11344			2.1E+05	2.1								
**378**	Matricin	[M+Cl]-	C17H22O5	341.11706											5.4E+04	-2.7
**379**	11,14-eicosadienoic acid	[M+Cl]-	C20H36O2	343.24093			1.3E+05	1.2								
**380**	Ovalifolin	[M-H]-	C22H18O4	345.11386									1.6E+04	-1.8		
**381**	O-Arachidonoyl Ethanolamine	[M-H]-	C22H37NO2	346.27515			3.7E+04	-0.9								
**382**	Triphenyl-1-hexene	[M+Cl]-	C24H24	347.15785					2.6E+04	-1.9						
**383**	Hydroxybowdichione	[M+Cl]-	C16H10O7	349.01284									2.2E+04	-2.3		
**384**	N-Acetylthienamycin	[M+Cl]-	C13H18N2O5S	349.06262											2.2E+04	1.2
**385**	Vincamine	[M-H]-	C21H26N2O3	353.18699									5.8E+04	0.2		
**386**	5-hydroperoxy-7-[3,5-epidioxy-2-(2-octenyl)-cyclopentyl]-6-heptenoic acid	[M-H]-	C19H30O6	353.19603					2.8E+05	2.6	1.7E+05	-2.1				
**387**	Spirasine I	[M-H]-	C22H29NO3	354.20707					6.9E+04	1.1						
**388**	5-O-(1-Carboxyvinyl)-3-phosphoshikimate	[M+Cl]-	C10H13O10P	358.99336											2.1E+04	1.9
**389**	Dityrosine	[M-H]-	C18H20N2O6	359.12403							2.6E+04	2.3				
**390**	p-Coumaroyl-D-glucose	[M+Cl]-	C15H18O8	361.07042											1.7E+04	-2.4

#	Putative Annotation [a]	Ion	Formula	$m/z_{Theo.}$ [b]	R2020 [c]	ppm	W2020 [c]	ppm	B2020 [c]	ppm	R2021 [c]	ppm	W2021 [c]	ppm	B2021 [c]	ppm
**391**	Shikodonin	[M-H]-	C20H26O6	361.16566			3.8E+04	2.5								
**392**	Europine	[M+Cl]-	C16H27NO6	364.15385					1.4E+05	-1.7						
**393**	Palmitoyl Serinol	[M+Cl]-	C19H39NO3	364.26289							3.7E+04	-1.4				
**394**	7'-carboxy-gama-tocotrienol	[M+Cl]-	C20H27O4	366.16107									1.9E+04	-2.0		
**395**	Catechin 5,7,3'-trimethyl ether	[M+Cl]-	C18H20O6	367.09570									3.6E+04	-0.8		
**396**	Hydroxy-PGE1	[M-H]-	C20H34O6	369.22818							2.6E+04	0.2				
**397**	Docosanedioic acid	[M-H]-	C22H42O4	369.30103			7.0E+04	2.4								
**398**	10,11-dihydro-12-oxo-LTB4	[M+Cl]-	C20H31O4	370.19125									1.9E+04	1.0		
**399**	3-Epihydroxymugineic acid	[M+Cl]-	C12H20N2O9	371.08548									1.5E+04	2.2		
**400**	1-Acetylaspidoalbidine	[M+Cl]-	C21H26N2O2	373.16869											2.2E+04	0.4
**401**	9'-Carboxy-gama-chromanol	[M-H]-	C23H35O4	374.24626			3.8E+04	-2.6								
**402**	Riboflavin	[M-H]-	C17H20N4O6	375.13087					3.4E+04	0.4						
**403**	PGE1 alcohol	[M+Cl]-	C20H36O4	375.23076	2.9E+04	1.3										
**404**	Brosimacutin C	[M+Cl]-	C20H22O5	377.11641									2.0E+04	-0.8		
**405**	Eicosanedioic acid	[M+Cl]-	C20H38O4	377.24641			2.8E+05	-1.5								
**406**	N-(7-Mercaptoheptanoyl)threonine 3-O-phosphate	[M+Cl]-	C11H22NO7PS	378.05459					3.5E+04	0.7						
**407**	Gibberellic acid	[M+Cl]-	C19H22O6	381.11053									3.3E+04	1.3		
**408**	5-(Heptadec-12-enyl)resorcinol	[M+Cl]-	C23H38O2	381.25658	9.8E+04	1.6										
**409**	Trihydroxy-7,3',4'-trimethoxyisoflavan	[M+Cl]-	C18H20O7	383.08988							1.5E+04	1.1				
**410**	24-hydroxy-tetracosanoic acid	[M-H]-	C24H48O3	383.35307			4.9E+04	0.3								
**411**	S-(Formylmethyl)glutathione	[M+Cl]-	C12H19N3O7S	384.06340							2.4E+04	1.0				
**412**	O-Feruloylgalactarate	[M-H]-	C16H18O11	385.07727											2.0E+04	0.9
**413**	5,3',4',5'-Tetramethoxy-6,7-methylenedioxyflavone	[M-H]-	C20H18O8	385.09289			3.1E+04	-2.1								
**414**	Mercaptoacetyl-Phe-Leu	[M+Cl]-	C17H24N2O4S	387.11573									5.1E+04	-1.7		
**415**	Palmatine	[M+Cl]-	C21H22NO4	387.12471					3.9E+04	-1.1						
**416**	5-Hydroxy-6-methoxy-3',4'-methylenedioxyfurano[2'',3'':7,8]flavanone	[M+Cl]-	C19H14O7	389.04335			6.0E+04	-2.4								
**417**	Latifoline	[M-H]-	C20H27NO7	392.17086									2.7E+04	1.6		
**418**	2,2-Dimethyl-3-(4-methoxyphenyl)-4-ethyl-6-(1-pyrrolidinylmethyl)-2H-1-benzopyran-7-ol	[M-H]-	C25H31NO3	392.22312			5.4E+04	0.2								
**419**	PA(15:0/0:0)	[M-H]-	C18H37O7P	395.22057					6.6E+04	-0.4						
**420**	N-Arachidonoyl glycine	[M+Cl]-	C22H35NO3	396.23016					2.6E+04	2.4						
**421**	Hexadecenoylcarnitine	[M-H]-	C23H43NO4	396.31193	2.6E+04	1.5										
**422**	Gibberellin A19	[M+Cl]-	C20H26O6	397.14172									2.1E+04	1.6		
**423**	Erythronolide B	[M-H]-	C21H38O7	401.25403									2.1E+04	1.1		
**424**	Glucoiberverin	[M-H]-	C11H21NO9S3	406.02982											2.3E+04	1.8
**425**	Ginkgolide A	[M-H]-	C20H24O9	407.13396											1.9E+04	2.0
**426**	Cholic acid	[M-H]-	C24H40O5	407.27960									3.0E+04	1.7		

#	Putative Annotation [a]	Ion	Formula	m/z Theo. [b]	R2020[c]	ppm	W2020[c]	ppm	B2020[c]	ppm	R2021[c]	ppm	W2021[c]	ppm	B2021[c]	ppm
427	4Z,7Z,10Z,13Z,16Z,19Z,22Z,25Z-octacosaoctaenoic acid	[M-H]-	C28H40O2	407.29555			1.6E+05	-2.4								
428	N-oleoyl glutamine	[M-H]-	C23H42N2O4	409.30718	4.8E+04	0.6										
429	Ovaliflavanone A	[M+Cl]-	C25H28O3	411.17391											1.3E+05	-1.6
430	Trimethyl-5Z,9Z,19Z-docosatrienoic acid	[M+Cl]-	C25H44O2	411.30383					4.3E+04	-0.7						
431	Cryptopleurine	[M+Cl]-	C24H27NO3	412.16779									7.8E+04	1.7		
432	22:2(5Z,9Z)(13Me,17Me,21Me)	[M+Cl]-	C25H46O2	413.31918			3.8E+04	2.8								
433	4-Deoxy-beta-D-gluc-4-enuronosyl-(1,3)-N-acetyl-D-galactosamine	[M+Cl]-	C14H21NO11	414.08086	7.0E+04	-1.6										
434	Briantheine W	[M-H]-	C24H32O6	415.21178					2.5E+04	2.0						
435	Dihydroxycholecalciferol	[M-H]-	C27H44O3	415.32177			5.2E+04	-2.1								
436	beta-Tocopherol	[M-H]-	C28H48O2	415.35786											1.9E+04	0.7
437	Syringaresinol	[M-H]-	C22H26O8	417.15579					2.3E+04	-0.7						
438	N-stearoyl valine	[M+Cl]-	C23H45NO3	418.30941					2.5E+04	-0.2						
439	Lamioside	[M-H]-	C18H28O11	419.15580									2.6E+04	0.2		
440	N-stearoyl histidine	[M-H]-	C24H43N3O3	420.32317									1.8E+04	0.0		
441	8-Cinnamoyl-3,4-dihydro-5,7-dihydroxy-4-phenylcoumarin	[M+Cl]-	C24H18O5	421.08506					2.2E+04	-0.6						
442	Militarinone C	[M-H]-	C26H33NO4	422.23299									5.5E+04	1.6		
443	Quassin	[M+Cl]-	C22H28O6	423.15799			3.8E+04	1.3								
444	Chitobiose	[M-H]-	C16H28N2O11	423.16203			3.8E+04	-1.0								
445	Didrovaltratum	[M-H]-	C22H32O8	423.20244	6.7E+04	-0.6										
446	1alpha,25-dihydroxy-26,27-dinorvitamin D3	[M+Cl]-	C25H40O3	423.26685											2.4E+04	0.7
447	Montanic acid	[M-H]-	C28H56O2	423.42075	4.3E+04	-1.9										
448	Jervine	[M-H]-	C27H39NO3	424.28496							3.0E+04	1.8				
449	Bispyribac acid	[M-H]-	C19H18N4O8	429.10432											2.5E+04	2.0
450	PA(P-16:0/0:0)	[M+Cl]-	C19H39O6P	429.21783			5.3E+04	-1.3								
451	Stanolone benzoate	[M+Cl]-	C26H34O3	429.22020			5.3E+04	0.3								
452	Heptacosanol	[M+Cl]-	C27H56O	431.40174							2.1E+04	1.8				
453	Echimidine	[M+Cl]-	C20H31NO7	432.17893							1.6E+04	1.2				
454	PC(O-12:0/O-1:0)	[M-H]-	C21H46NO6P	438.29833							2.8E+04	1.5				
455	Bleekerine	[M+Cl]-	C23H24N2O5	443.13780							2.1E+04	0.3				
456	(22E)-1alpha-hydroxy-24-oxo-26,27-cyclo-22,23-didehydrovitamin D3	[M+Cl]-	C27H38O3	445.25150			5.0E+04	-1.9								
457	Calophyllolide	[M+Cl]-	C26H24O5	451.13172					2.5E+04	0.1						
458	Calcitriol	[M+Cl]-	C27H44O3	451.29889											1.7E+04	-1.0
459	8-Caffeoyl-3,4-dihydro-5,7-dihydroxy-4-phenylcoumarin	[M+Cl]-	C24H18O7	453.07465			4.8E+04	-2.7								
460	Lactose 6-phosphate	[M+Cl]-	C12H23O14P	457.05194			1.2E+05	-1.8								
461	Eruberin A	[M-H]-	C25H30O8	457.18679	1.8E+04	-0.9										

#	Putative Annotation [a]	Ion	Formula	$m/z_{\text{Theo.}}$ [b]	R2020[c]	ppm	W2020[c]	ppm	B2020[c]	ppm	R2021[c]	ppm	W2021[c]	ppm	B2021[c]	ppm
**462**	CDP-N-methylethanolamine	[M-H]-	C12H22N4O11P2	459.06802											2.9E+04	1.6
**463**	Rutarin	[M+Cl]-	C20H24O10	459.10544					2.4E+04	2.0						
**464**	PA(20:3(8Z,11Z,14Z)/0:0)	[M-H]-	C23H41O7P	459.25171			4.8E+04	1.2								
**465**	3,7-Dihydroxy-2-[3-(4-hydroxy-3-methylbutyl)-4-methoxyphenyl]-5,6-dimethoxy-4H-1-benzopyran-4-one	[M+Cl]-	C23H26O8	465.13232							2.3E+04	-0.3				
**466**	PC(0:0/14:0)	[M-H]-	C22H46NO7P	466.29391			4.8E+04	-1.6								
**467**	Lupinisoflavone N	[M-H]-	C25H28O9	471.16621							1.9E+04	-0.3				
**468**	Parsonsine	[M+Cl]-	C23H37NO7	474.22706									2.4E+04	-1.4		
**469**	(-)-Asbestinine 2	[M-H]-	C28H44O6	475.30651			5.2E+04	0.0								
**470**	Bidenoside B	[M-H]-	C24H30O10	477.17567									6.2E+04	2.0		
**471**	PG(13:0/0:0)	[M+Cl]-	C19H39O9P	477.20257	3.5E+04	-0.1										
**472**	Guanosine diphosphate	[M+Cl]-	C10H15N5O11P2	477.99279					4.2E+04	2.0						
**473**	Delcorine	[M-H]-	C26H41NO7	478.28103			7.2E+04	1.0								
**474**	sn-3-O-(Geranylgeranyl)glycerol 1-phosphate	[M+Cl]-	C23H41O6P	479.23329									1.9E+04	0.4		
**475**	Ceramide (d18:1/12:0)	[M-H]-	C30H59NO3	480.44261					3.9E+04	-0.8	1.9E+04	-1.4				
**476**	Coenzyme M 7-mercaptoheptanoylthreonine-phosphate heterodisulfide	[M-H]-	C13H26NO10PS3	482.03910					6.3E+04	-1.5						
**477**	17beta-Estradiol 17-(beta-D-glucuronide)	[M+Cl]-	C24H31O8	482.17129			9.9E+04	1.3								
**478**	PA(20:4(5Z,8Z,11Z,14Z)e/2:0)	[M-H]-	C25H43O7P	485.26829							1.5E+06	-1.9	7.2E+05	0.0	3.4E+05	0.0
**479**	Acacetin 7-(2''-acetylglucoside)	[M-H]-	C24H24O11	487.12459			1.2E+05	-1.5								
**480**	Fucosyllactose	[M-H]-	C18H32O15	487.16744									2.9E+04	-1.2		
**481**	1-(5'-Phosphoribosyl)-5-amino-4-(N-succinocarboxamide)-imidazole	[M+Cl]-	C13H19N4O12P	489.04284							3.3E+04	0.6				
**482**	Deoxylimononic acid D-ring-lactone	[M-H]-	C26H34O9	489.21309					4.1E+04	-0.2						
**483**	PC(O-16:0/O-1:0)	[M-H]-	C25H54NO6P	494.36082							2.4E+04	1.6				
**484**	LysoPE(0:0/20:5(5Z,8Z,11Z,14Z,17Z))	[M-H]-	C25H42NO7P	498.26356											2.5E+04	-1.9
**485**	Pumilaisoflavone B	[M+Cl]-	C27H28O7	499.15227									2.0E+04	1.3		
**486**	6-Hydroxykaempferol 3,5,7,4'-tetramethyl ether 6-rhamnoside	[M-H]-	C25H28O11	503.15589	2.6E+04	0.7										
**487**	N-oleoyl tryptophan	[M+Cl]-	C29H44N2O3	503.30375									1.6E+04	1.7		
**488**	N3'-Acetyltobramycin	[M-H]-	C20H39N5O10	508.26154					3.3E+04	1.7						
**489**	PC(14:0/2:0)	[M-H]-	C24H48NO8P	508.30435											1.8E+04	0.3
**490**	Cer(d18:1/14:0)	[M-H]-	C32H63NO3	508.47412							1.7E+04	-1.2				
**491**	Flemiphyllin	[M+Cl]-	C30H34O5	509.21003	2.4E+04	0.3										
**492**	6'',6''-Dimethylpyraono[2'',3'':7,8]kaempferol 4'-methyl ether 3-rhamnoside	[M-H]-	C27H28O10	511.16035											1.9E+04	1.2
**493**	Raucaffricine	[M-H]-	C27H32N2O8	511.20814							2.2E+04	0.9				
**494**	Luteolin 7-(2''-glucosyllactate)	[M-H]-	C24H24O13	519.11396					3.9E+04	0.9						
**495**	Cer(d18:0/15:0)	[M-H]-	C33H67NO3	524.50427					4.0E+04	1.0						

#	Putative Annotation [a]	Ion	Formula	$m/z_{Theo.}$ [b]	R2020[c]	ppm	W2020[c]	ppm	B2020[c]	ppm	R2021[c]	ppm	W2021[c]	ppm	B2021[c]	ppm
**496**	Kaempferol 3,7,4'-tri-O-sulfate	[M-H]-	C15H10O15S3	524.91091	2.6E+04	-0.9										
**497**	Quercetin 3-rhamnoside-3'-sulfate	[M-H]-	C21H20O14S	527.05010			3.0E+05	1.4								
**498**	dTDP-L-olivose	[M-H]-	C16H26N2O14P2	531.07865	2.1E+04	-0.2										
**499**	13-Deoxycarminomycin	[M+Cl]-	C26H29NO9	534.15328											2.0E+04	0.7
**500**	Lappaol A	[M-H]-	C30H32O9	535.19736	6.1E+04	-0.2										
**501**	Phytofluene	[M-H]-	C40H62	541.47784							2.3E+04	0.1				
**502**	2,5-Diaminopyrimidine nucleoside triphosphate	[M+Cl]-	C9H18N5O14P3	547.97622					3.9E+04	-0.9						
**503**	Phoslactomycin B	[M+Cl]-	C25H40NO8P	548.21866									2.1E+04	-0.2		
**504**	Quercetin 3-sulfate-7-alpha-arabinopyranoside	[M+Cl]-	C20H18O14S	549.01038											2.1E+04	1.4
**505**	all-trans-retinyl stearate	[M-H]-	C38H64O2	551.48335			3.7E+05	0.3								
**506**	Pantethine	[M-H]-	C22H42N4O8S2	553.23713	2.4E+04	-1.8										
**507**	PI(P-16:0/0:0)	[M-H]-	C25H49O11P	555.29397			2.8E+06	0.9								
**508**	Cucurbitacin E	[M-H]-	C32H44O8	555.29634			2.8E+06	1.6								
**509**	Cucurbitacin P	[M+Cl]-	C30H48O7	555.31034							2.7E+04	-1.7				
**510**	Linoleoylglycerophosphocholine	[M+Cl]-	C26H51NO7P	555.31034							2.7E+04	-1.1				
**511**	5,7,2',3',4'-Pentahydroxy-3,6-dimethoxyflavone 7-glucoside	[M+Cl]-	C23H24O14	559.08601	2.9E+04	-0.5										
**512**	PG(P-20:0/0:0)	[M+Cl]-	C26H53O8P	559.31735									2.4E+04	-0.3		
**513**	3-Hydroxy-L-tyrosyl-AMP	[M+Cl]-	C19H23N6O10P	561.09073			1.9E+05	1.7								
**514**	Musaroside	[M-H]-	C30H44O10	563.28617			1.2E+05	-0.5								
**515**	N-[(3a,5b,7b)-7-hydroxy-24-oxo-3-(sulfoxy)cholan-24-yl]-Glycine	[M+Cl]-	C26H43NO8S	564.24034	2.4E+04	-1.4										
**516**	Ginkgetin	[M-H]-	C32H22O10	565.11402			9.2E+04	1.0								
**517**	dTDP-L-olivose	[M+Cl]-	C16H26N2O14P2	567.05533	4.7E+04	-1.3										
**518**	Apigenin 7-methyl ether 5-(6''-malonylglucoside)	[M+Cl]-	C25H24O13	567.09088									3.4E+04	0.4		
**519**	Corotoxigenin-3-o-alpha-L-rhamnopyranoside	[M+Cl]-	C29H42O9	569.25228	3.3E+04	-0.3										
**520**	PS(22:4(7Z,10Z,13Z,16Z)/0:0)	[M-H]-	C28H48NO9P	572.29924											3.4E+04	0.3
**521**	Cer(d18:1/19:0)	[M-H]-	C37H73NO3	578.55105											4.9E+04	1.2
**522**	Vitamin K2	[M-H]-	C41H56O2	579.41991											2.8E+04	1.5
**523**	Dihydrozeatin-9-N-glucoside-O-glucoside	[M+Cl]-	C22H35N5O11	580.20307							1.9E+04	-0.6				
**524**	Cer(t18:0/16:0)	[M+Cl]-	C34H69NO4	590.49206			1.3E+05	1.0								
**525**	Ceramide (d18:1/20:0)	[M-H]-	C38H75NO3	592.56705							2.0E+04	0.6	3.1E+04	1.5		
**526**	Dalpanol O-glucoside	[M+Cl]-	C29H34O12	609.17443	2.5E+04	0.3										
**527**	DG(15:0/18:4(6Z,9Z,12Z,15Z)/0:0)	[M+Cl]-	C36H62O5	609.42987											2.8E+04	-1.2
**528**	Myricetin 3-sambubioside	[M-H]-	C26H28O17	611.12457					3.0E+04	1.3						
**529**	Naringenin 7-(2-p-Coumaroylglucoside)	[M+Cl]-	C30H28O12	615.12748			8.4E+04	2.8								
**530**	Apigenin 5-rhamnosyl-(1->2)-(6''-acetylglucoside)	[M-H]-	C29H32O15	619.16661											2.8E+04	0.4
**531**	Cer(d18:0/22:0)	[M-H]-	C40H81NO3	622.61437			5.2E+04	-2.0								
**532**	Glycochenodeoxycholic acid 3-glucuronide	[M-H]-	C32H51NO11	624.33942					7.9E+04	-0.8						
**533**	Ubiquinone-6	[M+Cl]-	C39H58O4	625.40219											3.2E+04	1.2

#	Putative Annotation [a]	Ion	Formula	m/z $_{Theo.}$ [b]	R2020[c]	ppm	W2020[c]	ppm	B2020[c]	ppm	R2021[c]	ppm	W2021[c]	ppm	B2021[c]	ppm
**534**	Quercetin 3-(4''-acetylrhamnoside)-7-rhamnoside	[M-H]-	C29H32O16	635.16251					3.9E+04	-1.2						
**535**	Cer(t18:0/24:0)	[M-H]-	C42H85NO4	666.64024							2.1E+04	0.5				
**536**	Acacetin 7-(4'''-acetylrutinoside)	[M+Cl]-	C30H34O15	669.15973					2.8E+04	-0.8						
**537**	PE(14:1(9Z)/P-18:1(11Z))	[M-H]-	C37H70NO7P	670.48163							3.2E+04	0.1				
**538**	Purpureacin-1	[M+Cl]-	C37H66O8	673.44517	4.1E+04	0.7										
**539**	Gnididin	[M+Cl]-	C37H44O10	683.26330									1.9E+04	-0.7		
**540**	DG(20:5(5Z,8Z,11Z,14Z,17Z)/22:6(4Z,7Z,10Z,13Z,16Z,19Z)/0:0)	[M-H]-	C45H66O5	685.48341					3.9E+04	0.5						
**541**	PG(14:0/16:0)	[M-H]-	C36H71O10P	693.47087									3.4E+05	0.5		
**542**	Taurolithocholic acid 3-glucuronide	[M+Cl]-	C32H53NO11S	694.30269									4.4E+04	0.9		
**543**	Ecdysone palmitate	[M-H]-	C43H74O7	701.53534							2.4E+04	1.2				
**544**	CE(19:0)	[M+Cl]-	C46H82O2	701.60088			4.1E+04	1.1								
**545**	PA(16:1(9Z)/22:4(7Z,10Z,13Z,16Z))	[M-H]-	C41H71O8P	721.48213									3.0E+04	-1.0		
**546**	Kaempferol 3-rhamnosyl-(1->4)-rhamnoside-7-rhamnoside	[M-H]-	C33H40O18	723.21460					3.6E+04	-0.6						
**547**	Naringenin 7-O-(2'',6''-di-O-alpha-rhamnopyranosyl)-beta-glucopyranoside	[M-H]-	C33H42O18	725.22936							3.5E+04	0.7				
**548**	PG(12:0/18:2(9Z,12Z))	[M+Cl]-	C36H67O10P	725.41754					5.6E+04	-1.3						
**549**	Ubiquinone-8	[M-H]-	C49H74O4	725.55191									3.6E+04	-0.7		
**550**	DG(19:0/22:2(13Z,16Z)/0:0)[iso2]	[M+Cl]-	C44H82O5	725.58563			1.0E+05	-1.9								
**551**	Scillarenin 3-O-glucosylrhamnoside	[M+Cl]-	C36H52O13	727.31118					1.6E+05	-1.4						
**552**	PG(13:0/20:4(5Z,8Z,11Z,14Z))	[M-H]-	C39H69O10P	727.45651							1.6E+05	-1.3				
**553**	PA(17:0/21:0)	[M-H]-	C41H81O8P	731.55963	5.7E+04	-0.3										
**554**	PC(16:0/16:0)	[M-H]-	C40H80NO8P	732.55538									2.3E+04	-0.7		
**555**	PE(O-16:1(1Z)/22:6(4Z,7Z,10Z,13Z,16Z,19Z))]	[M-H]-	C43H74NO7P	746.51325											4.9E+04	-0.3
**556**	19'-Hexanoyloxyisomytiloxanthin	[M+Cl]-	C46H64O6	747.43969			4.2E+04	0.5								
**557**	UDP-N-acetylmuramoyl-L-alanine	[M-H]-	C23H36N4O20P2	749.13254	7.2E+04	-0.8										
**558**	Luteolin 7-glucuronide-3',4'-dirhamnoside	[M-H]-	C33H38O20	753.18916					5.2E+04	-1.1						
**559**	SM(d18:0/20:2(11Z,14Z))	[M-H]-	C43H83N2O6P	753.59160			3.4E+04	1.4								
**560**	PA(16:0/22:1(11Z))	[M+Cl]-	C41H79O8P	765.52066	7.0E+04	0.1										
**561**	PC(O-16:0/O-18:1(9Z))	[M+Cl]-	C42H86NO6P	766.58966											3.0E+04	-1.3
**562**	PC(15:0/20:3(5Z,8Z,11Z))	[M-H]-	C43H80NO8P	768.55557											3.0E+04	-0.9
**563**	PA(20:3(8Z,11Z,14Z)/22:6(4Z,7Z,10Z,13Z,16Z,19Z))	[M-H]-	C45H71O8P	769.48176											3.1E+04	-0.5
**564**	1-(6-[3]-ladderane-hexanoyl)-2-(8-[3]-ladderane-octanyl)-sn-glycero-3-phospho-(1'-sn-glycerol)	[M-H]-	C44H73O9P	775.49259											2.6E+04	-0.8
**565**	Patellamide A	[M+Cl]-	C35H50N8O6S2	777.29953											1.3E+05	-0.8
**566**	Cyanidin 3,5-di-(6-malonylglucoside)	[M-H]-	C33H35O22	782.15472			3.0E+04	-0.6								
**567**	Pfaffoside A	[M+Cl]-	C40H60O13	783.37374					3.0E+04	-1.2						
**568**	PI(P-16:0/16:1(9Z))	[M-H]-	C41H77O12P	791.50893					3.0E+04	-1.2						
**569**	Delphinidin 3-(2-xylosylgalactoside)-5-glucoside	[M+Cl]-	C32H39O21	794.16791							2.7E+04	-0.2				

#	Putative Annotation [a]	Ion	Formula	m/z $_{Theo.}$ [b]	R2020 [c]	ppm	W2020 [c]	ppm	B2020 [c]	ppm	R2021 [c]	ppm	W2021 [c]	ppm	B2021 [c]	ppm
**570**	PA(20:4(5Z,8Z,11Z,14Z)/22:6(4Z,7Z,10Z,13Z,16Z,19Z))	[M+Cl]-	C45H69O8P	803.44198											4.3E+04	0.5
**571**	PS(13:0/22:4(7Z,10Z,13Z,16Z))	[M+Cl]-	C41H72NO10P	804.45879			2.5E+04	-0.8								
**572**	PG(O-18:0/21:0)	[M-H]-	C45H91O9P	805.63279			2.4E+04	0.5								
**573**	PG(17:0/22:6(4Z,7Z,10Z,13Z,16Z,19Z))	[M-H]-	C45H77O10P	807.51816			2.0E+04	-2.0								
**574**	PG(15:0/22:4(7Z,10Z,13Z,16Z))	[M+Cl]-	C43H77O10P	819.49529									1.9E+04	-0.6		
**575**	PG(17:0/22:0)	[M-H]-	C45H89O10P	819.61206			9.2E+04	-1.5								
**576**	PG(O-18:0/20:3(8Z,11Z,14Z))	[M+Cl]-	C44H83O9P	821.54601							3.3E+04	1.1				
**577**	5-Methyl-5,6,7,8-tetrahydromethanopterin	[M+Cl]-	C31H47N6O16P	825.24802			2.1E+04	0.6								
**578**	PG(18:1(9Z)/22:2(13Z,16Z))	[M-H]-	C46H85O10P	827.58076	3.6E+04	0.5										
**579**	PG(16:0/22:1(11Z))	[M+Cl]-	C44H85O10P	839.55744			2.9E+04	2.4								
**580**	Bayogenin 3-O-cellobioside	[M+Cl]-	C42H68O15	847.42499											1.7E+04	0.3
**581**	PS(O-20:0/22:6(4Z,7Z,10Z,13Z,16Z,19Z))	[M-H]-	C48H84NO9P	848.58109			3.3E+04	-2.1								
**582**	Palargonidin 3-(6''-ferulylglucoside)-5-(6'''-malonylglucoside)	[M-H]-	C40H41O21	856.20695											2.4E+04	-0.2
**583**	PS(O-18:0/22:4(7Z,10Z,13Z,16Z))	[M+Cl]-	C46H84NO9P	860.55854							4.4E+04	-0.9				
**584**	PC(18:0/22:6(4Z,7Z,10Z,13Z,16Z,19Z))	[M+Cl]-	C48H84NO8P	868.56286			2.0E+04	2.2								
**585**	PI(O-18:0/20:5(5Z,8Z,11Z,14Z,17Z))	[M-H]-	C47H83O12P	869.55453											1.8E+04	0.5
**586**	PG(19:0/22:4(7Z,10Z,13Z,16Z))	[M+Cl]-	C47H85O10P	875.55744			2.0E+04	-2.5								
**587**	Guanosine tetraphosphate adenosine	[M+Cl]-	C20H28N10O20P	887.01258			2.9E+04	2.2								
**588**	PC(20:2(11Z,14Z))	[M+Cl]-	C50H84NO8P	892.56338							4.8E+04	-0.6				
**589**	Pelargonidin 3-glucoside-7-(6-(4-(glucosyl)-p-hydroxybenzoyl)glucoside)	[M+Cl]-	C40H45O22	912.20966									4.8E+04	0.0		
**590**	4-Isopropenyl-2-oxy-cyclohexanecarboxyl-CoA	[M-H]-	C31H48N7O18P3	930.19201									2.0E+04	-0.4		
**591**	TG(17:2(9Z,12Z)/20:5(5Z,8Z,11Z,14Z,17Z)/20:5(5Z,8Z, 11Z,14Z,17Z))	[M+Cl]-	C60H92O6	943.65879			3.1E+04	-2.2								
**592**	Pentaglutamyl folate	[M-H]-	C39H47N11O18	956.30308					3.4E+04	-0.3						
**593**	PI(20:1(11Z)/22:4(7Z,10Z,13Z,16Z))	[M+Cl]-	C51H89O13P	975.57287					3.7E+04	0.6						
**594**	Isorhamnetin 3-rhamnosyl-(1->2)-gentiobiosyl-(1->6)-glucoside	[M+Cl]-	C40H52O26	983.24408	6.3E+04	-0.4										

[a] CE Cholesteryl ester; Cer Ceramide; DG Diacylglycerol; MG Monoacylglycerol; PA Phosphatidic acid; PC Phosphatidylcholine; PE Phosphatidylethanolamine; PG Glycerophospholipids; PGE1 Prostaglandin E1; PI Phosphatidylinositol; PS Phosphatidylserine; SM Sphingomyelin; TG Triacylglycerol.

[b] Theoretical mass

[c] R stands for red tunica, W stands for white tunica and B stands for bulb.
